# A positive feedback loop between germ cells and gonads induces and maintains sexual reproduction in a cnidarian

**DOI:** 10.1126/sciadv.adq8220

**Published:** 2025-01-08

**Authors:** Camille Curantz, Ciara Doody, Helen R. Horkan, Gabriel Krasovec, Paris K. Weavers, Timothy Q. DuBuc, Uri Frank

**Affiliations:** ^1^ Centre for Chromosome Biology, School of Biological and Chemical Sciences, University of Galway, Galway H91 W2TY, Ireland.; ^2^ Université Paris Cité, CNRS, Institut Jacques Monod, Paris F-75013, France.; ^3^ Department of Biology, Queens College, The City University of New York, 6530 Kissena Blvd., Flushing, NY 11367, USA.; ^4^ Biology and Biochemistry PhD Programs, CUNY Graduate Center, 365 5th Ave., New York, NY 10016, USA.

## Abstract

The fertile gonad includes cells of two distinct developmental origins: the somatic mesoderm and the germ line. How somatic and germ cells interact to develop and maintain fertility is not well understood. Here, using grafting experiments and transgenic reporter animals, we find that a specific part of the gonad—the germinal zone—acts as a sexual organizer to induce and maintain de novo germ cells and somatic gonads in the cnidarian *Hydractinia symbiolongicarpus*. Germ cells express a member of the transforming growth factor–β family,* Gonadless* (*Gls*), that induces gonad morphogenesis. Loss of *Gls* resulted in animals lacking gonads but having nonproliferative germ cells. We propose that primary germ cells drive gonad development though Gls secretion. The germinal zone in the newly formed gonad provides positive feedback to induce secondary germ cells by activating *Tfap2* in resident pluripotent stem cells. The contribution of germ cell signaling to the patterning of somatic gonadal tissue may be a general animal feature.

## Teaser

Cnidarian germ cells secrete TGF-β to induce gonad development, and gonads induce germ cells by activating *Tfap2* in stem cells.

## INTRODUCTION

Jointly, germ cells and somatic gonadal cells constitute the main reproductive organ. Their respective developmental origin, however, is spatially and temporally distinct. In most animals, germ cells are generated only once in a lifetime during early embryonic development (*1*, *2*). Somatic gonad tissues develop later and are derived from mesoderm. Once specified, primordial germ cells migrate to the region of developing gonads where they are incorporated with somatic gonad cells (*2*, *3*). Defects in integration result in impaired fertility (*4*). Somatic gonad cells communicate with germ cells using multiple signaling pathways to regulate gamete formation (*3*). However, the nature of the signals from germ cells to the gonad remains poorly understood. Numerous studies propose that germ cells are dispensable for early embryonic gonad development in mammals but that they are essential later for its proper maturation (*5*). In zebrafish, gonads fail to regenerate in the absence of germ cells (*6*). In *Drosophila*, germ cells control the differentiation, proliferation, and morphogenesis of the somatic follicle cells that surround them by activating the delta/notch signaling pathway (*7*). However, data are often contradictory between male and female or across different species of vertebrates and invertebrates. Furthermore, these studies rely on the ablation of germ cells before their integration in the gonad; this is difficult to perform in animals with maternal germ cell induction. Therefore, the potential implication of an early signaling of germ cells to the gonad somatic tissue to regulate their morphogenesis remains a possibility that requires further studies using other animals (*5*). Overall, these processes, being restricted to early embryogenesis, are difficult to access and manipulate in most animal models. To address this issue, we have studied germ cell and soma interactions in the developing and mature gonads of the cnidarian *Hydractinia symbiolongicarpus*, an animal that continuously develops new gonads and induces new germ cells from pluripotent stem cells (*8*).

**Fig. 1.**
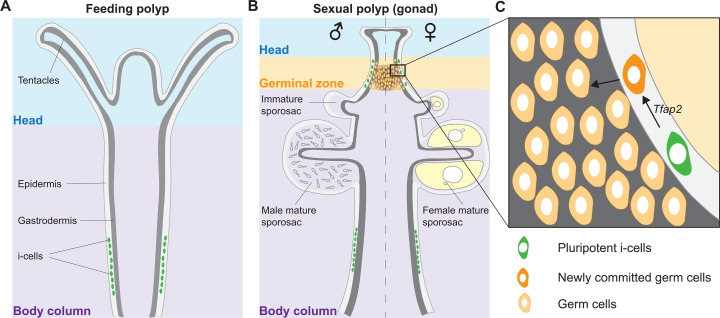
*Hydractinia* sexual development. (**A**) Feeding polyp morphology showing i-cell localization (green) in the epidermis of the lower part of the body column. (**B**) Sexual polyp morphology showing germ cells (orange) and their commitment from i-cells in the germinal zone. Once committed, germ cells become gastrodermal and start their maturation into functional gametes. The left side represents a male and the right side a female. (**C**) Close-up of the germinal zone [black rectangle in (B)] showing i-cells being induced to germ cell fate by Tfap2 expression.

*H. symbiolongicarpus* is a clonal and colonial cnidarian, a relative of jellyfishes and corals (*9*). A *Hydractinia* colony is either male or female and is structured as a network of substratum-attached gastrovascular tubes (called stolons) from which zooids (called polyps) bud continuously as the stolons elongate. The colony contains two primary types of polyps. The first polyp to emerge during metamorphosis of the sexually derived larva is of the feeding type. These polyps have a head with a mouth surrounded by muscular and innervated tentacles, used to catch prey (Fig. 1A). About 2 months later, when the colony reaches some 100 feeding polyps, a new type of polyp emerges—the sexual polyp. Sexual polyps are the animal’s gonads (*10*), constituting 20 to 70% of all polyps in adult colonies (table S1). The body column of sexual polyps lacks a functional mouth and has only rudimentary tentacles around its oral end. They obtain food through the shared stolonal network. The region below the sexual polyp head (the “neck”) is called the germinal zone (Fig. 1B). Here, adult pluripotent stem cells [known as i-cells (*8*, *11*)] are induced to germ cell fate by activation of *Tfap2*, the master regulator of germ cell commitment (Fig. 1C) (*10*). They then migrate to the gastrodermis, proliferate, and move into containers, known as sporosacs, where they mature to gametes (Fig. 1B). Stolonal elongation, new polyp budding (both feeding and sexual), and germ cell induction from pluripotent i-cells occur continuously following sexual maturation, making all stages of sexual development available at any one time (*10*).

## RESULTS

### The germinal zone is a sexual organizer

**Fig. 2.**
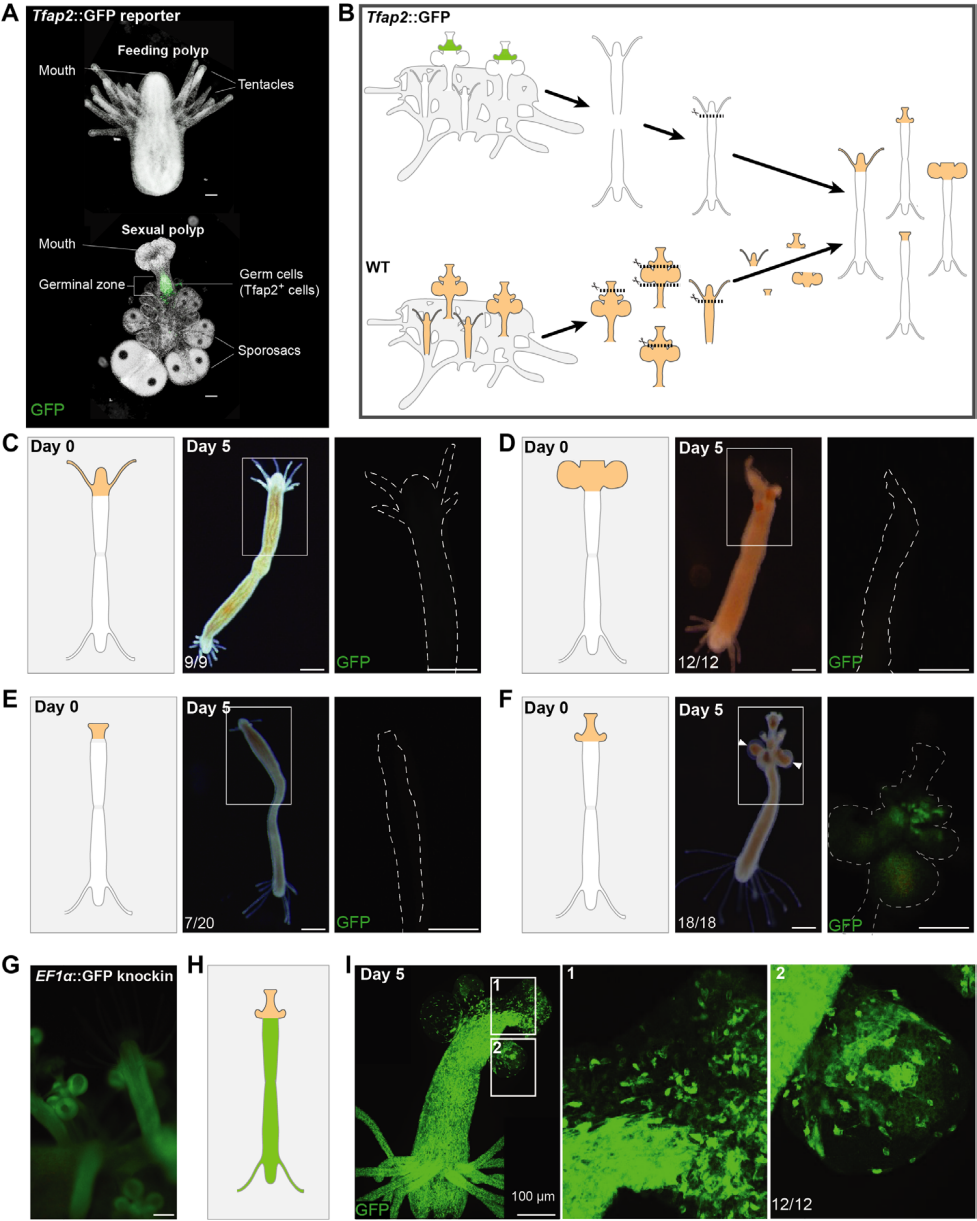
De novo induction of germ cells and somatic gonad tissue upon heterotopic grafting of gonad tissue onto feeding polyps. (**A**) *Tfap2*::GFP reporter animal. (**B**) Cartoon of the experimental grafting procedure showing different parts of sexual polyps grafted to a feeding polyp body column. WT, wild type. (**C** to **F**) Outcomes 5 days after grafting of feeding head grafting (C); sporosac region without germinal zone grafting (D); oral tip only (E); head including germinal zone graft showing induction of new germ cells (GFP^+^) in the recipient feeding polyp (F). White arrows show developing sporosacs in (F). White dashed line shows the outline of the polyp. (**G**) An *Ef1a*::GFP knock-in animal with ubiquitous GFP expression. (**H**) Cartoon of a chimera between a wild-type germinal zone (orange) and an *Ef1a*::GFP knock-in feeding polyp (green) at day 0 after grafting. (**I**) Confocal images of a representative chimera at day 5 after grafting. White rectangles show close-up views of germinal zone (1) and sporosac (2), both composed of a mixture of GFP^+^ and GFP^−^ cells. Scale bar, 100 μm.

To study the maintenance of sexual tissue in *Hydractinia*, we performed regeneration experiments of feeding and sexual polyps in a *Tfap2::GFP* transgenic reporter animal. *Tfap2* is an early germ cell marker; it drives the commitment of pluripotent i-cells to germ cell fate. Feeding polyps of *Tfap2::GFP* reporter animals have no green fluorescent protein (GFP) fluorescent cells, while in their sexual polyps, all early germ cells are GFP^+^. Fluorescence dissipates as early germ cells mature to gametes, reflecting *Tfap2* expression in early but not late germ cells (*10*) (Fig. 2A).

Removing the head of feeding polyps results in regeneration of a new feeding head from the stump within 3 days (*12*, *13*). Amputation of sexual heads, however, resulted in various outcomes, depending on the site of amputation. Amputating only the most distal (oral) part, leaving the germinal zone intact, resulted in the regeneration of a new sexual head (*n* = 69 of 75). By contrast, if the entire oral part including the germinal zone were amputated, either a feeding head regenerated (*n* = 11 of 75) or no head regenerated at all (*n* = 64 of 75). In both cases, all sexual features degenerated (fig. S1). This suggests a pivotal role for the germinal zone in the maintenance of gonadal tissue identity.

Next, we studied the induction of sexual development. To do so, we conducted a series of experiments by heterotopic grafting of gonadal tissue onto feeding polyps (Fig. 2B). We first grafted together two feeding polyps of the *Tfap2* reporter animal by joining their cut aboral ends, resulting in a biheaded animal. This allowed one head to feed after removal of the other. We amputated one of the heads and replaced it with a new feeding head from a wild-type donor. This resulted in the maintenance of the feeding identity of the chimeric animal with neither induction of somatic sexual structures (by morphology) nor germ cells (by lack of GFP) (Fig. 2C).

We then replaced one of the feeding heads with a piece of the lower body of a wild-type sexual polyp (i.e., a gonad) that contained immature sporosacs but no germinal zone. Normally, these sporosacs mature within days and spawn. However, the grafted sporosacs did not mature and gradually degenerated, nor did GFP^+^ cells appear, indicating an absence of somatic gonad development and of germ cell induction (Fig. 2D). Next, we replaced one of the feeding heads with the oral tip of a wild-type sexual polyp, again excluding the germinal zone. Within days, we observed in about half of them the maintenance of the sexual head but no new sexual tissue was induced. In the other half, the sexual head was resorbed, and a new feeding head grew instead. In both cases, no GFP^+^ germ cells were induced (Fig. 2E).

Last, when the germinal zone was included in the donor tissue, the sexual identity was maintained and new sporosacs developed. Moreover, de novo germ cells were induced in the recipient feeding polyp, attested by GFP expression (Fig. 2F), showing that the germinal zone can not only maintain the sexual identity of the entire somatic gonad but also induce new germ cells. To identify possible induction of new somatic gonad tissue by the grafted germinal zone, we repeated the experiment using a different transgenic animal as recipient. This animal had GFP knocked into its *Ef1a* gene (*14*), resulting in ubiquitous GFP fluorescence (Fig. 2G). We grafted a sexual head including the germinal zone from a wild-type animal onto an *Ef1a* knock-in animal (Fig. 2H). This resulted in the induction of both germ cells and somatic gonads in the recipient feeding polyp (Fig. 2, F and I).

Overall, the above experiments show that the germinal zone acts as a sexual organizer, being sufficient to induce and maintain sexual tissue that includes both somatic and germ cells in nonsexual tissue. The results also demonstrate that in the absence of a germinal zone, the feeding identity of polyps predominates, being the default fate of *Hydractinia* polyp development.

### *Gonadless* is a germ cell–specific member of the transforming growth factor–β family

To gain insight into the mechanisms of sexual induction, we reanalyzed a published differential gene expression dataset of different polyp types (*10*). A gene, annotated as *Dvr1* in GenBank (accession no. HyS0012.146), is up-regulated in the distal part of male and female sexual polyps. Phylogenetic analysis placed the gene in the transforming growth factor–β (TGF-β) protein family that includes bone morphogenetic protein (BMP), decapentaplegic-Vg-related (DVR), growth and differentiation factor (GDF), and NODAL proteins, but its orthologous relationship with vertebrate subgroup members could not be resolved (fig. S2). The TGF-β pathway is a major regulator of germ cell induction, maintenance, proliferation, and maturation in bilaterians (*3*). The predicted protein has a secretory signal peptide in its N terminus and a TGF-β domain in the C-terminal region (fig. S2). We hypothesized that this gene is involved in sexual development. For reasons outlined below, we named the gene *Gonadless* (*Gls*).

**Fig. 3.**
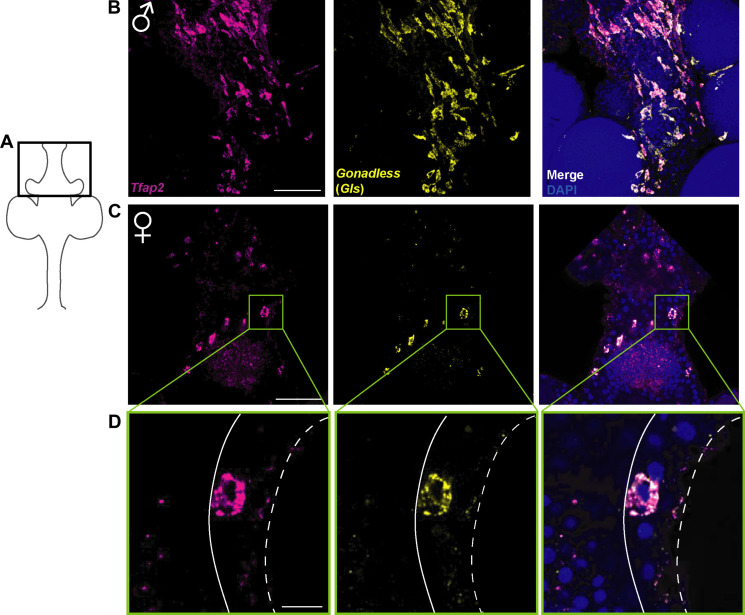
*Gls* expression pattern. (**A**) Cartoon of a sexual polyp. The black box corresponds to the position of the confocal images. (**B**) Maximum projection of mRNA in situ hybridization of *Tfap2* and *Gls* in a male sexual polyp. DAPI, 4′,6-diamidino-2-phenylindole. (**C**) Maximum projection of mRNA in situ hybridization of *Tfap2* and *Gls* in a female sexual polyp. Scale bar, 50 μm. Green rectangle corresponds to the close-up view shown in (D). (**D**) Single confocal section showing expression of *Tfap2* and *Gls* in the cells boxed in (C). White dashed line represents the outline of the epidermis, and continuous white line represents the basement membrane (mesoglea) separating the gastrodermis from the epidermis. Scale bar, 10 μm.

We performed signal amplification by exchange reaction (SABER) single-molecule fluorescence mRNA in situ hybridization (*15*, *16*) to study the expression pattern of *Gls*. These experiments showed that *Gls* mRNA is present at detectable levels exclusively in germ cells in the germinal zone that also express *Tfap2*, an early germ cell marker (*10*) (Fig. 3 and fig. S3). Tfap2 is the master regulator of germ cell commitment, essential and sufficient to induce germ cell fate in pluripotent i-cells (*10*). As shown previously, loss of *Tfap2* results in animals that have neither germ cells nor gonads (*10*).

### Gls is required for gonad development

**Fig. 4.**
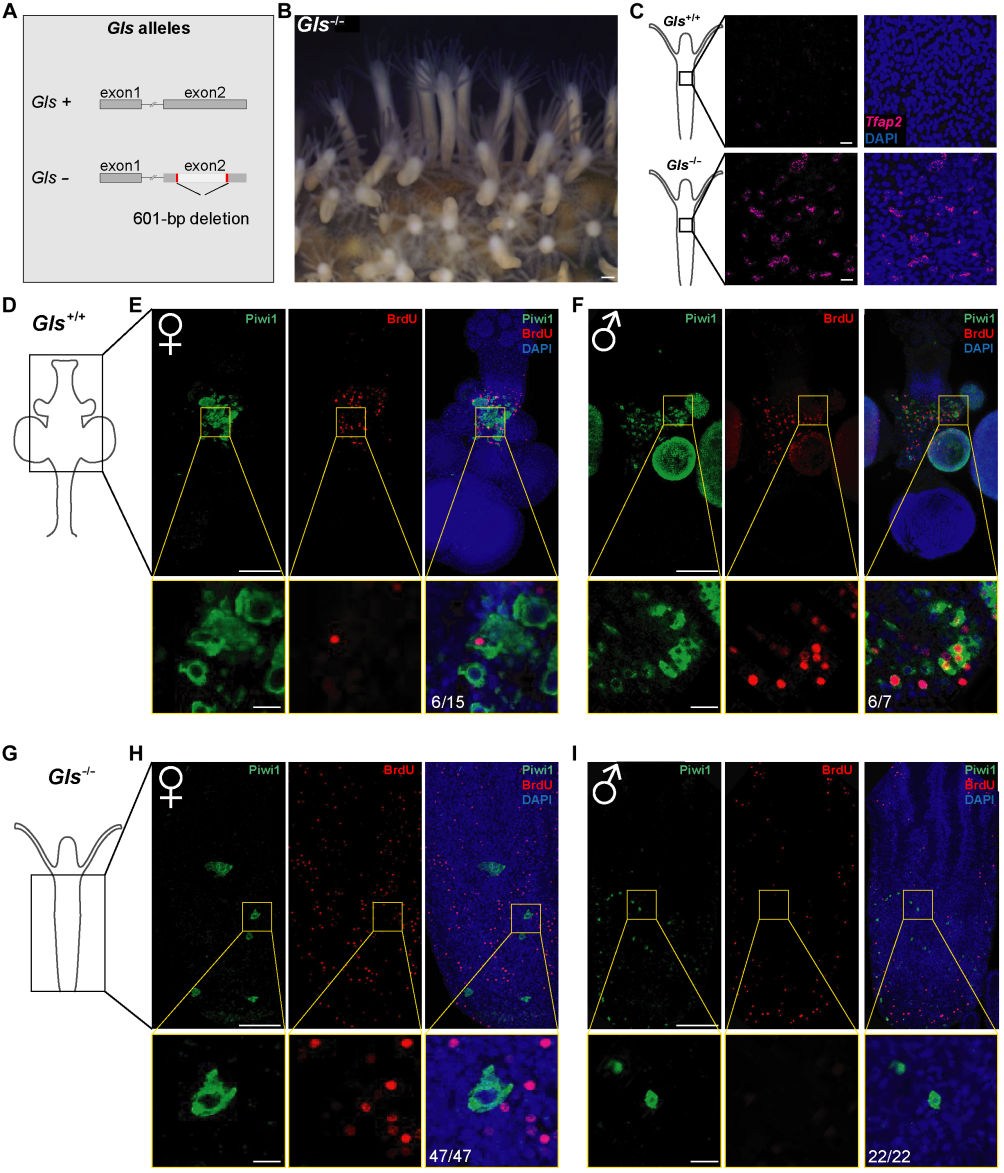
Morphological and cellular characterization of *Gls*^−/−^ animals. (**A**) Graphical representation of the genomic structure of wild-type and mutant alleles of *Gls*. (**B**) A *Gls*^−/−^ mutant, having only feeding polyps. (**C**) Maximum projection of mRNA in situ hybridization of *Tfap2* in wild-type (upper) and *Gls*^−/−^ (lower) feeding polyps. *Tfap2*^+^ germ cells are present in the mutant but not in the wild-type feeding polyp. (**D**) Cartoon of a wild-type sexual polyp. The black box corresponds to the position of the confocal images shown in (E) and (F). (**E** and **F**) Maximum projection of immunostaining of Piwi1 (green) and BrdU (red) in the gastrodermis of a female (E) and a male (F). Yellow boxes represent a close-up view of a single confocal section shown below, respectively. Germ cells proliferate in wild-type animals. (**G**) Cartoon of a Gls^−/−^ feeding polyp. The black box corresponds to the position of the confocal images shown in (H) and (I). (**H** and **I**) Maximum projection of immunostaining for Piwi1 (green) and BrdU (red) in the gastrodermis of a female mutant (H) or of a male mutant polyp. Yellow boxes represent a close-up of a single confocal section showing that germ cells do not proliferate in *Gls*^−/−^ mutants. Scale bar, 100 μm. Scale bar in the boxes, 10 μm.

To address the function of Gls, we generated knockout animals using CRISPR-Cas9–mediated mutagenesis. Two single guide RNAs (sgRNAs) were designed to flank the TGF-β domain of *Gls* (Fig. 4A). We injected the sgRNAs together with recombinant Cas9 into zygotes and screened the G_0_ animals by polymerase chain reaction (PCR) to identify individuals that had a genomic deletion in their cells. We grew the animals to sexual maturity and crossed them with wild-type animals. Heterozygous G_1_ animals were selected by PCR and sequencing, grown to sexual maturity, and interbred to obtain homozygous *Gls*^−/−^ knockout animals. We obtained a total of eight knockout animals consistent with the predicted Mendelian distribution (fig. S4). These animals developed normally to planula larvae and metamorphosed to primary polyps, 24 hours after CsCl induction (*17*). The animals developed to normal-appearing young colonies; however, none of them developed sexual polyps (i.e., gonads) even after 20 months (Fig. 4B). Sexual polyps normally appear within 2 months in wild-type *Hydractinia* colonies (*18*). We concluded that Gls is essential to induce somatic gonad development (hence the name *Gonadless*).

### Gls is dispensable for primary germ cell induction

To gain insight into the cascade of sexual development, we looked for the presence of germ cells in *Gls*^−/−^ animals. We argued that if Gls acted directly upstream of *Tfap2*—the major regulator of germ cell fate—then no germ cells should be present in *Gls* mutants. By contrast, the presence of germ cells in *Gls* mutants would be consistent with the gene being expressed downstream of Tfap2. For this, we performed *Tfap2* mRNA in situ hybridization with mutant and wild-type feeding polyps and found expression only in mutants (Fig. 4C). We also performed immunofluorescence (IF) using anti-Piwi1 antibodies on wild-type and *Gls* knockout feeding polyps. Piwi1 is a marker of both pluripotent i-cells and germ cells. However, i-cells are only found in the interstitial spaces of the epidermis; they migrate soon after acquisition of germ cell fate to the gastrodermis where they mature to gametes. Therefore, epidermal Piwi1^+^ cells are mostly i-cells, but Piwi1^+^ cells in the gastrodermis are exclusively germ cells.

In wild-type feeding polyps, we did not find gastrodermal Piwi1^+^ cells (i.e., germ cells), except for one female polyp with a gastrodermal oocyte (fig. S5). However, in *Gls*^−/−^ feeding polyps, we found gastrodermal Piwi1^+^ cells in ~34% in of the feeding polyps surveyed (fig. S5). A 5-bromo-2′-deoxyuridine (BrdU) incorporation followed by anti-BrdU and anti-Piwi1 immunofluorescence showed that these germ cells were not proliferative, in contrast to proliferative germ cells in wild-type animals (Fig. 4, D to I, and table S2). In one of the mutant clones, the gastrodermal Piwi1^+^ cells were oocytes. The other mutants had small Piwi^+^ germ cells in their gastrodermis, leading us to believe that they were males (fig. S5). No mature eggs or sperm were found in *Gls*^−/−^ animals. Therefore, while Gls is required for the development of the somatic tissue of the gonad and, indirectly, for germ cell proliferation and maturation, it is not necessary for primary germ cell induction.

### Gls is essential for secondary germ cell induction

**Fig. 5.**
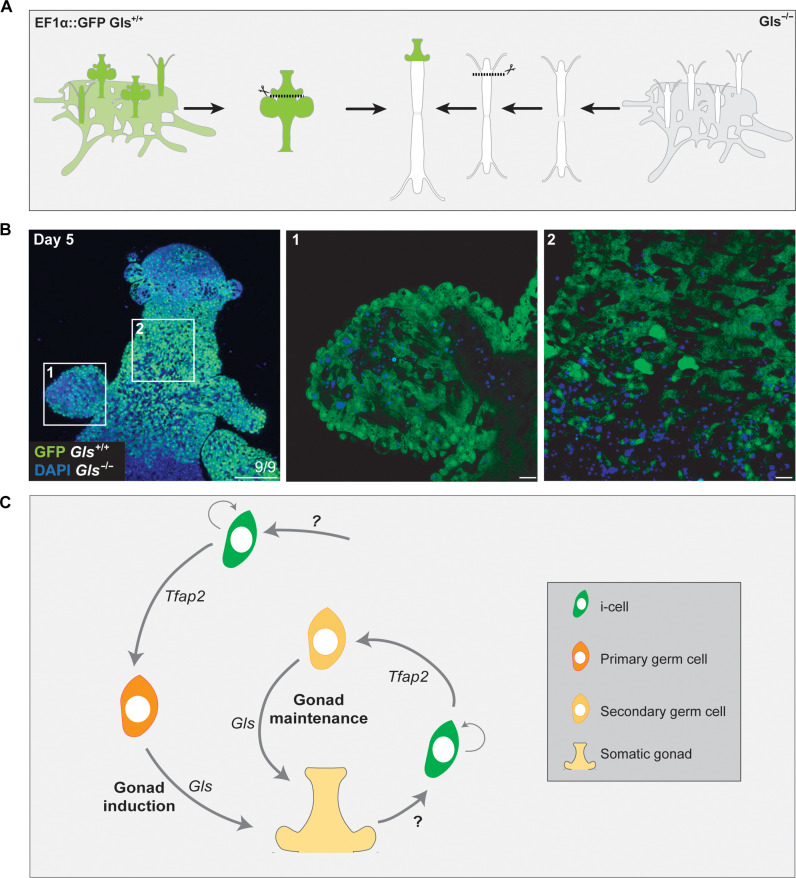
Heterotopic grafting of *Ef1a::*GFP fluorescent *Gls*^+/+^ sexual tissue onto sterile Gls^−/−^ animals. (**A**) Cartoon of the experimental grafting procedure. (**B**) Confocal images of a chimera, 5 days after grafting. Scale bar, 100 μm. White squares represent closes-up images of a newly formed sporosac (1) and the germinal zone (2), showing that they are composed of a mix of *Gls*^−/−^ (GFP^−^) and *Gls*+/+ (GFP^+^) cells. Scale bar, 10 μm. (**C**) A model for *Hydractinia* sexual development. Germ cells induce somatic gonad morphogenesis via Gls signaling, and the newly formed tissue, in turn, promotes secondary germ cell induction (by activating *Tfap2* in pluripotent stem cells), proliferation, and maturation.

To rescue the sexual phenotype in *Gls* mutants, we grafted a sexual head including the germinal zone from a *Gls* wild type, *Ef1a* knock-in animal with ubiquitous GFP expression, onto a decapitated *Gls*^−/−^ knockout feeding polyp (Fig. 5A). This experiment allowed the reintroduction of Gls signaling in the mutant and a direct observation of the outcome by fluorescence microscopy because the mutant cells were not fluorescent. It resulted in the induction of both somatic gonads and germ cells in the mutant, based on their morphology and localization, respectively (Fig. 5B), showing that a cascade driven by wild-type *Gls* expression is sufficient to induce full sexual development, including somatic gonadal tissue and secondary germ cells.

### Gls is insufficient for precocious sexual development

To probe the ability of ectopic Gls to activate the sexual program, we generated transgenic animals that expressed *Gls* ectopically in several contexts. Given the absence of conditional expression protocols for *Hydractinia*, we used the promoters of several genes to drive *Gls* in embryos. First, we used the genomic control elements of the *Rfamide* gene to drive *Gls* expression in embryos. *Rfamide* is expressed in a subset of neurons in the heads of feeding and sexual polyps (*13*). The transgenic construct also included GFP, separated by a T2A peptide sequence to mark transgenic cells in the mosaic animal. Most transgenic animals died during embryogenesis or before completing metamorphosis, and the remaining ones developed with severe patterning defects (fig. S6). We repeated the experiment using different genetic elements to drive *Gls* in *Tfap2* reporter animals: *Wnt3* is expressed in the oral tips of polyps (fig. S6); *Ncol1*, a nematogenesis marker, is expressed by differentiating nematocytes at the lower part of the body column (fig. S6). Last, we used the *B-tubulin* genomic control elements to drive *Gls* ubiquitously (fig. S6). All these constructs generated various, mild to severe defects in development, but no germ cells were induced as evidenced by lack of GFP reporter fluorescence (fig. S6). Only in rare cases did we observe structures that resembled sexual polyps in metamorphosed animals (fig. S6). Therefore, Gls is insufficient to induce sexual development precociously, or the transgenes did not produce mature Gls protein; the latter could not be verified.

## DISCUSSION

*Gls*, a member of the TGF-β family, is expressed by germ cells and required for patterning and maintenance of the somatic gonadal tissue. The gene is also present in some other hydrozoans (such as *Hydractinia*’s close relative, *Podocoryna carnea*) but missing in *Hydra* and *Nematostella* (fig. S2). We also demonstrate that the germinal zone within the gonad, in turn, facilitates the induction of new germ cells through activation of *Tfap2* in pluripotent i-cells.

We propose a model for sexual development in *Hydractinia*. In the model, *Tfap2* is induced in a small number of i-cells by an unknown factor, thereby committing them to germ cell fate. We call these primary germ cells. Primary germ cells secrete Gls protein that induces the development of the germinal zone that acts as a sexual organizer to drive the patterning of the entire gonad. Furthermore, the germinal zone secretes an unknown factor that induces *Tfap2* expression in resident pluripotent i-cells, committing them to germ cell fate that we call secondary germ cells. The gonadal somatic tissue also provides the microenvironment that promotes proliferation of germ cells and eventually their maturation to gametes. Secondary germ cells, in turn, secrete Gls to maintain the germinal zone and by proxy, the entire gonad. Therefore, a positive feedback loop induces and maintains sexual reproduction (Fig. 5C).

Several questions remain unanswered. First, through which receptor and effector does Gls signal? Both receptor and effector phylogenies (figs. S7 and S8) did not provide a conclusive answer. Moreover, mRNA expression plots of receptors and effectors in the *Hydractinia* cell atlas (*19*) revealed a broad distribution for all, with none being sexual polyp specific (fig. S9). Last, identifying signaling activity through phosphorylated Smad protein immunofluorescence is currently unfeasible because of lack of a specific antibody (*20*). Second, what is the nature of the signal that induces *Tfap2* in i-cells that become primary germ cells? Third, what is the signal that is emitted from the germinal zone in the somatic gonad to induce *Tfap2* expression in pluripotent i-cells and thereby convert them to secondary germ cells? The possibility that Gls itself acts on i-cells is unlikely because precocious expression of Gls was insufficient to induce germ cells. Moreover, if this were the case, germ cells would induce all i-cells in their vicinity to become germ cells too, and germ cell identity would spread uncontrollably. Therefore, Gls induces somatic gonad patterning, but the signal emitted by the gonad to induce secondary germ cells must be distinct from Gls to restrict secondary germ cell commitment to the germinal zone. Last, what molecular signals act downstream of Gls and induce proliferation of germ cells and their maturation to gametes?

It is interesting to speculate about the evolution of the germ cell–gonad interactions. Germ cells in all studied animals are specified before the development of gonads. Therefore, it is possible that induction of gonad morphogenesis by a TFG-β signal of germ cell origin is a primitive trait in animals. By contrast, because most bilaterians segregate a germ line only once in a lifetime, induction of secondary germ cells in the gonad does not exist in these animals. It either has been lost in bilaterians or constitutes a cnidarian innovation.

## MATERIALS AND METHODS

### Animals

*H. symbiolongicarpus* colonies were kept in artificial seawater (ASW) as described (*9*). Fertilized eggs were collected 1.5 hours after exposing male and female colonies to light.

### Grafting

*H. symbiolongicarpus* colonies were starved for a day and anesthetized in 4% MgCl_2_ (in 50% distilled water/50% filtered seawater). Polyps were dissected from the colony. Two feeding polyps were grafted at their wound side by skewering them onto a minutien pin (Fine Science Tools, catalog no. 26002-10). They were then pressed together by placing a block of 1% agarose gel at each end and left for 2 hours for the tissues to fuse. Grafts were removed from the minutien pin by gently pushing them toward the end of the pin with tweezers. Grafts were transferred to a glass petri dish with freshly 0.2-μm–filtered seawater and incubated overnight. The next day, one head of the grafts was removed. Donor tissue feeding heads or sexual polyp parts (oral tip, germinal zone, and mature sporosac) were isolated using a scalpel. Donor and recipient tissues were skewered onto a minutien pin. They were then pressed together by placing a piece of 1% agarose gel at each end and left for 4 hours for the wound to heal. Chimeras were removed from the minutien pin and transferred to a glass petri dish with freshly 0.2-μm–filtered seawater and incubated with agitation for 8 days at 18°C. Chimeras were fed with *Artemia franciscana* nauplii, and seawater was replaced daily.

### BrdU incubation

Sexual polyps were incubated for 1 hour in 150 μM BrdU (Sigma-Aldrich; catalog no. B5002) in ASW and fixed in 4% paraformaldedhyde/ASW.

### Injection of embryos

One-cell stage embryos of wild-type or *Tfap2* reporter animal were injected using a Narishige IM 300 microinjection system as described (*10*).

### Ectopic expression of *Gls*

*Gls* coding sequence in-frame with T2A peptide was synthetized by IDT. Insert was ligated into the 5’Wnt3::GFP::3’Wnt3 (*10*) and 5’RFamide::GFP::3’βtubulin (*13*) using restriction enzymes Not 1 and Sac 1. Insert was ligated into the 5’βtubulin::mScarlet::3’βtubulin (*10*) and 5’Ncol1::mScarlet::3’Ncol1 (*21*) plasmids using Gibson assembly according to the manufacturer recommendations (NEB, catalog no. E2611). Sequences of *Gibson* primers used were as follows: βtubulin-Fwd: CCAGGTCCAATGGTATCTAAAGGTGAAGC; βtubutulin-Rev:ACGATGACATTTTTTCAGATCCACCTCC; βtubutulin-Gls-T2A-Fwd: ATCTGAAAAAATGTCATCGTTGTTAATATTTTTG; βtubutulin-Gls-T2A-Rev: TAGATACCATTGGACCTGGGTTTTCTTC; *Ncol1-* Fwd: CCCAGGTCCAATGGTATCTAAAGGTGAAGC; Ncol1-Rev: ACGATGACATTACTGTAGGATTGTTATAATCG; Ncol1-Gls-T2A-Fwd: TCCTACAGTAATGTCATCGTTGTTAATATTTTTG; Ncol1-Gls-T2A-Rev: TAGATACCATTGGACCTGGGTTTTCTTC. The full sequence of the plasmids injected can be found in data S1.

Plasmids listed were injected at 500 to 1800 ng/μl and were supplemented with 200 mM KCl. The *Ncol1::Gls::mScarlet* and *B-Tubulin::Gls::mScarlet* were injected into *Tfap2::GFP* reporter embryos. The phenotype of the animal was assessed at 2 days after metamorphosis.

### CRISPR-Cas9 knockout

Single guide RNAs (sgRNAs) were designed using Geneious (2017.9.1.8). The three selected sgRNAs for *Gls* did not match other genomic sequence. Synthetic sgRNAs were provided by Synthego Inc. and diluted according to the manufacturer’s recommendations. All sgRNAs were incubated together (500 ng/μl) with recombinant Cas9 (1 μg/μl; IDT, catalog no. 1074181) for 15 min before injection as described in (*16*).

### Genotyping

Forty-five CRISPR-Cas9–injected animals were metamorphosed. Three animals displayed defects in sexual development. Animals were genotyped for *Gls* mutations. Primers spanning the entire second exon of the gene (see fig. S4 and data S1) were used in a PCR to identify large deletions. PCR products were sequenced, allowing us to identify a 601-bp deletion in Mutant 28. Sequence of the *Gls* mutant 28 used for the generation of G_1_ heterozygotes can be find in data S1. G_1_ animals were metamorphosed and grown to sexual maturity. G_1_ males and females that carried the 601-bp deletion were then crossed to obtained G_2_ offspring. G_2_ animals were genotyped, and eight homozygote mutant animals were identified.

DNA was extracted as described in (*16*). A two-step PCR approach was used to amplify genomic fragments. PCR conditions using Phusion High-Fidelity DNA Polymerase (Thermo Fisher Scientific, catalog no. F530S,) were as follows: initial denaturation (98°C, 3:00), denaturation (98°C, 0:30), annealing/extension (64°C, 2:30), repeat steps 2 to 3 (30×), and a final extension (64°C, 15:00). PCRs were run on a 1% agarose gel, and positive bands were excised and purified using Monarch Gel extraction clean kit (NEB, catalog no. T1020L). A-tailing of the fragments was carried out by incubating the DNA with MyTaq polymerase (NEB, catalog no. M0273) for 15 min. Inserts were then ligated into the pGEM-T Easy Vector System (Promega, catalog no. A1360). DH5-α *Escherichia coli* bacteria were transformed and plated on ampicillin LB agar plates and grown overnight. Individual clones were picked and grown in overnight cultures of LB broth (with ampicillin, 100 μg/ml). Plasmid DNA was extracted using the Monarch Miniprep Kit (NEB, catalog no. T1010L) and sent to sequencing to identify mutations.

### In situ hybridization

Probes were synthesized according to (*15*) using hairpins 27 and 30. Tissue was fixed and dehydrated as previously described in (*10*). In situ hybridization experiments were performed as described previously (*16*), with changes listed below. After rehydration, samples were incubated for 10 min in proteinase K (2 mg/ml) in phosphate-buffered saline (PBS) with 0.1% Tween 20 and fixed in 4% paraformaldedhyde/PBS.

### Immunofluorescence

Immunofluorescence staining was performed as previously described (*22*). Anti-Piwi1 antibodies were used at the concentration of 1:2000 (*10*); anti-GFP 1:300 (Synaptic Systems, catalog no. 132005); 1:100 anti-BrdU (Abcam, catalog no. ab6703).

### Phylogeny

Putative *Hydractinia* genes were identified with tBLASTn and BLASTp searches using human sequences as queries. Reciprocal BLAST was performed, including previously unidentified *Hydractinia* sequences. Previously unidentified genes from *Hydractinia* were next used as queries as well. After identification of genes in *Hydractinia*, proteins were analyzed with ScanProsite (ExPaSy) (*23*) and Pfam to verify the presence of TGF-β–specific domains.

For the TGF-β family, the dataset was built by collecting all sequences from a previous phylogenetic analysis of TGF family (*24*) to which we added *Hydractinia* sequences. In addition, we completed the dataset by incorporating additional sequences from selected species, especially the cnidarians *Hydra vulgaris*, *Nematostella vectensis*, and *P. carnea* (data S1), the lophotrochozoans *Biomphalaria glabrata*, cephalochordate *Branchiostoma floridae*, and teleost *Danio rerio* to build an alignment more representative of metazoan diversity. From these species, we added Nodal and TGF-β genes that were absent/underrepresented in the original alignment despite belonging to the TGF-β family (*24*).

For the Smad and TGF-β receptor dataset, we incorporated sequences from a previous study (*25*) in addition to our BLAST results. Multiple sequence alignment of amino acids was generated using MAFFT version 7 (*26*) with default parameters. Gblocks version 0.91b (*27*) was used to remove vacancies and blur sites. Final alignment is composed of 376 amino acids. Multiple sequence alignment can be found in data S1.

Phylogenetic analyses for the TGF-β gene family were conducted using IQ-TREE with automatic model selection (WAG+F+I+R5), 1000 bootstraps, and 1000 SH-aLRT. For the TGF-β receptors and Smad alignments, PhyML 3.1 software (*28*) was used. Blossum and JTT evolution models were used, respectively, determined to be most likely by MEGA11 (*29*). Node robustness was evaluated by conducting 500-bootstrap replicate sampling.

Bayesian analyses were performed using MrBayes (v3.2.6) (*30*) under a mixed model. Similarly, the WAG evolution model was determined to by the most likely under the Bayesian method. The phylogenetic analysis was carried out for 300,000 generations with 10 randomly started simultaneous Markov chains (1 cold chain, 9 heated chains) and sampled every 100 generations. One fourth of the topologies were discarded as burn-in values, while the remaining ones were used to calculate posterior probability. Node robustness corresponds to posterior probabilities. For both phylogenetic analyses, the outgroup is the human glial cell line–derived neurotrophic factor sequence (NCBI P39905.1) already successfully used to conduct TGF-β family phylogeny (*24*).

### Single-cell analysis

The expression of the identified genes was plotted in the *Hydractinia* cell type atlas dataset (*19*), both as Uniform Manifold Approximation and Projection (UMAP) and bubble expression plots. The code for plotting can be found at https://github.com/HorkanHR/CurantzGls2024, which also includes expression broken down by colony part.

### Imaging

Images of whole colonies and chimeras were taken using a Leica M165FC microscope with a Leica DFC7000T camera. Confocal images were acquired using an Olympus Fluoview 3000 laser scanning confocal microscope. Images were imported into ImageJ/Fiji. Their brightness and contrast were adjusted as a whole. To facilitate visualization, a Gaussian blur correction was applied to the images of the SABER in situ. We aimed to display all polyp images in the same orientation; therefore, some images were rotated, and a black rectangle was inserted behind for aesthetic reasons.
