# Supplementary material for: A positive feedback loop between germ cells and gonads induces and maintains sexual reproduction in a cnidarian
Source: Sci Adv. 2025 Jan 8;11(2):eadq8220. doi: 10.1126/sciadv.adq8220 (PMC11708894; doi:10.1126/sciadv.adq8220)
Supplement: Supplementary file 1 — Figs. S1 to S9 Tables S1 and S2 Data S1 [file sciadv.adq8220_sm.pdf]

Science Advances
AAAS

Supplementary Materials for

A positive feedback loop between germ cells and gonads induces and maintains sexual reproduction in a cnidarian

Camille Curantz *et al.*

Corresponding author: Uri Frank, uri.frank@universityofgalway.ie

Sci. Adv. **11**, eadq8220 (2025)
DOI: 10.1126/sciadv.adq8220

This PDF file includes:

Figs. S1 to S9
Tables S1 and S2
Data S1

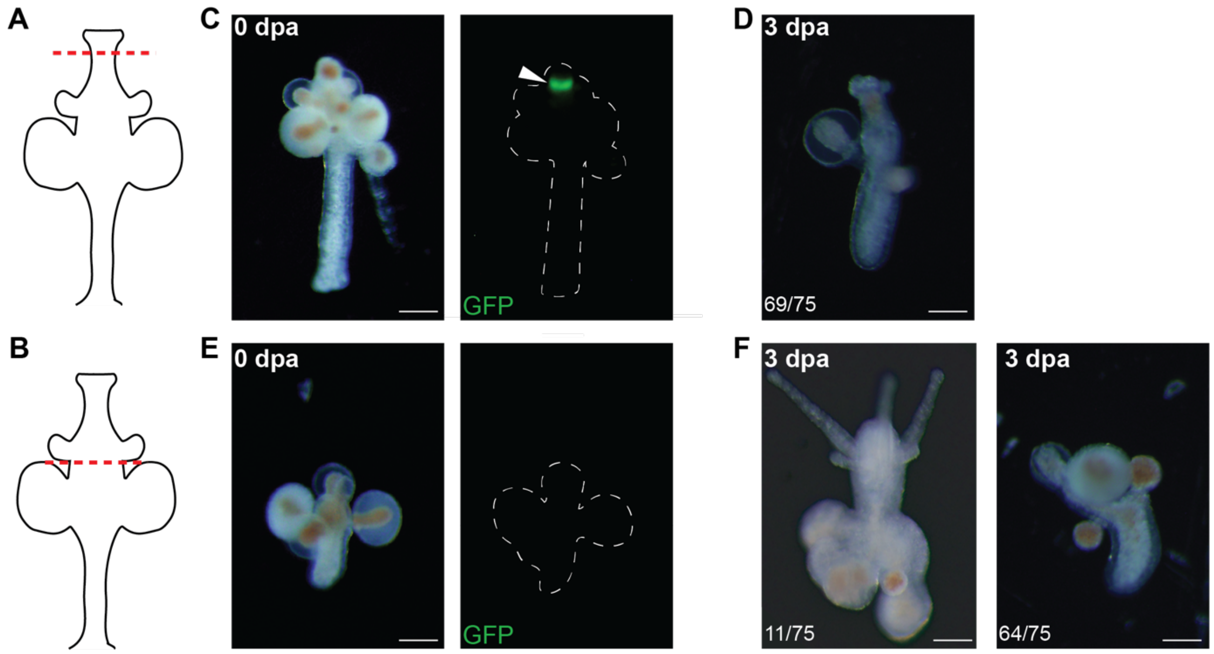

Fig. S1. Sexual head regeneration**.**

(**A, B**) Cartoon of sexual polyps. Red dashed lines indicate amputation planes for the experiments shown in C and D, and E and F, respectively. Pictures of regenerating *Tfap2*::GFP polyp at 0 and 3 days post amputation (dpa). White dashed lines show the outline of the polyp in the GFP channels. Scale bars 100 µm.

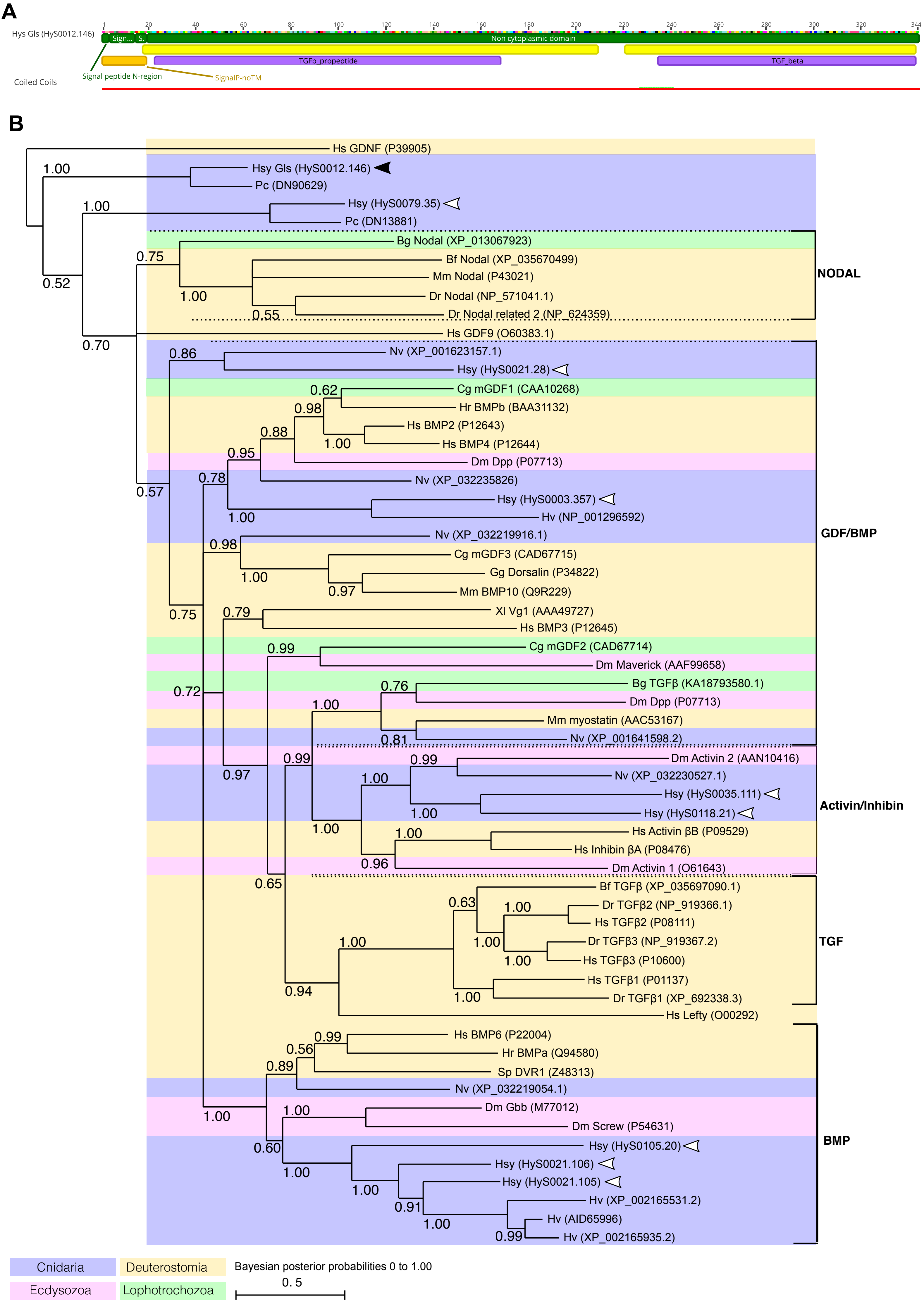
A
Hys Gls (HyS0012.146)
Sign...
S.
Non cytoplasmic domain
TGFb_propeptide
TGF_beta
Coiled Coils
Signal peptide N-region
SignalP-noTM
B
Hs GDNF (P39905)
Hsy Gls (HyS0012.146)
Pc (DN90629)
Hsy (HyS0079.35)
Pc (DN13881)
Bg Nodal (XP_013067923)
Bf Nodal (XP_035670499)
Mm Nodal (P43021)
Dr Nodal (NP_571041.1)
Dr Nodal related 2 (NP_624359)
NODAL
Hs GDF9 (O60383.1)
Nv (XP_001623157.1)
Hsy (HyS0021.28)
Cg mGDF1 (CAA10268)
Hr BMPb (BAA31132)
Hs BMP2 (P12643)
Hs BMP4 (P12644)
Dm Dpp (P07713)
Nv (XP_032235826)
Hsy (HyS0003.357)
Hv (NP_001296592)
Nv (XP_032219916.1)
Cg mGDF3 (CAD67715)
Gg Dorsalin (P34822)
Mm BMP10 (Q9R229)
Xl Vg1 (AAA49727)
Hs BMP3 (P12645)
Cg mGDF2 (CAD67714)
Dm Maverick (AAF99658)
Bg TGFβ (KA18793580.1)
Dm Dpp (P07713)
Mm myostatin (AAC53167)
Nv (XP_001641598.2)
GDF/BMP
Dm Activin 2 (AAN10416)
Nv (XP_032230527.1)
Hsy (HyS0035.111)
Hsy (HyS0118.21)
Hs Activin βB (P09529)
Hs Inhibin βA (P08476)
Dm Activin 1 (O61643)
Activin/Inhibin
Bf TGFβ (XP_035697090.1)
Dr TGFβ2 (NP_919366.1)
Hs TGFβ2 (P08111)
Dr TGFβ3 (NP_919367.2)
Hs TGFβ3 (P10600)
Hs TGFβ1 (P01137)
Dr TGFβ1 (XP_692338.3)
Hs Lefty (O00292)
TGF
Hs BMP6 (P22004)
Hr BMPa (Q94580)
Sp DVR1 (Z48313)
Nv (XP_032219054.1)
Dm Gbb (M77012)
Dm Screw (P54631)
Hsy (HyS0105.20)
Hsy (HyS0021.106)
Hsy (HyS0021.105)
Hv (XP_002165531.2)
Hv (AID65996)
Hv (XP_002165935.2)
BMP
Cnidaria
Deuterostomia
Ecdysozoa
Lophotrochozoa
Bayesian posterior probabilities 0 to 1.00
0. 5

C

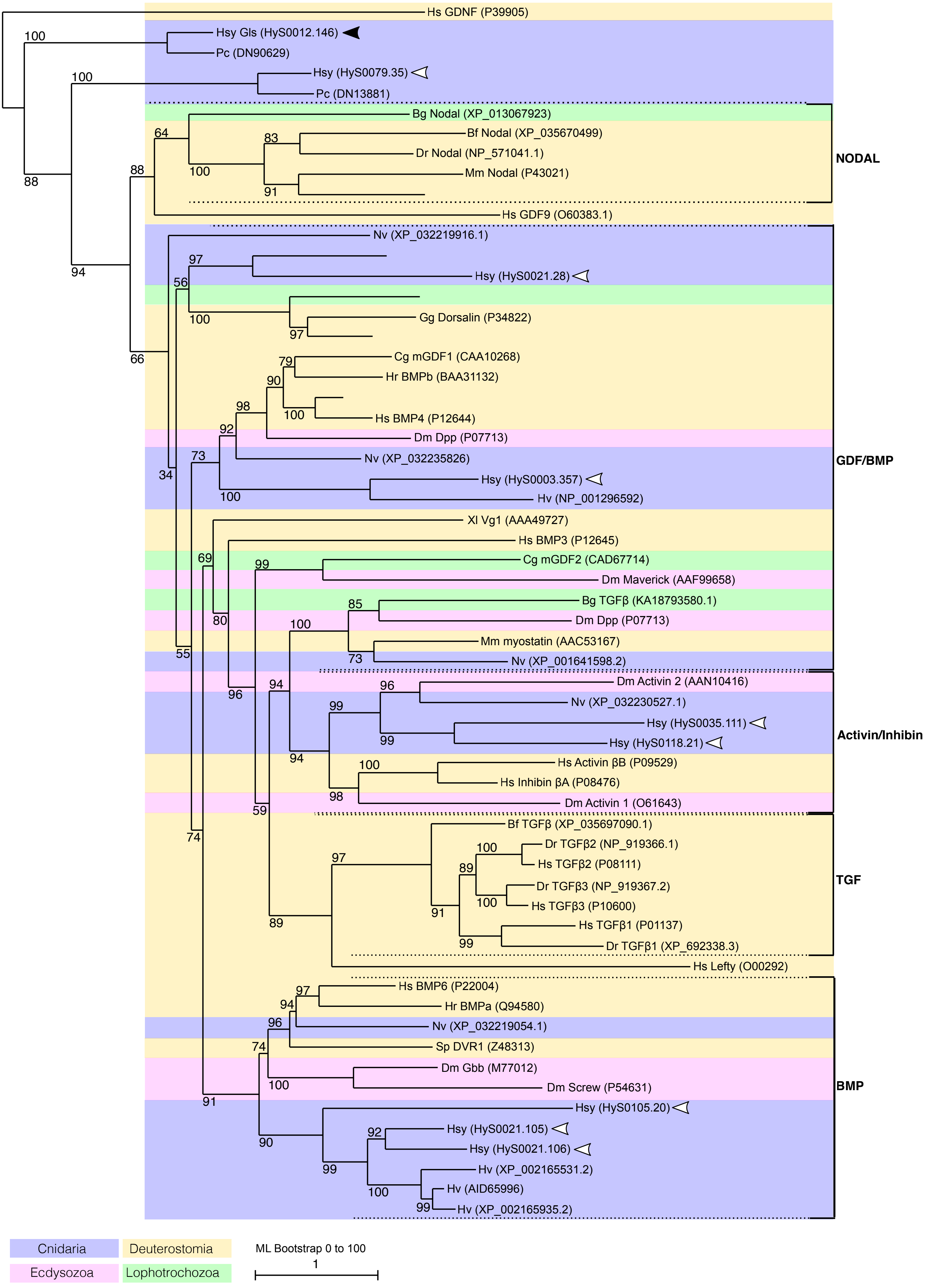

Hs GDNF (P39905)
Hsy Gls (HyS0012.146)
Pc (DN90629)
Hsy (HyS0079.35)
Pc (DN13881)
Bg Nodal (XP_013067923)
Bf Nodal (XP_035670499)
Dr Nodal (NP_571041.1)
Mm Nodal (P43021)
Hs GDF9 (O60383.1)
NODAL
Nv (XP_032219916.1)
Hsy (HyS0021.28)
Gg Dorsalin (P34822)
Cg mGDF1 (CAA10268)
Hr BMPb (BAA31132)
Hs BMP4 (P12644)
Dm Dpp (P07713)
Nv (XP_032235826)
Hsy (HyS0003.357)
Hv (NP_001296592)
GDF/BMP
Xl Vg1 (AAA49727)
Hs BMP3 (P12645)
Cg mGDF2 (CAD67714)
Dm Maverick (AAF99658)
Bg TGFβ (KA18793580.1)
Dm Dpp (P07713)
Mm myostatin (AAC53167)
Nv (XP_001641598.2)
Dm Activin 2 (AAN10416)
Nv (XP_032230527.1)
Hsy (HyS0035.111)
Hsy (HyS0118.21)
Activin/Inhibin
Hs Activin βB (P09529)
Hs Inhibin βA (P08476)
Dm Activin 1 (O61643)
Bf TGFβ (XP_035697090.1)
Dr TGFβ2 (NP_919366.1)
Hs TGFβ2 (P08111)
Dr TGFβ3 (NP_919367.2)
Hs TGFβ3 (P10600)
Hs TGFβ1 (P01137)
Dr TGFβ1 (XP_692338.3)
TGF
Hs Lefty (O00292)
Hs BMP6 (P22004)
Hr BMPa (Q94580)
Nv (XP_032219054.1)
Sp DVR1 (Z48313)
Dm Gbb (M77012)
Dm Screw (P54631)
BMP
Hsy (HyS0105.20)
Hsy (HyS0021.105)
Hsy (HyS0021.106)
Hv (XP_002165531.2)
Hv (AID65996)
Hv (XP_002165935.2)
Cnidaria
Deuterostomia
Ecdysozoa
Lophotrochozoa
ML Bootstrap 0 to 100
1

Fig. S2. Gls phylogeny

(A) Domain analysis of Gls showing a typical TGF-β-like structure. Topologies of TGF-β multigene family obtained by phylogenetic analysis using Bayesian inference (**B**) and maximum likelihood (**C**) methods on amino acid sequences. The phylogenetic trees are rooted by the human protein GDNF (Glial Derived Neurotrophic Factor). The two analyses gave rise to similar topologies with some variation, but the deep nodes remain conserved. In both, Gls branches close to the root with its *Podocoryna* homologue. The Nodal clade branches next. The rest of the tree is subdivided between BMPs, TGF-βs, Activins/Inhibins, and a group composed of both GDFs and BMPs. The *Hydractinia* genome encodes nine TGF-β family members, including two GDFs/BMPs, two Activins/Inhibins, three BMP's, and two genes that branch close to the root of the tree, including Gonadless. *Hydractinia* sequences are labelled with white arrows, except for Gonadless which is labelled with a black arrow. (Bf: *Branchiostoma floridae*; Bg: *Biomphalaria glabrata*; Dr: *Danio rerio* Dm: *Drosophila melanogaster*; Ce: *Caenorhabditis elegans*; Cg: *Crassostrea gigas*; Hr: *Halocynthia roretzi*; Hs: *Homo sapiens*; Hv: *Hydra vulgaris*; Hsy: *Hydractinia symbiolongicarpus*; Mm: *Mus musculus*; Nv: *Nematostella vectensis*; Pc: *Podocoryna carnea;* Sp: *Strongylocentrotus purpuratus*; Xl: *Xenopus laevis*). Node robustness are posterior probabilities and bootstraps (1000 replicates) for Bayesian and maximum likelihood analyses, respectively.

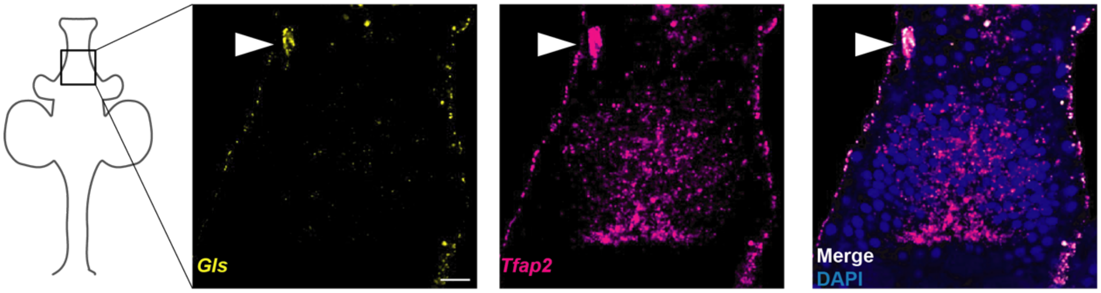

Fig. S3. Expression of *Gls* and *Tfap2*

Single confocal section of *in situ* mRNAs localization of *Tfap2* and *Gls* in the gastrodermis of a sexual polyp. Partial overlap in expression is shown. Scale bar 10 µm. Arrowheads point to a cell expressing both genes.

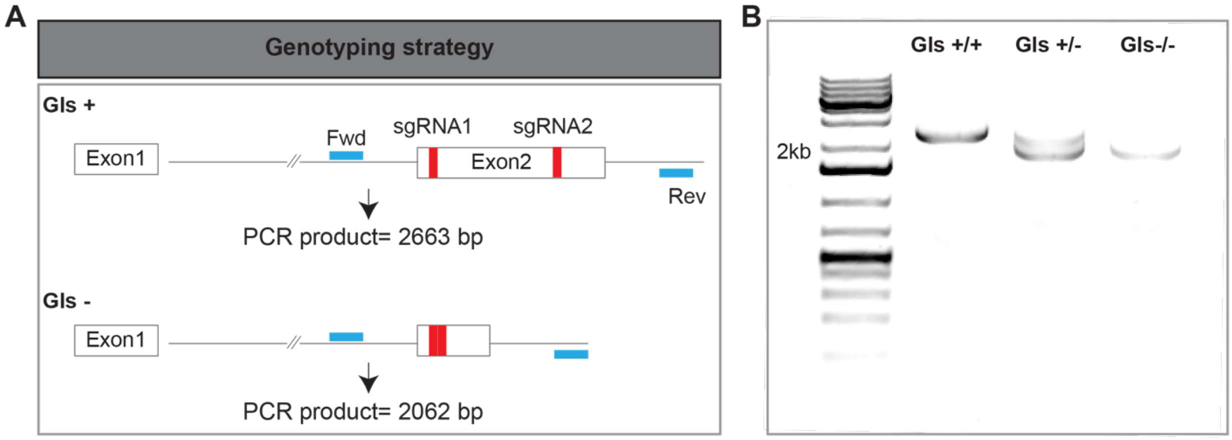

Fig. S4. Screening for *Gls* mutants.

(**A**) Graphical representation of the wild type and mutated alleles and the screening strategy. Red bars show the position of the Cas9 cut sites. (**B**) Results of the PCR genotyping showing wild type, heterozygous mutant, and homozygous mutant.

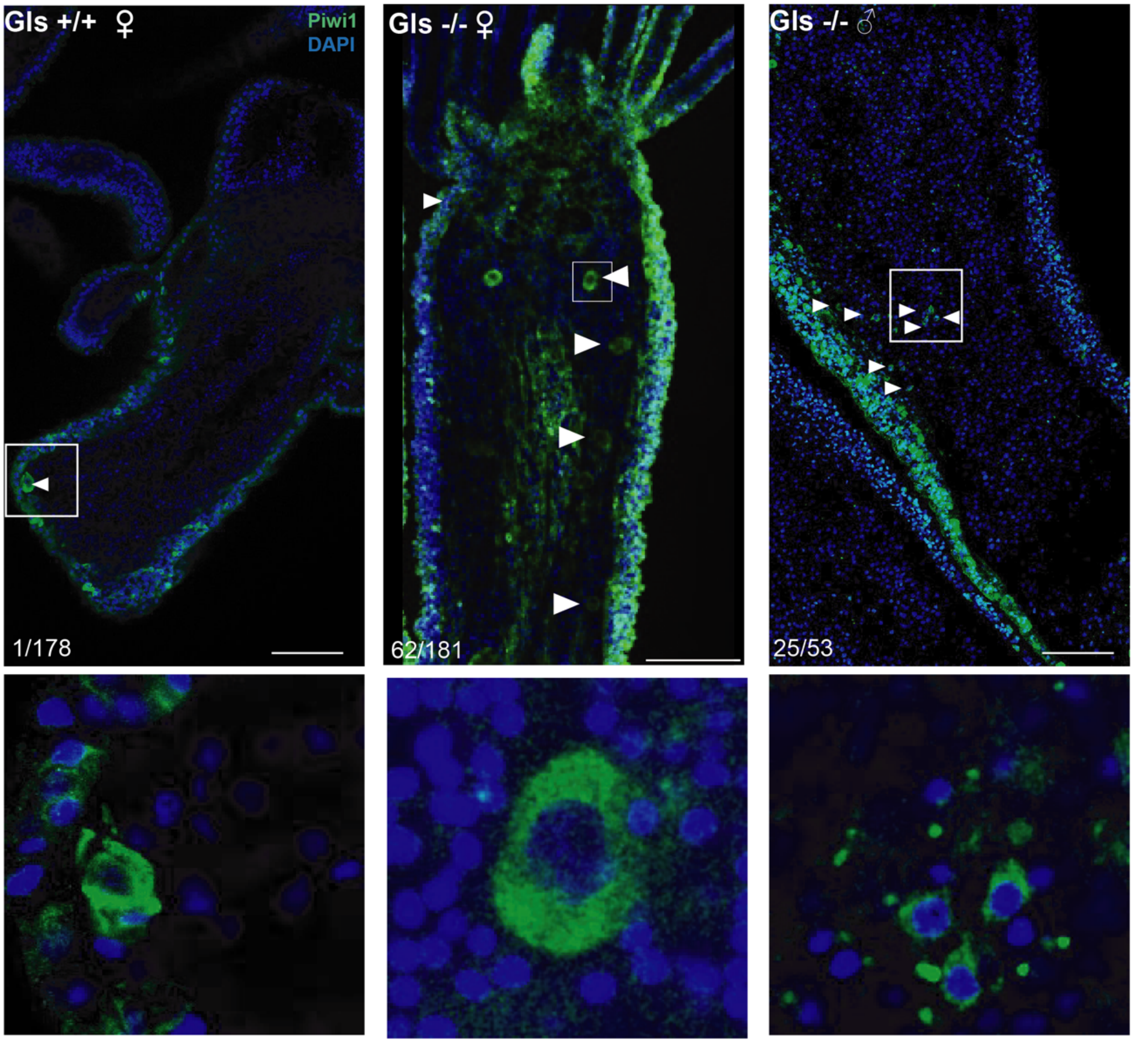

Fig. S5. Piwi1$^+$ cells in wild type and Gls$^{-/-}$.

Confocal images of a female Gls$^{+/+}$ and a male Gls$^{-/-}$ showing Piwi1$^+$ germ cells (white arrows) in their gastrodermis. White boxes represent a 50 μm close-up of these cells. Scale bar 50 μm.

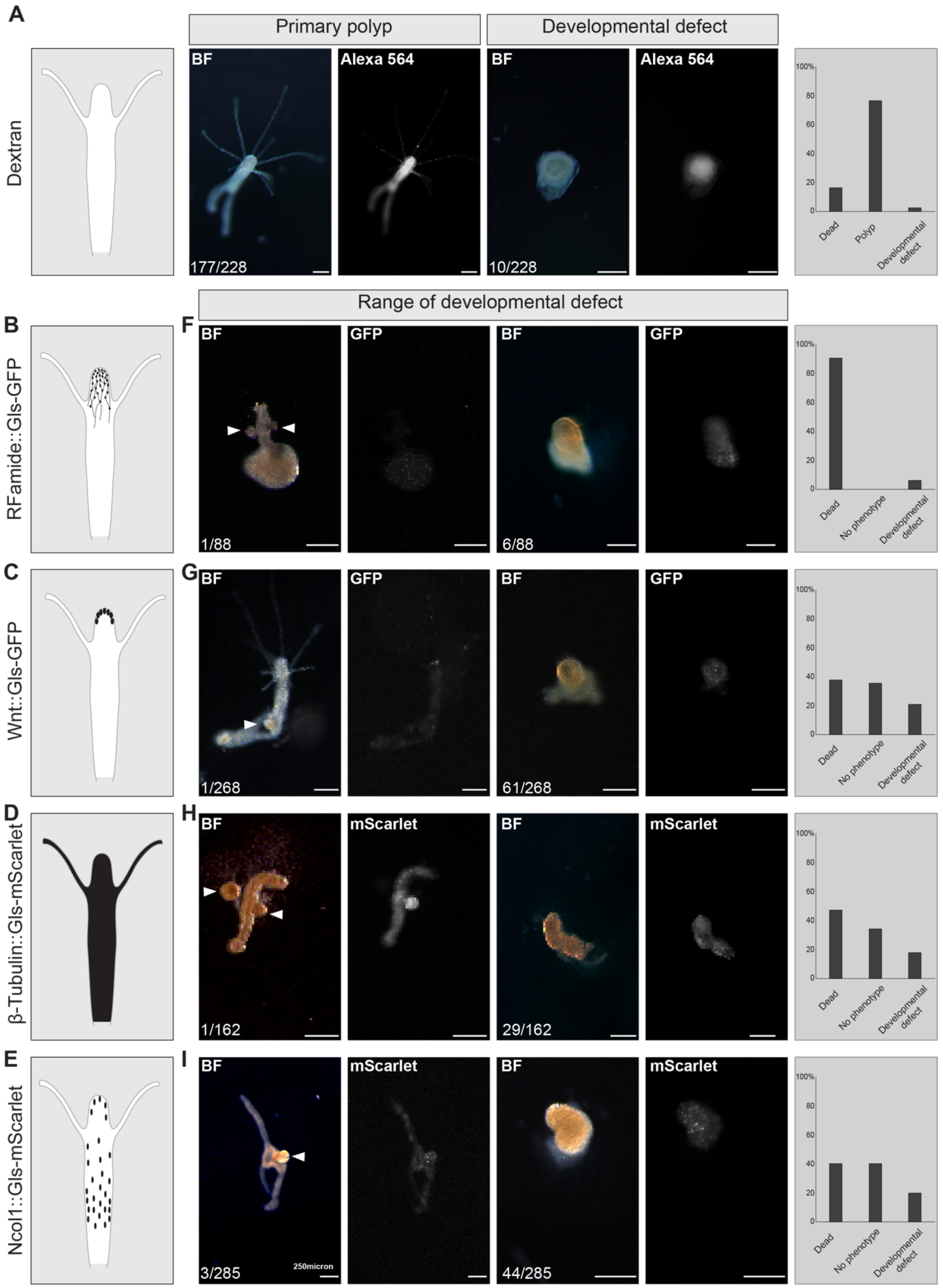
A
Primary polyp
Developmental defect
Dextran
BF
Alexa 564
177/228
10/228
100%
80
60
40
20
0
Dead
Polyp
Developmental defect
Range of developmental defect
B
RFamide::GIs-GFP
F
GFP
1/88
6/88
No phenotype
C
Wnt::GIs-GFP
G
1/268
61/268
D
β-Tubulin::GIs-mScarlet
H
mScarlet
1/162
29/162
E
Ncol1::GIs-mScarlet
I
3/285
250micron
44/285

Fig. S6. Ectopic expression of Gls in embryos.

(**A**) Control embryos injected with dextran 565 develop into a primary feeding polyp. (**B**) *Rfamide* precursor promoter-driven *Gls::GFP*. (**C**) *Wnt3* promoter-driven *Gls::GFP* (**D**) *B-tubulin* promoter-driven *Gls::mScarlet* injected in *Tfap2*::*GFP* reporter animals (**E**) *Ncol1* promoter-driven *Gls::mScarlet* injected in *Tfap2*::*GFP* reporter animals. No germ cells (GFP^+ cells) were detected (**F-I**) Transgenes expression cause mild to severe developmental defects. White arrows indicated structures that resembled sexual tissue in metamorphosed animals. Scale bar 250 μm. BF: bright field

A

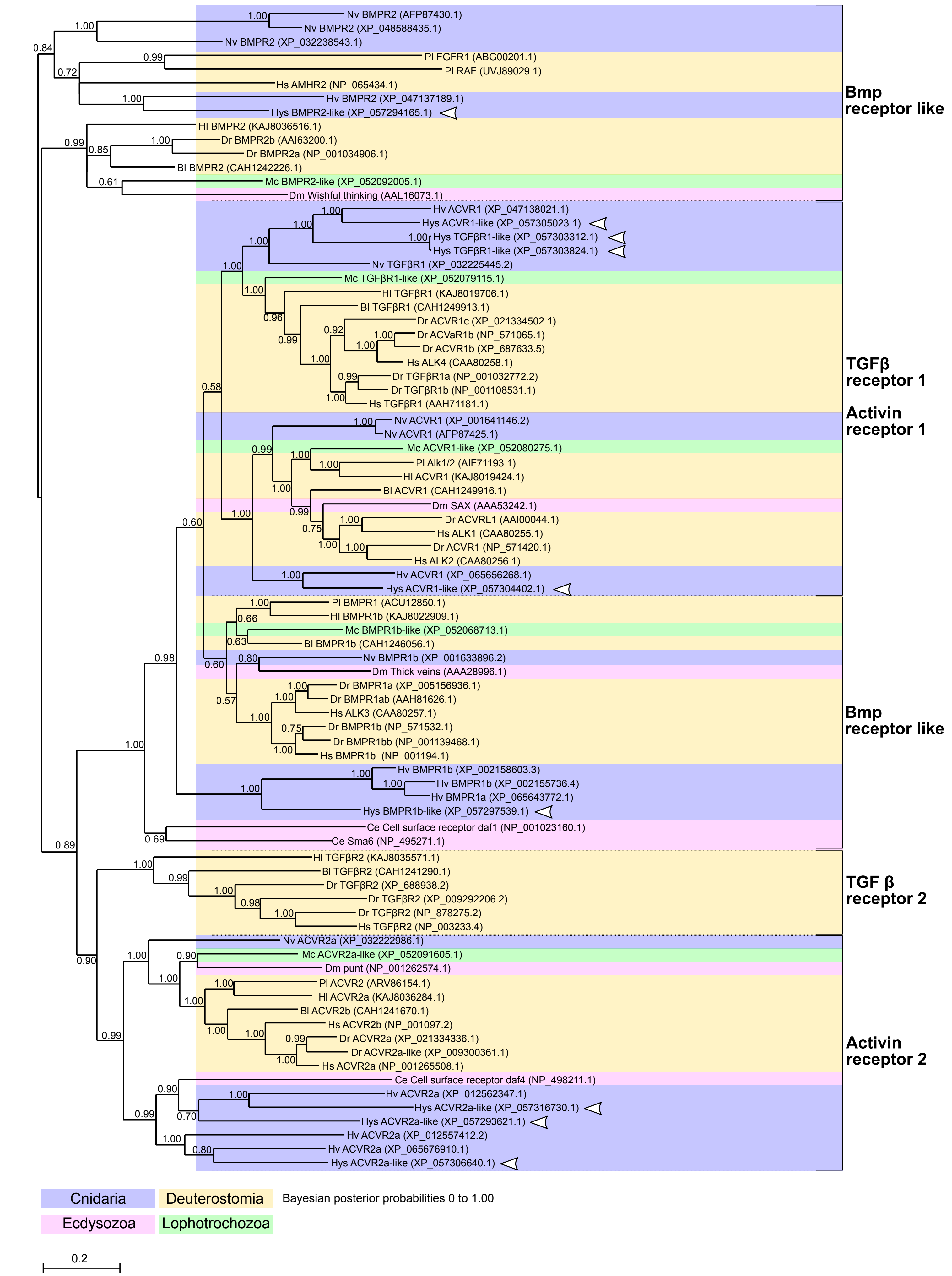
Nv BMPR2 (AFP87430.1)
Nv BMPR2 (XP_048588435.1)
Nv BMPR2 (XP_032238543.1)
Pl FGFR1 (ABG00201.1)
Pl RAF (UVJ89029.1)
Hs AMHR2 (NP_065434.1)
Hv BMPR2 (XP_047137189.1)
Hys BMPR2-like (XP_057294165.1)
Hl BMPR2 (KAJ8036516.1)
Dr BMPR2b (AAI63200.1)
Dr BMPR2a (NP_001034906.1)
Bl BMPR2 (CAH1242226.1)
Mc BMPR2-like (XP_052092005.1)
Dm Wishful thinking (AAL16073.1)
Bmp receptor like
Hv ACVR1 (XP_047138021.1)
Hys ACVR1-like (XP_057305023.1)
Hys TGFβR1-like (XP_057303312.1)
Hys TGFβR1-like (XP_057303824.1)
Nv TGFβR1 (XP_032225445.2)
Mc TGFβR1-like (XP_052079115.1)
Hl TGFβR1 (KAJ8019706.1)
Bl TGFβR1 (CAH1249913.1)
Dr ACVR1c (XP_021334502.1)
Dr ACVaR1b (NP_571065.1)
Dr ACVR1b (XP_687633.5)
Hs ALK4 (CAA80258.1)
Dr TGFβR1a (NP_001032772.2)
Dr TGFβR1b (NP_001108531.1)
Hs TGFβR1 (AAH71181.1)
TGFβ receptor 1
Activin receptor 1
Nv ACVR1 (XP_001641146.2)
Nv ACVR1 (AFP87425.1)
Mc ACVR1-like (XP_052080275.1)
Pl Alk1/2 (AIF71193.1)
Hl ACVR1 (KAJ8019424.1)
Bl ACVR1 (CAH1249916.1)
Dm SAX (AAA53242.1)
Dr ACVRL1 (AAI00044.1)
Hs ALK1 (CAA80255.1)
Dr ACVR1 (NP_571420.1)
Hs ALK2 (CAA80256.1)
Hv ACVR1 (XP_065656268.1)
Hys ACVR1-like (XP_057304402.1)
Pl BMPR1 (ACU12850.1)
Hl BMPR1b (KAJ8022909.1)
Mc BMPR1b-like (XP_052068713.1)
Bl BMPR1b (CAH1246056.1)
Nv BMPR1b (XP_001633896.2)
Dm Thick veins (AAA28996.1)
Dr BMPR1a (XP_005156936.1)
Dr BMPR1ab (AAH81626.1)
Hs ALK3 (CAA80257.1)
Dr BMPR1b (NP_571532.1)
Dr BMPR1bb (NP_001139468.1)
Hs BMPR1b (NP_001194.1)
Bmp receptor like
Hv BMPR1b (XP_002158603.3)
Hv BMPR1b (XP_002155736.4)
Hv BMPR1a (XP_065643772.1)
Hys BMPR1b-like (XP_057297539.1)
Ce Cell surface receptor daf1 (NP_001023160.1)
Ce Sma6 (NP_495271.1)
Hl TGFβR2 (KAJ8035571.1)
Bl TGFβR2 (CAH1241290.1)
Dr TGFβR2 (XP_688938.2)
Dr TGFβR2 (XP_009292206.2)
Dr TGFβR2 (NP_878275.2)
Hs TGFβR2 (NP_003233.4)
TGF β receptor 2
Nv ACVR2a (XP_032222986.1)
Mc ACVR2a-like (XP_052091605.1)
Dm punt (NP_001262574.1)
Pl ACVR2 (ARV86154.1)
Hl ACVR2a (KAJ8036284.1)
Bl ACVR2b (CAH1241670.1)
Hs ACVR2b (NP_001097.2)
Dr ACVR2a (XP_021334336.1)
Dr ACVR2a-like (XP_009300361.1)
Hs ACVR2a (NP_001265508.1)
Activin receptor 2
Ce Cell surface receptor daf4 (NP_498211.1)
Hv ACVR2a (XP_012562347.1)
Hys ACVR2a-like (XP_057316730.1)
Hys ACVR2a-like (XP_057293621.1)
Hv ACVR2a (XP_012557412.2)
Hv ACVR2a (XP_065676910.1)
Hys ACVR2a-like (XP_057306640.1)
Cnidaria
Deuterostomia
Ecdysozoa
Lophotrochozoa
Bayesian posterior probabilities 0 to 1.00
0.2

B

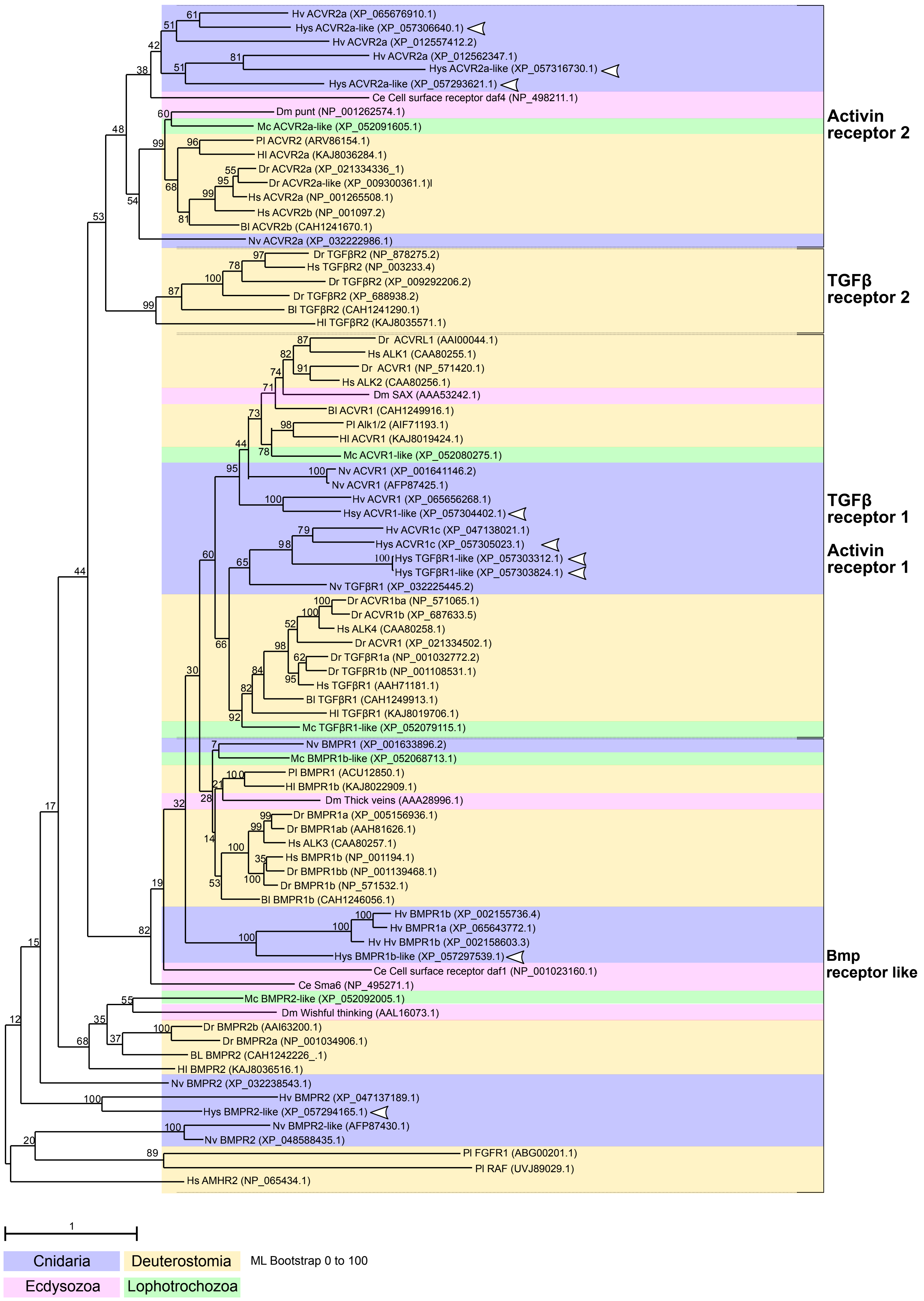

Hv ACVR2a (XP_065676910.1)
Hys ACVR2a-like (XP_057306640.1)
Hv ACVR2a (XP_012557412.2)
Hv ACVR2a (XP_012562347.1)
Hys ACVR2a-like (XP_057316730.1)
Hys ACVR2a-like (XP_057293621.1)
Ce Cell surface receptor daf4 (NP_498211.1)
Dm punt (NP_001262574.1)
Mc ACVR2a-like (XP_052091605.1)
Pl ACVR2 (ARV86154.1)
Hl ACVR2a (KAJ8036284.1)
Dr ACVR2a (XP_021334336_1)
Dr ACVR2a-like (XP_009300361.1)l
Hs ACVR2a (NP_001265508.1)
Hs ACVR2b (NP_001097.2)
Bl ACVR2b (CAH1241670.1)
Nv ACVR2a (XP_032222986.1)
Dr TGFβR2 (NP_878275.2)
Hs TGFβR2 (NP_003233.4)
Dr TGFβR2 (XP_009292206.2)
Dr TGFβR2 (XP_688938.2)
Bl TGFβR2 (CAH1241290.1)
Hl TGFβR2 (KAJ8035571.1)
Dr ACVRL1 (AAI00044.1)
Hs ALK1 (CAA80255.1)
Dr ACVR1 (NP_571420.1)
Hs ALK2 (CAA80256.1)
Dm SAX (AAA53242.1)
Bl ACVR1 (CAH1249916.1)
Pl Alk1/2 (AIF71193.1)
Hl ACVR1 (KAJ8019424.1)
Mc ACVR1-like (XP_052080275.1)
Nv ACVR1 (XP_001641146.2)
Nv ACVR1 (AFP87425.1)
Hv ACVR1 (XP_065656268.1)
Hsy ACVR1-like (XP_057304402.1)
Hv ACVR1c (XP_047138021.1)
Hys ACVR1c (XP_057305023.1)
Hys TGFβR1-like (XP_057303312.1)
Hys TGFβR1-like (XP_057303824.1)
Nv TGFβR1 (XP_032225445.2)
Dr ACVR1ba (NP_571065.1)
Dr ACVR1b (XP_687633.5)
Hs ALK4 (CAA80258.1)
Dr ACVR1 (XP_021334502.1)
Dr TGFβR1a (NP_001032772.2)
Dr TGFβR1b (NP_001108531.1)
Hs TGFβR1 (AAH71181.1)
Bl TGFβR1 (CAH1249913.1)
Hl TGFβR1 (KAJ8019706.1)
Mc TGFβR1-like (XP_052079115.1)
Nv BMPR1 (XP_001633896.2)
Mc BMPR1b-like (XP_052068713.1)
Pl BMPR1 (ACU12850.1)
Hl BMPR1b (KAJ8022909.1)
Dm Thick veins (AAA28996.1)
Dr BMPR1a (XP_005156936.1)
Dr BMPR1ab (AAH81626.1)
Hs ALK3 (CAA80257.1)
Hs BMPR1b (NP_001194.1)
Dr BMPR1bb (NP_001139468.1)
Dr BMPR1b (NP_571532.1)
Bl BMPR1b (CAH1246056.1)
Hv BMPR1b (XP_002155736.4)
Hv BMPR1a (XP_065643772.1)
Hv Hv BMPR1b (XP_002158603.3)
Hys BMPR1b-like (XP_057297539.1)
Ce Cell surface receptor daf1 (NP_001023160.1)
Ce Sma6 (NP_495271.1)
Mc BMPR2-like (XP_052092005.1)
Dm Wishful thinking (AAL16073.1)
Dr BMPR2b (AAI63200.1)
Dr BMPR2a (NP_001034906.1)
BL BMPR2 (CAH1242226_.1)
Hl BMPR2 (KAJ8036516.1)
Nv BMPR2 (XP_032238543.1)
Hv BMPR2 (XP_047137189.1)
Hys BMPR2-like (XP_057294165.1)
Nv BMPR2-like (AFP87430.1)
Nv BMPR2 (XP_048588435.1)
Pl FGFR1 (ABG00201.1)
Pl RAF (UVJ89029.1)
Hs AMHR2 (NP_065434.1)
Activin receptor 2
TGFβ receptor 2
TGFβ receptor 1
Activin receptor 1
Bmp receptor like
1
Cnidaria
Deuterostomia
Ecdysozoa
Lophotrochozoa
ML Bootstrap 0 to 100

Fig. S7. TGF-β receptors phylogeny

Unrooted topologies of TGF-β receptor multigene family obtained by phylogenetic analysis using Bayesian inference (**A**) and maximum likelihood (**B**) methods, on amino acid sequences. The two analyses gave rise to similar topologies with a paraphyletic distribution of BMP receptor-like and two well defined clusters which are the Activin receptor 2 and the TGFβ receptor 2 groups. A third cluster comprises a set of both TGFβ receptor and Activin receptor 1. The *Hydractinia* genome encodes nine TGFβ receptors-like genes, including three Activin receptor 2, four TGFβ receptors plus Activin receptor 1 group, while the last two are BMP receptor-like without clear affiliations. No TGFβ receptor 2 was detected. Node robustness are posterior probabilities and bootstraps (500 replicates) for Bayesian and maximum likelihood analyses, respectively.

A

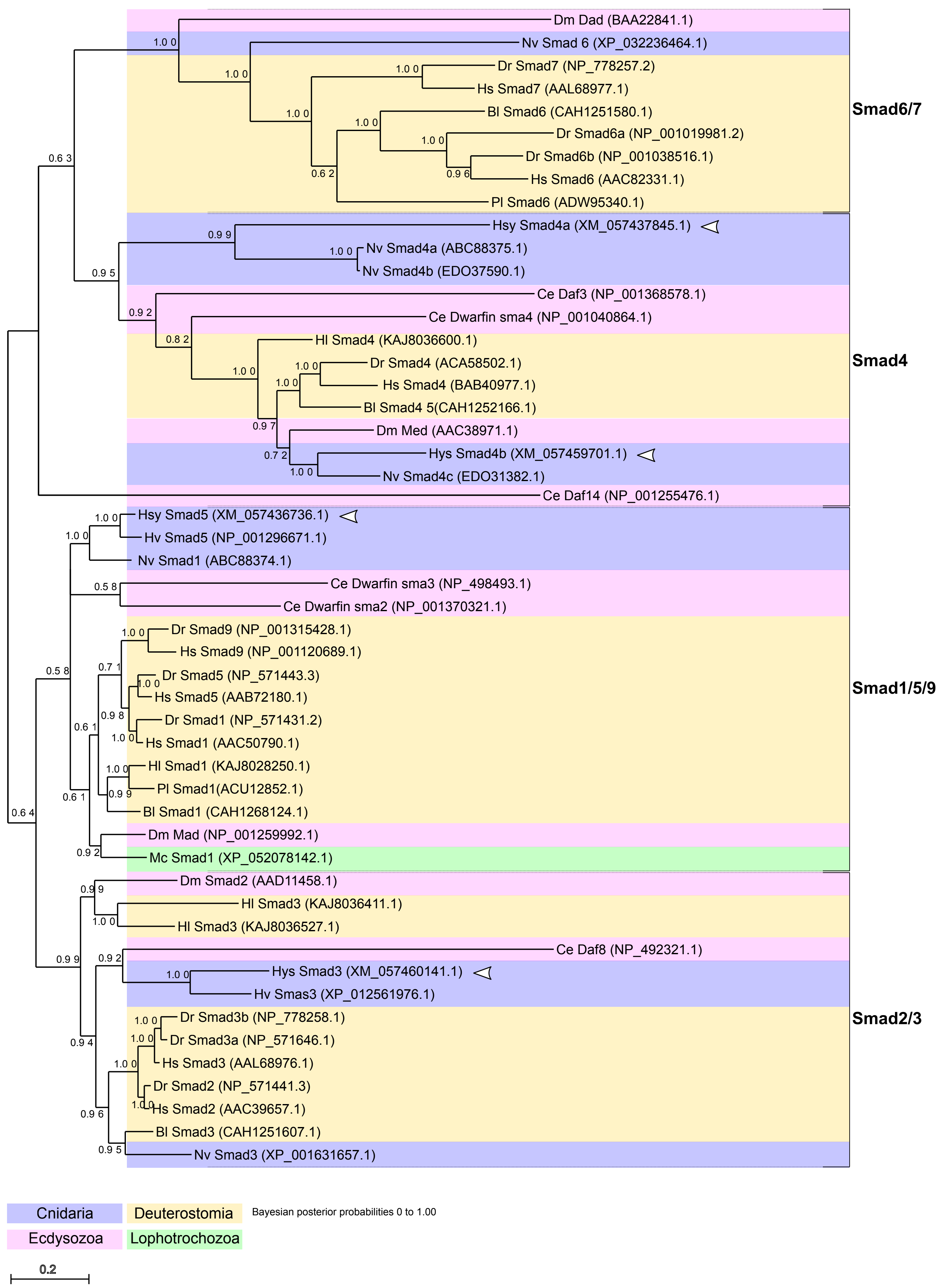
Dm Dad (BAA22841.1)
Nv Smad 6 (XP_032236464.1)
Dr Smad7 (NP_778257.2)
Hs Smad7 (AAL68977.1)
Bl Smad6 (CAH1251580.1)
Dr Smad6a (NP_001019981.2)
Dr Smad6b (NP_001038516.1)
Hs Smad6 (AAC82331.1)
Pl Smad6 (ADW95340.1)
Smad6/7
Hsy Smad4a (XM_057437845.1)
Nv Smad4a (ABC88375.1)
Nv Smad4b (EDO37590.1)
Ce Daf3 (NP_001368578.1)
Ce Dwarfin sma4 (NP_001040864.1)
Hl Smad4 (KAJ8036600.1)
Dr Smad4 (ACA58502.1)
Hs Smad4 (BAB40977.1)
Bl Smad4 5(CAH1252166.1)
Dm Med (AAC38971.1)
Hys Smad4b (XM_057459701.1)
Nv Smad4c (EDO31382.1)
Ce Daf14 (NP_001255476.1)
Smad4
Hsy Smad5 (XM_057436736.1)
Hv Smad5 (NP_001296671.1)
Nv Smad1 (ABC88374.1)
Ce Dwarfin sma3 (NP_498493.1)
Ce Dwarfin sma2 (NP_001370321.1)
Dr Smad9 (NP_001315428.1)
Hs Smad9 (NP_001120689.1)
Dr Smad5 (NP_571443.3)
Hs Smad5 (AAB72180.1)
Dr Smad1 (NP_571431.2)
Hs Smad1 (AAC50790.1)
Hl Smad1 (KAJ8028250.1)
Pl Smad1(ACU12852.1)
Bl Smad1 (CAH1268124.1)
Dm Mad (NP_001259992.1)
Mc Smad1 (XP_052078142.1)
Smad1/5/9
Dm Smad2 (AAD11458.1)
Hl Smad3 (KAJ8036411.1)
Hl Smad3 (KAJ8036527.1)
Ce Daf8 (NP_492321.1)
Hys Smad3 (XM_057460141.1)
Hv Smas3 (XP_012561976.1)
Dr Smad3b (NP_778258.1)
Dr Smad3a (NP_571646.1)
Hs Smad3 (AAL68976.1)
Dr Smad2 (NP_571441.3)
Hs Smad2 (AAC39657.1)
Bl Smad3 (CAH1251607.1)
Nv Smad3 (XP_001631657.1)
Smad2/3
Cnidaria
Deuterostomia
Ecdysozoa
Lophotrochozoa
Bayesian posterior probabilities 0 to 1.00
0.2

B

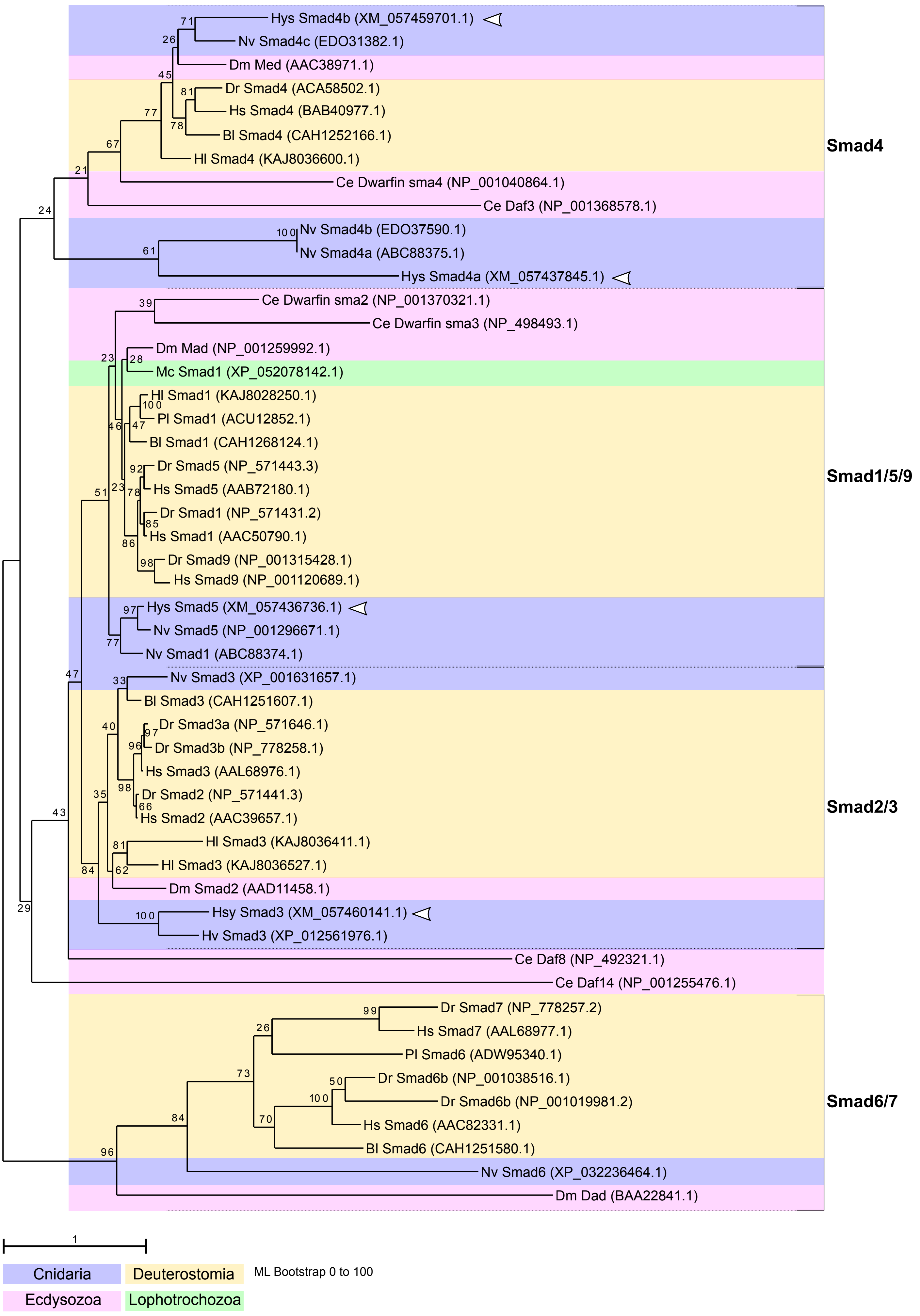

Hys Smad4b (XM_057459701.1)
Nv Smad4c (EDO31382.1)
Dm Med (AAC38971.1)
Dr Smad4 (ACA58502.1)
Hs Smad4 (BAB40977.1)
Bl Smad4 (CAH1252166.1)
Hl Smad4 (KAJ8036600.1)
Ce Dwarfin sma4 (NP_001040864.1)
Ce Daf3 (NP_001368578.1)
Nv Smad4b (EDO37590.1)
Nv Smad4a (ABC88375.1)
Hys Smad4a (XM_057437845.1)
Smad4
Ce Dwarfin sma2 (NP_001370321.1)
Ce Dwarfin sma3 (NP_498493.1)
Dm Mad (NP_001259992.1)
Mc Smad1 (XP_052078142.1)
Hl Smad1 (KAJ8028250.1)
Pl Smad1 (ACU12852.1)
Bl Smad1 (CAH1268124.1)
Dr Smad5 (NP_571443.3)
Hs Smad5 (AAB72180.1)
Dr Smad1 (NP_571431.2)
Hs Smad1 (AAC50790.1)
Dr Smad9 (NP_001315428.1)
Hs Smad9 (NP_001120689.1)
Hys Smad5 (XM_057436736.1)
Nv Smad5 (NP_001296671.1)
Nv Smad1 (ABC88374.1)
Smad1/5/9
Nv Smad3 (XP_001631657.1)
Bl Smad3 (CAH1251607.1)
Dr Smad3a (NP_571646.1)
Dr Smad3b (NP_778258.1)
Hs Smad3 (AAL68976.1)
Dr Smad2 (NP_571441.3)
Hs Smad2 (AAC39657.1)
Hl Smad3 (KAJ8036411.1)
Hl Smad3 (KAJ8036527.1)
Dm Smad2 (AAD11458.1)
Hsy Smad3 (XM_057460141.1)
Hv Smad3 (XP_012561976.1)
Ce Daf8 (NP_492321.1)
Ce Daf14 (NP_001255476.1)
Smad2/3
Dr Smad7 (NP_778257.2)
Hs Smad7 (AAL68977.1)
Pl Smad6 (ADW95340.1)
Dr Smad6b (NP_001038516.1)
Dr Smad6b (NP_001019981.2)
Hs Smad6 (AAC82331.1)
Bl Smad6 (CAH1251580.1)
Nv Smad6 (XP_032236464.1)
Dm Dad (BAA22841.1)
Smad6/7
1
Cnidaria
Deuterostomia
Ecdysozoa
Lophotrochozoa
ML Bootstrap 0 to 100

Fig. S8. Smad phylogeny

Unrooted topologies of Smad multigene family obtained by phylogenetic analysis using Bayesian inference (**A**) and maximum likelihood (**B**) methods on amino acid sequences. The two analyses give rise to similar topologies with four major clusters: Smad1/5/9, Smad2/3, Smad4, and Smad6/7. Smad6/7 and Smad4 are sister groups in the Bayesian analysis, while the Smad6/7 cluster branches first in the maximum likelihood topology. The *Hydractinia* genome encodes four Smads including one Smad1/5/9, one Smad2/3, and two Smad4. No Smad6/7 was detected, in contrast to the cnidarian *Nematostella*, which has one. Nodes robustness are posterior probabilities and bootstraps (500 replicates) for Bayesian and maximum likelihood analyses, respectively.

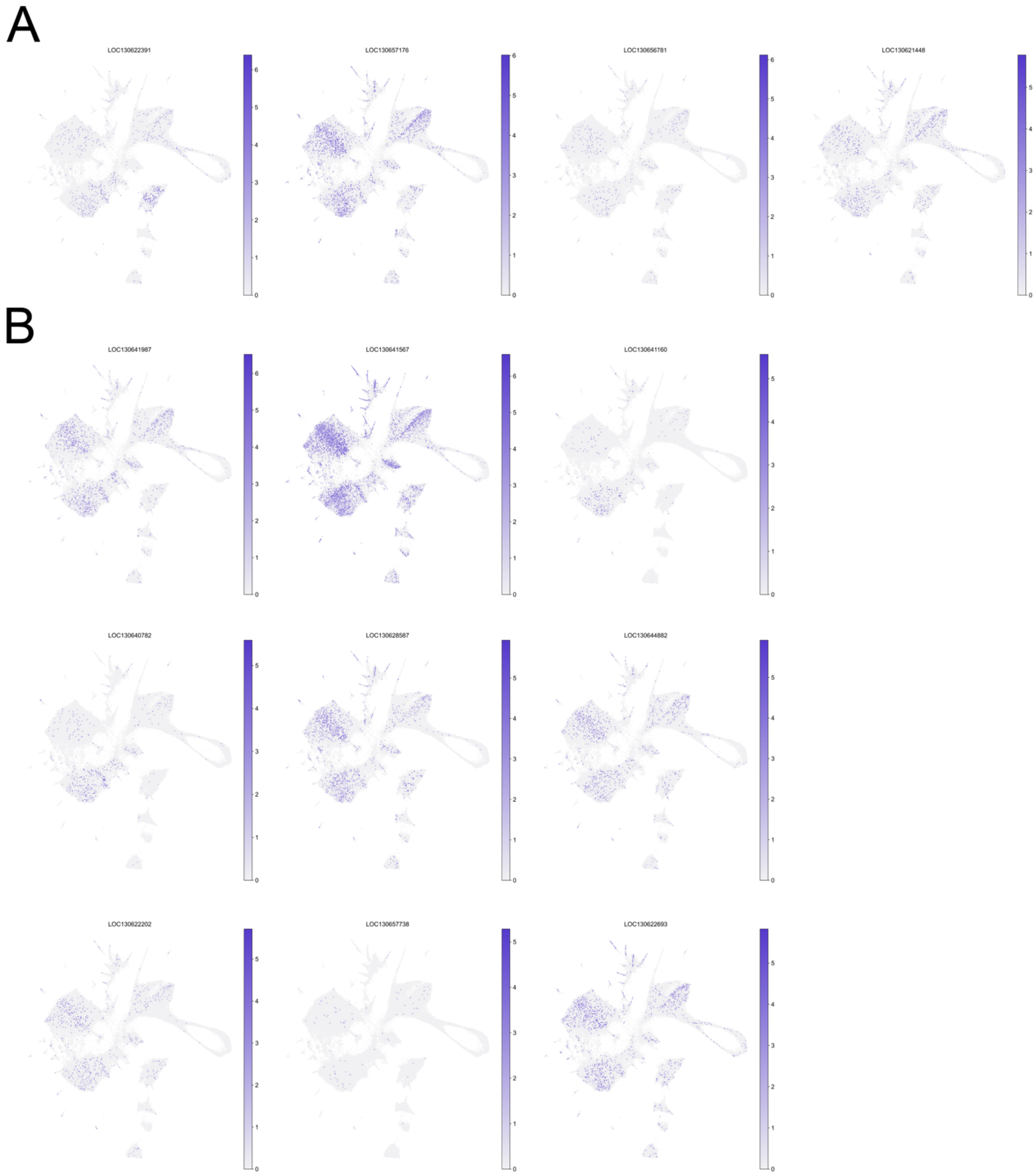
A
LOC130622391
LOC130657176
LOC130656781
LOC130621448
B
LOC130641987
LOC130641567
LOC130641160
LOC130640782
LOC130628587
LOC130644882
LOC130622202
LOC130657738
LOC130622693

Fig. S9. Expression plots of Smad and TGF-β receptor genes.

UMAP expression plots in the *Hydractinia* cell type atlas. Dataset is composed of tissue from all colony parts, including feeding polyps, sexual polyps and stolon. (**A**) *Hydractinia* Smad genes. (**B**) *Hydractinia* TGF-β receptor genes. All genes plotted show an expression pattern which is not confined to any broad or specific cell type.

Table S1. Percent of sexual polyps in wild type colonies.

	Number of feeding polyps	Number of sexual polyps	Total	% of sexual polyps
Female 1	66	53	119	45
Female 2	162	170	332	51
Female 3	145	161	306	53
Female 4	259	62	321	19
Female 5	140	63	203	31
Male 1	102	234	336	70
Male 2	282	81	363	22
Male 3	123	145	268	54
Male 4	129	71	200	35
Male 5	84	185	269	69

Table S2. Quantification of BrdU+/Piwi1+ cells in the gastrodermis of Gls$^{-/-}$ and Gls$^{+/+}$ polyps

	Piwi1+	BrdU+/Piwi1+	% of BrdU+/Piwi1+
Gls-/- Female 1	3	0	0
Gls-/- Female 2	5	0	0
Gls-/- Female 3	5	0	0
Gls-/- Female 4	6	0	0
Gls-/- Female 5	2	0	0
Gls-/- Female 6	4	0	0
Gls-/- Female 7	1	0	0
Gls-/- Female 8	6	0	0
Gls+/+ Female 1	17	2	12
Gls+/+ Female 2	15	3	20
Gls+/+ Female 3	23	5	22
Gls+/+ Female 4	24	7	29
Gls+/+ Female 5	14	3	21
Gls+/+ Female 6	19	4	21
Gls+/+ Female 7	27	6	22
Gls-/-Male 1	3	0	0
Gls-/-Male 2	2	0	0
Gls-/-Male 3	5	0	0
Gls-/-Male 4	2	0	0
Gls-/-Male 5	1	0	0
Gls-/-Male 6	4	0	0
Gls+/+ Male 1	67	19	28
Gls+/+ Male 2	56	23	41
Gls+/+ Male 3	59	18	31
Gls+/+ Male 4	51	15	29
Gls+/+ Male 5	33	5	15
Gls+/+ Male 6	48	20	42

Data S1. Sequences of transgenic constructs and protein alignments used to generate the phylogenetic trees.

Plasmid sequences:

- 5’β-Tubulin::Gls-T2A-mScarlet::3’β-Tubulin

ACAACGATAAACAAACATAGCGTTATGCTGTTGTTTTTTTCAAAAAAAAATTTATACTAATCAAGTCTTTTTATCATGTTTTATA
AATTCAGCGTTTTTATTTTTCCCTGATTCAAGCGATAAGGTCATCTGTTTGTTTTTCACACCATCATGTCTTCAAAAGACGTA
CATAAGTTTTCGTGTCATGTTCACATGACAACAATTAAATAGATTTTTATACTGCTTAGGGTTTGTCAAAAGGAGTGCATTTT
TAACTCCAATAGGGTAGTAACAGCAAAGTGGATAAATCCTTTTCTTTGACAGTTGTAAGGCTAGTATCAAAGTATTGTTAAG
CTTCAGGGTTATCTGACACGAAATATATTGTTCTCTATTACTCAGACGGTGTTTTCATTACCTTATGTTTTATACGGTAAAAA
AAGTTTTAATCTTAAGCAATTATTTTAGCATCTCCAGATGCAATCTTTTGTTGTCTGCAAAAATCTAATTAGAATTGTCATTAC
GTTTCCATGACAACTCTTATGAAATTACTGCTATCAATAAAAGTCATTAATAAAAGCTGTTTCATGGTCACTGAGGAGCTTTG
TAAAAACTTTACCAGTAGAGGGGCGTAACATTTTCAGATAATAGCCATCTAGAACCCAGTTTTCTGATGCTTAAAAACCTGG
GTACAGAAAGCTAGTTTATTCTAAACAGCTACGAAACACGTTTTTACCCAGATAATTTCTTTGTAAATTTCCTGCCCGTTTTC
TCTTTAAAAGAAAGACTACTTTTTTAATTTAGTGACTTACACGAAATTCGAATAACGCGACAGAAATTGTTAATACTGTTAATG
TTACACGTAGAAAACCTGTAGAGTTCTGCCATTTTGAATGTGTTTATTTGTTTTAGATGACGTCACAAGAATTTCATGAAGAT
TTGGATATGTTGCTCAACATGGAAATGAAGTTATTACTGCTCGATTTAGAAGATGTTCGGATACCGGAAAATCCTCCCGCAA
TACCTCGTGAGCCGGAAAATTATAATTTCTACTACAAAGATGATTAGAAACGATAACTGTCTACTTTGTCCTTCCAGAAACTT
TATCGTTTTTGCTCAATCCAGCTATTTGAACGTTTTGAATAATCATCGAGTGAACGAATATGGCGAAAGTTTTCTTTAAGTAA
TTACAAAAATTTAAATTACGAGGGACCAGAACTTAGTCACGTGACTGGTGACAACGCATACTCTTTTATAAAAAAATTTTTCT
GCCTCAAAGCAATAATTTAATTATAAAAATTTCAAATTCTTTTTTGCTTGTTTCTGCTCAGTTTTTACTGAAAAGGATTTATTT
GCAGCAATTTTAATTTAATCGCCGTTTTTTTCAGTTTGGGCTTTTTTAAAACGATCCATGTAGGTTGAATATTCTTATAGCAAT
CGATTCAGCTTTAAGTGGTTTTGAATATTTTACCATATAAAAACAAAAAAGTTTGCGGCTGCAACAGACCGACACTTGGTCT
CTGTTATCTCAAATTACCTGGAAGGTTAACTGTTTTGCGATGTTGTTTTTCTTCACCAGTGATGGCTTGAGCAGAGGAAACT
CACGCAGCGTCCTCAAAAGACATTTCAATTTCAATGCTTTTTTGTCGCAACTATTGTCTATATTGTGATAAAGAAAGATACTT
TTTTTTTTAAAGTCTGAATATAAATTTTTGTAATATTTTTTTTCTAAAAAGATGACAGCAAAATTGTGTAAAAATAAGAATTGTA
TAAACCTATTGTGTTTTAGTGCTTTGAAATTTATAAAATATTTTTTGTTGACAGTTGCAACTTTGTACTTGTATATACACATCTC
AAAATACATAATTGTAAAAATTCGCTGTGAACTATTTTTATCTGCTCCCTCTGGTCCACATATTGCATATCTAATATATATTCT
CTCGTTTTGAGAGATCATTGTTAGATCTTATTATTTCCGCGATAACATTTTAATAAAATATATTTTATCGGTTGATCCTACTGA
CAGATGCTTCTTATAACAGCCGTCCAATATTCTTCATTAAAATTATATTGCAGAACCCACCTTTAAAACACTCCAGGGTGGAA
TATATTTATTTACTAAAATAAAATTATGGCTTATCCTACATTATTTAGTAATGAGAGTTTGAGCACGGATCACATTTACTTGTC
GTTTTTGCATAAATATATTTGGCACCTCTCATCACTGGGTGGAAAAATTTCATTATGGTTAGCTTTTCGGAATTTTTTAAGCG
GCAGATTGTGTTTGTGGTAGGATGTTCAAACATCTCTTGTTGTTGAAACTACCAAGCTAGAATTGAAAATAATAAATCATCGA
AGATTCCTACGGATTTCCGAACTACATTTTTGTTTAAGGAGTTTTGTAAATTCCACATGTGGACTTTGAATGTTTTTTACATTC
TGGAAAACTATTTTTCTGTGAGAAAAACAAAATTTGAATTAGTTATGCTTTTAAAAATATTCCTTTTGACATTAAAACGTGTTG
TCTAAAGTAAATTGAAAAGATCTCGCCTCATAAATAATTTCTATTCAACCCACAAATTTTAATTCAGTACGTATTCGCTTACTT
GTAAACGTAATTGCCACATACATAATTTACTGTTTATTATTCTTGATGATGAATATAAACTTCGAAACGTGCGTTGAATTTTTA
TTATGAATCGAGAAAAATCAAAATAAATTAAACCGCTCGTGAAAACCACTTCCACCGCTGTTTAACATTGAGGTGAAAACCT
ATTAGGCACAACCCTCTAAGCAAAGACCGAAAAATATCCTAGACTCTAGTTTAAGCTTTTAACGCCGTACCAGAAAACGTTT
AAAGTACAGCGATTTCTTTGTTCAACTACATATTAAAGCCAACCTTGTCCCCAGGGCCACTTGTCTTTTACTCTACGCTAAA
GAAAAAGGCGAAACAGTTCCTGGGCACGAAGTTGATATGAATATGTTCCCTGGTAACTTGCAAATAAACGTACGATCGTGA
TTCTCAGCTGATGATTGAATGATAACAATCTGTTTTTATGGATGTTCTCAGGAGGAAATTATAGTCGATTAAAAAAAATGTCC
GTTGTGTTGCAATATGTGTTTTTAATTAGCAGGTTTTTAAGCCAATTATATATAGGACTGTTAATTGGTTTGACGAGTAAACA
ACTACCTTCTCGCAGCAAAATGCAAAGTAGCTGCACAACCTAAGAAACTAAGAAAACTTGGCTGAGATTTTTTTTCTAAAATT
TAAAAACAAATTCCGGAACAAAATAATGTGAGTCTCGAAATACTAAAAACTATTTTTCTCGGAACATTAACCGTGCTATCTCT
TCCAATCCTATGAAGTGTTCGTCATCTTCTGTAAAATTGACCCAACCTTGTCCCTAGGCTAGTTAGGTTTGTTTATATTTGGA
CGACCGTCCAAAACACCGAAGAAGCCCTGGGGACGAGGTTGAAACTGACTATCCGTTTTCCCTGTTTTTAGAAGAACCCAA
ATATGAACATTCGAGACTGAAAATGTGATTTTAAGCATGATAAAAAAGAATTAACATGTCGAGAGCTACTATTTGCTTCTCCT
CCAGACCGCTGAAGAAATATTTTTCCGCTTTGGCACGACTTTGTTTGTGGTGAGCAAACACGAAAGCAAATGCATATTGGA
TGAATTTAGATTTATGGATGCTGGTTGAAAAACAGCGTGAATTATTCTGCAAACGTAATGTAACTTCGAATTATTTATTAACA
AAACAATGTATAGTCGCAGCTAGATTTTCAAACTCTGTCTTTTTCCACGATCCTATCAAATACTCGCAAGAAATGAAAAAAAC
TCAGTTTGTAGTCGAGCCGGCTACAGCTAGTTAGCTATTTGTACATAGTTTTTAATTTGTCATTTTTATTAGTGCCACACCCC
AAAACCTCTTCTAACAAAAATAAACCCCATGTTACTTAAAAAAGAGAGAATTAGAGGGCGAAAATTTCCTGCATCGCTAAAT
CATAAGAAACATGTATTATTTATATCCAGTTGTCAACTCGGTCCACAGGGTAAACATTGCTCGGCTGTCATAGTGATAAATC
TACTTTGCATTATGTTGTCCGAACAACATAACAAAAAACGCGCCAATCTTTCTTCACTAGCCAATTAAAATAAACATCCAAAG
CACTTTAAAAGTTGATTGGCTTATAAAAGAACGCACTACAATGAACGCCTCAGAAAACAGACGGGGGTTTTATTAAGCATTT
AGAGTTTAAGAATTTCACCATGTTGCCAATATTTCAGATAATTTAAAGTTTTTTTATATAAAAAAATATTTTTTCTTATATTTCAT
GCAGAATTTATAATTGATGTTGCTCTAGTATAGGATCTATTTGGTAATGATTTTCAAATCATTACCCCCCGGTTATACTTTTTC
TATAGCAACAAGAGGAACCGATTTTGCACAACCATCACAAATCTTTCATTGCAAGTCGCAGGCGGCTCAGAGAAGCTCGTA
AAAAAAACTCTTACAAACTAGGTGTTGTAGCACGAACTATATAATTTCAATGCGGCCGCCTGCAGGGGTGGAGGTGGATCT
GAAAAAATGTCATCGTTGTTAATATTTTTGATGTTACTAAGTGTGATCGATCCCGTTGTATTAGTGATTAATAATGATCCATCT
AAAGGCAATACACGAAATCCACAGGTGCCACAGTTTATGATGGACTTATACAATGTGGTAGCTGATGAGAATGGATACGCC
AGAAAAGGTGCTCCTAATCTTGGAAAAACAATCAATTGCGTTTCGGCTCACGATTCAAGACATAACAGCACATCTGGCCGA
TTCCGTTATCAATTCAACCTAAGCTCGATTGAAGCAGACTTGATTATCCGAAGGGTTGAGTTGCGTGTATTTAAAACAAAAT
CAACACGTCGAGAAAATGTATTTTACAGAACAGATATAGTCCTTGACGGTCCATCGTACCGAGACATTCGCTCAGTAATGG
TCAGCTCAACAGGTTATGGTTGGTTGTCGTTTGAAATTACCGATGTTGCAAGTCGATTAATTCAATATGGGGATAAAATCGT
GGATATTGATATTACATTGCAACCAATTCGTAACAAACTTCCAACATTTAAATTATTAACATCAACAAATACGTCTACAAGAG
CCTTATTCATGGTGTATTCCGACACAAACGTCAAATCATCTAAGAAGGCGTCAAGATTGTACGGCACAGGTAAATACGCAG
GTGTGCAAGAAAAAAATCGGCATCGGCGCGATGCTAAAAATTCCCGTCAAGCTTGCCGATTGGCTAATCTGACAATTACAT
TCTCAGAACACACTGCATTTGGTGATGTCATGTTGCCACGCAAGGTAAACATTCGCAGATGTGAGGGATCGTGTCGATATT
TGGTTGGAGCTGGAACAGACGTAGAAGAAACTTACCATGGTGTCATGCAGAATATGATTAAACGCGAACAGCCGAGTCATC
TAAAAACGAAACGCATCCGATACGCCTGTTGTGCTCCCCGTTATTTGCAAAGTTTGTTGTTGTTAAGACAGAGTTCGATAAC
TGGTGATATCGTCATGGTTACATTGGACAACGCTATTGTAGAAACATGTTGGTGTGTGTACATTGGATCAGGTGAAGGTAG
AGGATCATTATTGACATGCGGAGTTGAAGAAAACCCAGGTCCAATGGTATCTAAAGGTGAAGCAGTTATAAAAGAGTTTAT
GAGATTTAAAGTTCATATGGAAGGTTCCATGAATGGACATGAATTTGAAATTGAAGGAGAAGGTGAAGGTAGACCATACGA

AGGAACACAAACAGCTAAGTTAAAAGTTACAAAAGGAGGTCCACTGCCATTCTCGTGGGATATTTTATCTCCTCAATTTATG
TATGGTTCCAGAGCTTTCATCAAGCACCCAGCTGATATACCGGACTATTATAAACAAAGTTTTCCGGAGGGATTCAAATGG
GAAAGAGTTATGAACTTTGAAGACGGAGGAGCTGTTACAGTAACCCAGGATACCAGTCTGGAAGATGGTACTTTGATTTAC
AAAGTTAAATTACGAGGAACAAATTTTCCGCCTGATGGCCCTGTAATGCAGAAGAAGACAATGGGTTGGGAGGCATCGACT
GAGCGTTTATACCCCGAAGACGGCGTCTTGAAGGGAGATATAAAAATGGCTTTACGATTAAAGGACGGTGGCCGTTATCTA
GCTGATTTTAAAACGACATATAAGGCAAAGAAACCCGTTCAAATGCCTGGTGCTTATAATGTTGATCGGAAATTAGATATCA
CATCACACAACGAAGATTATACAGTTGTCGAACAATATGAGCGAAGTGAAGGCAGGCACAGTACTGGAGGAATGGATGAA
CTATACTAAGAGCTCACACTTTTCGTTTTAATTTATAATTTTAAGTTTTGCTTGTATATCATGAATATAGGGAAGGCACAGAA
GTAACGTAAATGATAGTATTATTCTTTATTCCAATACTTAATAAAGCACAAGGCCCAAAATTTGCTATGGGAAACATTTACGC
GCTTAAAACATAAAAATCAAAAGCCTCCGTTTCCAATTTTTTGCGTTTTTTGAAATGTCATTTTCCTGTACTTGAGAAACCGA
AAGATACTATATAAAAAAATGGGTTCAAACCGGTCGCCAGTGGAAATCCTATCAACGGAAATTATACNNATTCATTGCTTCA
ACTAATAAGTTCGTATGTCGAATAATAAAATGATTTCAGACCCAAGTTACGATTAACGGTTCTTGAATTATCCTATTGTCGCT
AGAAATTGGTGCAACGACAAACCTCTAATTCGATGGCTCGAGAATTCGAGTTATAGCACCGATTTCGCTTCACACATACTGA
CGGGTTTTGATGTTGCACTGTGAAATGCAAAGCTCGTCCCATCACCTTTAACCCTGGCTGTATTAGGGGCAGCGCTGTTGA
GAAGAAAAAGTCCTGGGGACGAGGTTGAAGTCTTGTCGAGATAACTTAGTTCATACTTTGAACTGGGTTGTACTACAGACA
CATCTCTTTGTGGACCTCTGTCCGAACAATTGATTCATATGTTTAAAACCTACTGAATACCATTTTCTTACTATTTAAACTTCA
TTATTTTTGGGAAAGGCCATTGCTTAGATGCAACCTCGTCCATAGGGTTTTTGCCTTCTTGACATCAAGTAAGCGCTAGCGG
CTNNNNNNNNNNNNNNNNNNNCCTGGGGACGAGGATGGCTTAAATGGGGATTCATCCAACGGCCCACACACAGTGTACCA
TCTCCCCATTTGCCCTCACATGGTTAGGGGTAACCCGGGGCTATGGTAACATTCTCTCGCCCATATTGTGAATAGCTGTCA
AGGGAAACCGAACCCCGGTCTGCCGCACAAAGTGCGCGAGTTATAACCAAAGCTACGGCGCCAGTTTTAAGGGCAATGTC
CGTTTATGCCGAATGTGACGCCTCGGTTGCCCACGATGGCTCCTATGGCTCAC

5’β-Tubulin/3’β-Tubulin, Gls, T2A, mScarlet codon optimized

- 5’NcoI1::Gls-T2A-mScarlet::3’NcoI1

TTGTTGCTATCGACACATCAGAAATGTTCCTATTATTTTTATTAGTCAGGAACATGCTGAACTGTTTATCAAAATTTTAAGAAA
TTTTTATAATATTTTAAGACTAATATTGGGGCTCGCTTTTATGAGATTATTTATCTGTTCTTTTTAAAATAAGAAACATCTTACA
CAGTCATTTTGTAATGTATCTCCTTTCAAAAAACAGTTAGCAGTTTTGTGTTTCCGAAAGTTTTATTAAGAAATAATGCGTAAC
TAGATCGATACAAAATTATTATAGCAGATTGATTGCATCCATCAGCCTGTCCGAAATTATCTCACAGCAATTTGGTGCGCTA
TTGTAAAGCTGAAAGTCGAAATTAACGTTATTTATTTTGTTACAGCCTGGTTTTATGAATTTTATTCACTTGTTATATTTTTTAT
CTTTTATTTTTAAAAACAATTATTATAAAATAGAAAGCGTAGCACTTTTGAAAAGAAAGGAACGCGGGTGTAAAAACAACAAC
AAATAGTGTTGTGATTTTGACTGTGTGTAAAATGCTCTATTTTCAAAAAAACAGACGTACCTTCTCTTCGTTTGCGAGTGGAG
ACGTCTAGATAAAGATTTTATTTAACTTCGACGGGAACTAGATCCAAGCTCATCCTTTCTCATGCAAATAGACAGAAAAATTT
TAAAATTTAAATTTGTTTTACTGTTTATCGCCTCTCCCAAAAAAAAAAAAAAAAAAGGAATTTAAAAATATAGATATTTGAAATTC
TCTTAACCTCAAAACTTTTTTAAAAATCAAAAATATATATTATTCGTTGAAATCTGTGCAAAAAAAGTGTCCAAACTTTTAAAA
CTCGTCAAAAGTAAAAACGTTTAAATTTTTTTCACCTAGAACGATTTTATGCGCAATAATAAATTCACTGCGTTCAAACGATC
GCGGAAGAAATCGAAGCTCGTGTCAAATGGAATGCTGCACAAAATAGTACCATCTATTGAGACACTAGTAAATTTTCGAATG
CTTCGTTACTCTCACAATTTTTTACAGGGGGTAAAAAAACAGAGAATTTAGTATGAAAACAGAACAAAAATACGACGAAATAA
CTATTTCTTATGGAAAGTGTGTAAATTATTTTGAAAATGAAGTTTGAAAATTCAAATGTGGTTTAAAAAAATTAGTTAGGTGCA
GAATAAAACTATATCTAACATTTTTGTACCCCCTGATAAAATTGATATGTTTTTAATTTGACAACTGAATCGTAAAATTGTTCG
AAGTTGAGGATTAAATGATCATTCAGCATGAGGCTACGCTACAGCGCGCATGCCTATTACCCGGTGAGAAATGTCATGCTT
GGGTAACCCTGTGTGCGCATGATCAATTACATTACAAAATCAACCAATCAAATTACAGAAAATCATAAAATCTTGAGCAGCT
TACTTCTGTGCAGGTATAAAGAAAGTTATTTCGAAAAGTCTAATTATCATTCATACCTCAATTGCCATTAGAATTGGACGGAG
TGCGGAAAAGGGTAAGGTAATTATTTTTTATATAAACTGTTTCTTGTTGTCTTCTTCTTATTATTGTTTATCATTGATATATTGT
TTAATATTGTTACATACTATCGTATTTATATTTGTGTATTTCGAGTAATTACAACAACAAAATTTATTTTATTTGCTTTTCGCCT
TCACAATCATATTTTAAATTAAAATTATTTTTATGAGACCAAAAAATGTTTTTTTCTTATGCTCTATCTTACAAATTCTCCTTGG
TACTTCTCAGCGTGACTAATACACATTTATACTATTTAAGACCTACACAACTAAATAAACAAAAATAAAGATTTTAACTAAAAC
AATATTTTAGGAAAAAAAATATACAATACAAATATACAATCCACCTCCTAAATTAAATCAATAATCTGTTAGCACAAATAAAAT
ATAAAATACGTTTTCGATTTTAGCGATTATAACAATCCTACAGTAAGGCCTATGTCATCGTTGTTAATATTTTTGATGTTACTA
AGTGTGATCGATCCCGTTGTATTAGTGATTAATAATGATCCATCTAAAGGCAATACACGAAATCCACAGGTGCCACAGTTTA
TGATGGACTTATACAATGTGGTAGCTGATGAGAATGGATACGCCAGAAAAGGTGCTCCTAATCTTGGAAAAACAATCAATT
GCGTTTCGGCTCACGATTCAAGACATAACAGCACATCTGGCCGATTCCGTTATCAATTCAACCTAAGCTCGATTGAAGCAG
ACTTGATTATCCGAAGGGTTGAGTTGCGTGTATTTAAAACAAAATCAACACGTCGAGAAAATGTATTTTACAGAACAGATAT
AGTCCTTGACGGTCCATCGTACCGAGACATTCGCTCAGTAATGGTCAGCTCAACAGGTTATGGTTGGTTGTCGTTTGAAAT
TACCGATGTTGCAAGTCGATTAATTCAATATGGGGATAAAATCGTGGATATTGATATTACATTGCAACCAATTCGTAACAAAC
TTCCAACATTTAAATTATTAACATCAACAAATACGTCTACAAGAGCCTTATTCATGGTGTATTCCGACACAAACGTCAAATCA
TCTAAGAAGGCGTCAAGATTGTACGGCACAGGTAAATACGCAGGTGTGCAAGAAAAAAATCGGCATCGGCGCGATGCTAA
AAATTCCCGTCAAGCTTGCCGATTGGCTAATCTGACAATTACATTCTCAGAACACACTGCATTTGGTGATGTCATGTTGCCA
CGCAAGGTAAACATTCGCAGATGTGAGGGATCGTGTCGATATTTGGTTGGAGCTGGAACAGACGTAGAAGAAACTTACCAT
GGTGTCATGCAGAATATGATTAAACGCGAACAGCCGAGTCATCTAAAAACGAAACGCATCCGATACGCCTGTTGTGCTCCC
CGTTATTTGCAAAGTTTGTTGTTGTTAAGACAGAGTTCGATAACTGGTGATATCGTCATGGTTACATTGGACAACGCTATTG
TAGAAACATGTTGGTGTGTGTACATTGGATCAGGTGAAGGTAGAGGATCATTATTGACATGCGGAGTTGAAGAAAACCCAG
GTCCAATGGTATCTAAAGGTGAAGCAGTTATAAAAGAGTTTATGAGATTTAAAGTTCATATGGAAGGTTCCATGAATGGACA
TGAATTTGAAATTGAAGGAGAAGGTGAAGGTAGACCATACGAAGGAACACAAACAGCTAAGTTAAAAGTTACAAAAGGAGG
TCCACTGCCATTCTCGTGGGATATTTTATCTCCTCAATTTATGTATGGTTCCAGAGCTTTCATCAAGCACCCAGCTGATATA
CCGGACTATTATAAACAAAGTTTTCCGGAGGGATTCAAATGGGAAAGAGTTATGAACTTTGAAGACGGAGGAGCTGTTACA
GTAACCCAGGATACCAGTCTGGAAGATGGTACTTTGATTTACAAAGTTAAATTACGAGGAACAAATTTTCCGCCTGATGGCC
CTGTAATGCAGAAGAAGACAATGGGTTGGGAGGCATCGACTGAGCGTTTATACCCCGAAGACGGCGTCTTGAAGGGAGAT
ATAAAAATGGCTTTACGATTAAAGGACGGTGGCCGTTATCTAGCTGATTTTAAAACGACATATAAGGCAAAGAAACCCGTTC
AAATGCCTGGTGCTTATAATGTTGATCGGAAATTAGATATCACATCACACAACGAAGATTATACAGTTGTCGAACAATATGA
GCGAAGTGAAGGCAGGCACAGTACTGGAGGAATGGATGAACTATACTAAGAGCTCATGATGACAAGGAATTAGATATTTTT
CAAATTACTTCTTTTCTTGAACTGATACTTTTCATATGGTTATTGACACAGACGCGAACAGATTAGCATCTACTCGTCCAAGA
TAGATTGAATGGATTGGAATATATATTATATTGTTAAATTATAGTTTCAATTGTGTGGGACTTTCCATGCAACCCGGAATCTC
ATTATTTTTTTTTTGAAAGATATATGTGAATTTTAATGCATGATCATATGAAAGAATAAATATTTTTCTATCCATAAAATTTTGA
TCTTTTTTTCTTTCTTATCAAAAGTAATCTACAAGAATTGGACTAACTTGTTTTTGAATGCATTTAATTTTATTTCCAAAAAGAT
TTACAAAATATCCAACGCTTCAAAAGTATATAATTTTTCTACAAATAACATTTTTTCTAAAATTAACTACAAACAATTATATATC
CCTTGAGATTTAAACTTCCGAGGTTTAAAAATAACCTTGCAGTACTCCATGATTATGGTATTTTTTACAGAAAATTAAGCCTA
ATAGATCAAAAATTATGAACACACTCATGGCCTAGCTCTAATAAATGCTAGAAAAGCAAGTTAAACATCTGCTCTAACAATTT
TGGCACCCAGTTATCGGGAAAAAAATAAAAATCAAGCCAGCAAATCGCTCTACATATCTCAGTTTTCTTGTTGTAACCATAT
GTTGTAGAAATCTTCGATAACTCAACGCGTCATTTCTGCATGATCTTTATAAGTTGAAGCTTGAAAAAATATGCCTAACTAAA
TCGTTTGTTTACTGTCAGGAAATAAAATTTTGTGGCAATGTCCAAAGATGGCGTTTAACATTACCTAGACTAACATTTGAGTT
CTTTTGAATTAAAAACACCAGAAATACATTCAGTCGAAGGTTTTCTTTGATCAAAATTTCGAAGGTTGTATAGAAGGCAGAGA
AAGGGTTTGACAGATTCGCTATTTCTTGTTTTGTAAAAGGTATAAGCGTTGCAGTAACTACTACGAAAACACGCATCGAAGC
ATTGACTCACTGTATCAACGTGAGTATAATTTTTGGTTATGATAGCTAGGTTAACAAATTTCTTGTTATACAGGAACCCGTCC
TTATAGACAGCATTCTGGCAATGTTCTCCAAAAATTGCGTCACTTGTTAACAATATCCAGTGAATACATAGATAAAACAATGC
GTGATTTAATTGGATGAACATTTTATTAATCAATCGGTTTTCTTGTCAGATTTTCTTTGTTAAAAAACGAGTTACCTTGAGTGT
TTCTTGATCACCTTAATTTTCCTTTTAATTCTTCTTTTGTTTAAAGCATTTTGATTTTTCCAAACATCTGCAAGTACCCCTACTT
AACTTTTCAATCGTAAACTAACAAAACTTACTTCTATTGTGTATGTGTGAAGTCTACTCATAAACTTG

5’NcoI1/3’NcoI1, Gls, T2A, mScarlet codon optimized

- 5’Wnt3::Gls-T2A-mScarlet::3’Wnt3

CGAGCTGGAGCTGTGTTAGTAGTAGGTTAAGAAAAACTGGAACCAAACTCCTTGTTAGTGAACTATATTAGCTAATATAAAT
CTGTATGATATATCTTGAAATTTGAAACCAGAAAAAAAAAACAGTCAATTTTTTATNTTTTTTATCTAACCTTCATTTTCATAAG
TGTTACCGAGGCTTGAATTGGTGCTTATAGAACAAAAACTGTGTATGCTTGCTATGAAAGTTGCTATGTATAAAAATACCATA
AATCCAAGTGTAAACTTATTTTTCTTGCATATGGTATGAGGGCTGTTAAAATTTGTTAAAAATGTACTGCATCACGACATTGC
CTTGTATAATGCATTTGTTTTTTTTAAAACAGTGGTGTACCGTCATCTCCTCTCGAATTAACGCCCAGTCTGAAGCGAAATCT
TAATAAGCGCCCGGACGGGTATTTGATTTCAATCGAGTATTTACAGCGTCTGAAAATAAAATCCTAAACTTCCTATCATCAAT
GCTTAAGAAAAGATATTGTTGCCGAACTTTAAAAAGTTGAGAATGTCGTCAGACTGTATGTAACTTTTGCAGTCGACTGTCG
CGTGAAGAGGAAATTTATCAAGAAAAAATTTGTGATTGGAATAAATGTTTGCCTAACTACATACGTTTATTTGCACAAATTGAA
TGACGAATACATGAAGTAAAGTGTATATCACGACCACGAAGTGTTCTTGTTTATAAATGGCGGATAAGCTGCGAAGTGTTGT
AGCTGGTGACGTATTGTAGCACTAAATGTTTTTATTTTTGTGTTAAATTTGGGTTGTTTGTTGTTGTTTTATAGTTGCAATGTT
TATGTTGGTTAATAATTTTTTTCTTTTTGAAATAACTTTGCACCCATTGCGAATTTATAACGCTTATTTTGACGAGAAACCAGG
GCTGTTGTTATTTCATGTTGCAATCACTAAAATCATTATATATAAGTCGCTTTTGCTAGAGAACACACTCGAAGTCAGAATCC
TGACCAATCTTTGTCAAATACTATCTACAAAAGGCAATAACAACGTTTATGCTTCCCAGCATGCAAAGGAGAAAAATACGAT
TACGTGGACACGTACGTATTTATTGATTTATTTAAACTTTCTCGGTTGACCATTTTCCTTTTTATAGATGTACGTATACAGCAA
ATCTTTCTCGATTGGGAATCATGAGAACATAAAATGCTGTTATTTAGCAAATAGATAAAGTCGAGGGCTTTAATCTCTACGG
AGAAGCACTTGATTAAACAAGTGAACGCGTGGGGAAGTCATGCGCCTTTAGAAATATTTTTTCACATCAAAAGCTTGTAAAC
TTTATTCGAAGTTATTCCGGGGTATAGAAGCATCAATGTCCTGTTTACTTTGAAGGGTATTCATGTCTTAAATATAAGGCCAA
AGCGAATAGGAAAAACATTCATTAAACGTCATAGCAGGATAGGTTTGTTGACGATAACCTTTATTGATAATGGAAGTTTATTA
TTTTTAAACGGGAAGGAAGAAGGTTCTGTTACTTTTATGAATATCTAAAAATCGAATATATGAACCAAAATGCACAATGCCTT
TTTTATTTATTTGAACGTTTTAAATAAGCGTAACATATCAATATCACTTTAATTTATGACCAATAGATTGAAAACAATAGGGAA
TTTACTTACAACTANAAATCCTTTATATATGTATATGTACACTCTAAATAGGTTGAATTGTTGTTAAGACTCCGTTTGAAGTTA
CCGCCACATATTCTGTGACATTCTTTACGTATACCCGATAGGTTGGATGCGCTCAAAAAAACTTTTGACTATTAAAGGAACA
TGCAATGATGTATATTAGATTAGAAAACAAATATTGTATAAATAATAATGAGAAATAAATGAACAAAGTTATGGTGAAGGTAA
AGTAGAGAAAGTATTCCAGAGCAGAAGGATGGAATTAAATATGTTTTGCGATCATTAAAAAACTAACAATAGACAGTCAGAC
CGACTGCTAATAAGACTAATTGCATTTTATTCTCCTGTGATTTTATACATTATTGAGAAAGCTGTGTTTTCTTGATATGACTTA
CAAACTGTACAGTCTTAGTTCAGGAGGAGACATGCCGGAGTAACTACAAATTTGGGAGTTTGGCTTCGTAAAGGATTAAAC
CGACCACGTAATGATAATGCCATTTCTCTTTTATTTTTTAGTAATAAATTATCTATAAGTGTCGGTTAATGAGAAAATAACAAA
GTTGGAAAGACAGTTTTGTAGCAAGCACTTTGTTGGAGAAGAAAGCTGTAATTGTAATAGATTTGAACTATTGGAGAAAGTA
CGTGAACTTGCTGTTAACAAACCACACTGAAAATTAATGCGGCCGCATGTCATCGTTGTTAATATTTTTGATGTTACTAAGT
GTGATCGATCCCGTTGTATTAGTGATTAATAATGATCCATCTAAAGGCAATACACGAAATCCACAGGTGCCACAGTTTATGA
TGGACTTATACAATGTGGTAGCTGATGAGAATGGATACGCCAGAAAAGGTGCTCCTAATCTTGGAAAAACAATCAATTGCG
TTTCGGCTCACGATTCAAGACATAACAGCACATCTGGCCGATTCCGTTATCAATTCAACCTAAGCTCGATTGAAGCAGACTT
GATTATCCGAAGGGTTGAGTTGCGTGTATTTAAAACAAAATCAACACGTCGAGAAAATGTATTTTACAGAACAGATATAGTC
CTTGACGGTCCATCGTACCGAGACATTCGCTCAGTAATGGTCAGCTCAACAGGTTATGGTTGGTTGTCGTTTGAAATTACC
GATGTTGCAAGTCGATTAATTCAATATGGGGATAAAATCGTGGATATTGATATTACATTGCAACCAATTCGTAACAAACTTCC
AACATTTAAATTATTAACATCAACAAATACGTCTACAAGAGCCTTATTCATGGTGTATTCCGACACAAACGTCAAATCATCTA
AGAAGGCGTCAAGATTGTACGGCACAGGTAAATACGCAGGTGTGCAAGAAAAAAATCGGCATCGGCGCGATGCTAAAAAT
TCCCGTCAAGCTTGCCGATTGGCTAATCTGACAATTACATTCTCAGAACACACTGCATTTGGTGATGTCATGTTGCCACGCA
AGGTAAACATTCGCAGATGTGAGGGATCGTGTCGATATTTGGTTGGAGCTGGAACAGACGTAGAAGAAACTTACCATGGT
GTCATGCAGAATATGATTAAACGCGAACAGCCGAGTCATCTAAAAACGAAACGCATCCGATACGCCTGTTGTGCTCCCCGT
TATTTGCAAAGTTTGTTGTTGTTAAGACAGAGTTCGATAACTGGTGATATCGTCATGGTTACATTGGACAACGCTATTGTAG
AAACATGTTGGTGTGTGTACATTGGATCAGGTGAAGGTAGAGGATCATTATTGACATGCGGAGTTGAAGAAAACCCAGGTC
CACTGCAGATGAGTAAAGGAGAAGAACTTTTCACTGGAGTTGTCCCAATTCTTGTTGAATTAGATGGTGATGTTAATGGGCA
CAAATTTTCTGTCAGTGGAGAGGGTGAAGGTGATGCAACATACGGAAAACTTACCCTTAAATTTATTTGCACTACTGGAAAA
CTACCTGTTCCATGGCCAACACTTGTCACTACTTTCTGTTATGGTGTTCAATGCTTTTCAAGATACCCAGATCATATGAAAC
GGCATGACTTTTTCAAGAGTGCCATGCCCGAAGGTTATGTACAGGAAAGAACTATATTTTTCAAAGATGACGGGAACTACA
AGACACGTGCTGAAGTCAAGTTTGAAGGTGATACCCTTGTTAATAGAATCGAGTTAAAAGGTATTGATTTTAAAGAAGATGG
AAACATTCTTGGACACAAATTGGAATACAACTATAACTCACACAATGTATACATCATGGCAGACAAACAAAAGAATGGAATC
AAAGTTAACTTCAAAATTAGACACAACATTGAAGATGGAAGCGTTCAACTAGCAGACCATTATCAACAAAATACTCCAATTG
GCGATGGCCCTGTCCTTTTACCAGACAACCATTACCTGTCCACACAATCTGCCCTTTCGAAAGATCCCAACGAAAAGAGAG
ACCACATGGTCCTTCTTGAGTTTGTAACAGCTGCTGGGATTACACATGGCATGGATGAACTATACAAATAGGAGCTCTGTC
AATTTGTGTGGTGCTGCAGTGTAAAATGTGATACTTGCACGGAGGCTGTTCCGAAATATACATGCAAATAAAATATGTGTAC
ACGTGTATTAATCTGATTGGAGGAGAACTTGAGTTATGATAAAGACGTACGCCAAAGTGAAGCTACGTACGCCAAAGTGAG
GCTATTCACTGAAGGAATATATAATAAGTGGAGCACATCCTGTAGTTAAAAGAATTTGTTGTATGCCATGCATAGTGAAGTT
GAAAAGTAGAACACAGCTTGATCTTACAGATCTGTTAAATACACAAGGATTACCATGGAGACGATTTTCAAAACAAAACCGA
CGACGGGTTTGTAGTTCATTTCCAGGCTCCTAATTTAAACTGGACCAGCTATGTATGGGTAGCTTAGCAACTAAAAATAAAA
AAACTCTTTCAGTCACGTATTTATTTATTACTTTATTTATTAAAAGAAAACGCTTTGAAGAGGAGGCAGCACCAGTAACCATG
ACGACCACACAAAAACATACGGAGATTTGTTGTTTTTCAATATGTAATTTATACTTTTTTGTATATATGTTTTCTCTCGTTTGT
TTGACAATTTTTCGATGTTATATTTTTATACATATACATATAATGTTAGTTTTATAAATTACAATTTTTATTTAACAAAGTGTGG
AGTGTACATAGTAATGCTATGAGATTTTCTGAGAGGTTAGAATATATATATATCACGACTGCTTGCAACTTCATAGTAGTGAG
CCGCCCTGTTTAGTTTAGACACTATACATGCGCCCTATCTTTCTGACCGACGCCCTTTACTGTCTCTTACCCTTATTGAACT
AAAAGAGAAAGACTTAGTCCTTGTAATTATGAAATTCCTCCTTGCCATGACCCAACCCTTTCTTCCTTTCCTCCTCGACTTCC
CTTATATTCAAAAGAATATATAATTAAAGACGTCTCATAATTTATTGAACGCTTGTAGAATATATTAAAGACCAAAGTTTTTTTT
CCAGACTATAGAGTTAGAATTATATAGAGAAAACAAAAAGTTTGTGTTTTTTATGCTTCCCAATAAGAAACCCTCTGCATAAT
AGATCGAAATTGACACCCCTGTGTAAGTTGGCGAAGTTTTGATAATAACCTTCTTTTAAAGGTAAAAATATACAAAAAATTCA
AACTTGTAATGCAACGCACATAACAACTACCGGGGCAACTCTGATGTTTTATGTTTTCTAAAACCGTTTGGTACACGTCATT
TTAAATATAGTTATACTCAGGACTATTTCTGGGCACCACTTTGAGATANTTTCAATTGTTTTCTGCAACACCAAAACATAAAA
TATGTTAGTCTTAATTATATTATTAATCGACTTTCCGTCTGTAAGTCAAAGATAATAGCTGCGAGTAGCGGTGGGTGACTCC
TTTTGAATTTCCGACGGCCAAACTCTCGTTGACTTTCTACGGGATTAACCATGATTGAGTTCACTTCTAAGACGTNTTGAAA
AACGTCGAAGGTAGACTATTTTAAAAAAATGTCATTTTTCAAACCAAATAGAATGAACATTACAAAAACCAGAAACATTATTA
GCACCAAAGTTTGAGGAGAGAGAAAAATCCATCCTTTCTAGGATTCTAGGTGTATATATTTTTACCAGTATTTTGTGACCATA
TAGTTTTCAAAGTTTTCAAGCCAATCAAGGGATGAAATCATGTAAGAAGAACTTTACGAGATAAAAATATTGAAAAAGTGCTA
TATTTTGTGGTCGCCCTACAAATGACAGCCAGGCTAATTTTTTATTGTGTAGCCTGGTGATTTTTCCGTGTTAGAAAAGCGG

TATTATGCTGAAATGAGCATGTTGGTAGAAATTAAAGCAATATGATTTGGGTTTCCCAAAACGTAATTTTCAACAAATATGTC
AATTTTATTTGGTTAGGNTGGTAAGATTGATTGATAAAAATAGTTAAAAACAGTTACGAATATAGCTAGTGGGCAAATTATCA
GGTGTCATGGTCTCTGAAAAAAAATCTTCTGGGGTTGGGGAATAATTTACCAAGGAGAAATTCTCCGGGAACACACATCAA
AGTCCTTGT

5’Wnt3/3’Wnt3, Gls, T2A, GFP codon optimized

- 5’Rfamide::Gls-T2A-mScarlet::3’β-Tubulin

GCCTCTCTAATCAAGTGGATGTTTCTACGAGTTAATCAACTAGTTGAGATTAATTGTCAACAAAAAGTGGGGAAATTTTCGA
AATTTTCAAAAGTTAAATTTTCCAAAATACGCTTCAAATTCCGGAATGTTCCAAAACCGGTTAAGTTTTAGAAGTCCTCTACG
AACACTTAAATCGAATTCAGCCATTTTTATGTTCTGGAAAATTTGAATAGTTCTAAGGTTTTTTTTGCTGTTCTTTTTTCTCTC
GAAACTGTAAAGGTATAAATGTCCTAGACAGATCGTTATTTAAACGACAAAGTTCCTCCATCGTCATTTTTTACGATCTAGCT
TTCATATATAATTTTAACGGTTTCTGTCATGGCAACGAATCAATTTTATTTTACTCAAGAATTAAAGCCGATTCTGAAAAAAAA
ATTACGTTGTTTAATGATGAAAAAACGTGTGTTAAATTTTTGATTTTGTAACACTTTGGGGAATTTAAGTAAAGGTTTTGATTT
ACCTTAATCAATTTCCGAAGTAATTACGACTGTACATTCAACTATCCCACTGTTTTAGAATTGCGACGGCTTAAATTCTGTCA
TGGTAGAGTTTTCCCGCTGAAAATAATGGAATCAGTTATGCGTAACGTGGTTTCAAGTGATAGTCACCAGGCATTCTCAAG
GTTGATTGAAATCCGTGTTTCATGTCAGTCCTAGGAGCAATGTAAAAGTGATATAATTTTTTTTGTTCGCATCAAACTATTAA
AGTTTATATTTGACCGTACTACGTAATCTTTCTTTTCCATTCGGTTTTAAAGATATGCAAGGCTGGGACCTTATGTTTACTCC
ATGATGAGAAATCCTAAAGTCAATTGTTTATATGAATAAAAAGAATAACAAAAAGCGAGCCAAACTTCATATCCTAAACGAAA
TATATACACGTTAAAAAGATACAGCGAGAATCCAATAAAGACGGAAATTGTGAAAAACAAAAAGTTAAAATAAACTTCTTTGT
CTTTTGAAAAACAAAACAAAACAAATTAAACTATGAAATAGGAATTGATAAAAAAGAGAAATTTTGACGAAGTAAAATGTTTTA
ATTAGTTCAGCCATCTGCAATGTAATTCCATTACTTCTCCGCTACAATGGACTTCATTATTATAACAAAAAAAGTCATTGTGC
AAATTTAATTATCCGTGTAGAGGATGAAACATTTCACCTTGCTATGATAACAAGACCTCCCTTTTTTGTTTCCGTTCCGTACC
AATTTTAGATATTATATAAAAATACATCTCTATTTCAGCAGAAGTAATTTTTGGGGCGGAACACTGTGGGAATATATTGACAG
AAAGTCGTTGCTGCGTGAGTGCTGGCACTCTAATATCTTTCACTTTGAGAAAATTATCATCTGGTAAGTAAAATATTTTGTCC
TGCTAACTTAAGCTTATACATCTTCGAAATTTTACTGCAATTATCTAAAACGCCGTCTAAATATTTGCGGAAATCAAGTCTTC
AATTGTTGACGCTCGTAAAATTATGAAACCGTTTTTTAAGTAAAAACTAAATTTTAAATATTTGCGAATTTGCTAATTTTAAAA
AACGATTTTTTCTGATAGTCGATCATATTCAAAGCAAAATATCTTTTTCAAACTCTAGTAACAAAAATTCAATATGAAAAAAAA
AAAAAAATTATTTGGATTCAGAAAATTATACTCTTGAAAAAACGAATTGTTAAAATTGAAAACCCTTAAAAATTATTTTTTAAGT
TCTATTCACACAAAAATTCGGTTTTCTAAGGTAGATTATTTAAAACGAATAGGTAACAACACTTTTTTTCGTGTCGCGTAAATT
CGACAGCGATGTAGCAGGAAAGAAACGTCTTCACTTTTTGTGGGAAGAAAGAAAAAACGCAATTCATGAGACCTCCATGGT
TTGCCGAAAGTATAATATCAACAACAAGCTAGATATGTTGTGCCCTTTGCACTTTTGCCCAGTAATGCAAAATGGTTTATTAT
TTTTGCAAGTTGTGATTGCAGACCTTTTACTGGTTTTTTAAACGACAGTTCACAAAGAAGGGTTTGAGACAAAATGTTACATA
AATTCACCATTTTTAACTAATTTCCACAATTGAGAAACATGACATAATCGTAAAAACAATAGTTTAGCATACAGTAAGTTTAAA
ACTGTATATTAAAGCTCTTAATGTTTGAAACTTAAACTATTGTTAAAAATATTTTTTATTTTTTTAAAAATTGAACAGAATTAAA
ATTGTTTCACTACAAGTGTGTTTCTCTTTAAAGAAAGCACTCCCAAAGTTGATGTTTCAAATTACTGATGTTTCGAGCTAACT
TTACAAATTATGAAGTATTATAAAAAAACAAGGAAATATCATCTACGTGAGATTTTTGAGAAGAATGAACTTCAAGCGATAAC
ATATCATAAACAAAAATCAGCATGTTGAAAAACAGGAAAATCAAAAAAAGTGTAATTTATTCCGATCAATAGATAGAGTCAGA
TCAACTAATTAATTCAACTTCAAAAAACATCTGCACACTTTCTCTCTCTTTTGTTTGTCAAATAAGAAAATGAAAAAGATGAAA
AACAGGGACTAATTGGATAAAACAATCAATTTTTTTTATTATACATTTTCGCCTTATATAAATTATGACCCTAATATATTCACTTA
GCATAGAGCAAGTCAAGAGAGAAGGGTTTGCTATCCTCGAAGGGTGTGAGTATGCATGTTCACAACTTTATTTTTAATATAG
TCCTATCTTCACTTCAATATAAAATCTCATCAGGCATAACAATTGAACACTTCGATAGTCTTTGGTATAACATATCTGGAGGA
CAACATACACAATACGTTGCTCATGCATAAAAGAAGTTCAAGAAGTGTGCTGTTTTTATACGATGTTTCAAAACTTCTAACAT
GTATTGGCACCGTTTTGTACTAGTTTTTTCTACACTTCTGAATTTAAAGTATAAATAATTTTGATATCAGAGAAATGTGCATTC
TTCCTCACGAAGTGAATATTTTATTTCTGTAGATAAATGGCGGCCGCATGTCATCGTTGTTAATATTTTTGATGTTACTAAGT
GTGATCGATCCCGTTGTATTAGTGATTAATAATGATCCATCTAAAGGCAATACACGAAATCCACAGGTGCCACAGTTTATGA
TGGACTTATACAATGTGGTAGCTGATGAGAATGGATACGCCAGAAAAGGTGCTCCTAATCTTGGAAAAACAATCAATTGCG
TTTCGGCTCACGATTCAAGACATAACAGCACATCTGGCCGATTCCGTTATCAATTCAACCTAAGCTCGATTGAAGCAGACTT
GATTATCCGAAGGGTTGAGTTGCGTGTATTTAAAACAAAATCAACACGTCGAGAAAATGTATTTTACAGAACAGATATAGTC
CTTGACGGTCCATCGTACCGAGACATTCGCTCAGTAATGGTCAGCTCAACAGGTTATGGTTGGTTGTCGTTTGAAATTACC
GATGTTGCAAGTCGATTAATTCAATATGGGGATAAAATCGTGGATATTGATATTACATTGCAACCAATTCGTAACAAACTTCC
AACATTTAAATTATTAACATCAACAAATACGTCTACAAGAGCCTTATTCATGGTGTATTCCGACACAAACGTCAAATCATCTA
AGAAGGCGTCAAGATTGTACGGCACAGGTAAATACGCAGGTGTGCAAGAAAAAAATCGGCATCGGCGCGATGCTAAAAAT
TCCCGTCAAGCTTGCCGATTGGCTAATCTGACAATTACATTCTCAGAACACACTGCATTTGGTGATGTCATGTTGCCACGCA
AGGTAAACATTCGCAGATGTGAGGGATCGTGTCGATATTTGGTTGGAGCTGGAACAGACGTAGAAGAAACTTACCATGGT
GTCATGCAGAATATGATTAAACGCGAACAGCCGAGTCATCTAAAAACGAAACGCATCCGATACGCCTGTTGTGCTCCCCGT
TATTTGCAAAGTTTGTTGTTGTTAAGACAGAGTTCGATAACTGGTGATATCGTCATGGTTACATTGGACAACGCTATTGTAG
AAACATGTTGGTGTGTGTACATTGGATCAGGTGAAGGTAGAGGATCATTATTGACATGCGGAGTTGAAGAAAACCCAGGTC
CACTGCAGATGAGTAAAGGAGAAGAACTTTTCACTGGAGTTGTCCCAATTCTTGTTGAATTAGATGGTGATGTTAATGGGCA
CAAATTTTCTGTCAGTGGAGAGGGTGAAGGTGATGCAACATACGGAAAACTTACCCTTAAATTTATTTGCACTACTGGAAAA
CTACCTGTTCCATGGCCAACACTTGTCACTACTTTCTGTTATGGTGTTCAATGCTTTTCAAGATACCCAGATCATATGAAAC
GGCATGACTTTTTCAAGAGTGCCATGCCCGAAGGTTATGTACAGGAAAGAACTATATTTTTCAAAGATGACGGGAACTACA
AGACACGTGCTGAAGTCAAGTTTGAAGGTGATACCCTTGTTAATAGAATCGAGTTAAAAGGTATTGATTTTAAAGAAGATGG
AAACATTCTTGGACACAAATTGGAATACAACTATAACTCACACAATGTATACATCATGGCAGACAAACAAAAGAATGGAATC
AAAGTTAACTTCAAAATTAGACACAACATTGAAGATGGAAGCGTTCAACTAGCAGACCATTATCAACAAAATACTCCAATTG
GCGATGGCCCTGTCCTTTTACCAGACAACCATTACCTGTCCACACAATCTGCCCTTTCGAAAGATCCCAACGAAAAGAGAG
ACCACATGGTCCTTCTTGAGTTTGTAACAGCTGCTGGGATTACACATGGCATGGATGAACTATACAAATAGGAGCTCACAC
TTTTCGTTTTAATTTATAATTTTAAGTTTTGCTTGTATATCATGAATATAGGGAAGGCACAGAAGTAACGTAAATGATAGTATT
ATTCTTTATTCCAATACTTAATAAAGCACAAGGCCCAAAATTTGCTATGGGAAACATTTACGCGCTTAAAACATAAAAATCAA
AAGCCTCCGTTTCCAATTTTTTGCGTTTTTTGAAATGTCATTTTCCTGTACTTGAGAAACCGAAAGATACTATATAAAAAAAT
GGGTTCAAACCGGTCGCCAGTGGAAATCCTATCAACGGAAATTATACNNATTCATTGCTTCAACTAATAAGTTCGTATGTCG
AATAATAAAATGATTTCAGACCCAAGTTACGATTAACGGTTCTTGAATTATCCTATTGTCGCTAGAAATTGGTGCAACGACAA
ACCTCTAATTCGATGGCTCGAGAATTCGAGTTATAGCACCGATTTCGCTTCACACATACTGACGGGTTTTGATGTTGCACTG
TGAAATGCAAAGCTCGTCCCATCACCTTTAACCCTGGCTGTATTAGGGGCAGCGCTGTTGAGAAGAAAAAGTCCTGGGGA
CGAGGTTGAAGTCTTGTCGAGATAACTTAGTTCATACTTTGAACTGGGTTGTACTACAGACACATCTCTTTGTGGACCTCTG
TCCGAACAATTGATTCATATGTTTAAAACCTACTGAATACCATTTTCTTACTATTTAAACTTCATTATTTTTGGGAAAGGCCAT
TGCTTAGATGCAACCTCGTCCATAGGGTTTTTGCCTTCTTGACATCAAGTAAGCGCTAGCGGCTNNNNNNNNNNNNNNNN
NNCCTGGGGACGAGGATGGCTTAAATGGGGATTCATCCAACGGCCCACACACAGTGTACCATCTCCCCATTTGCCCTCAC
ATGGTTAGGGGTAACCCGGGGCTATGGTAACATTCTCTCGCCCATATTGTGAATAGCTGTCAAGGGAAACCGAACCCCGG

TCTGCCGCACAAAGTGCGCGAGTTATAACCAAAGCTACGGCGCCAGTTTTAAGGGCAATGTCCGTTTATGCCGAATGTGA
CGCCTCGGTTGCCCACGATGGCTCCTATGGCTCAC

5’RFamide/3’β-Tubulin, Gls, T2A, GFP codon optimized

Genomic sequences:

- ***Gls* wild type sequence**

TACCCAGCATAGCTGCAGAATTTTTGAAATGATTCCAGCTTACAATAATAACTTCATATCAACGGTTCTTGACTCAGCTATAA
AACAGGACTCATGTCTAAAGCTTGTTTTTTTATCAATTCTATAACTATTCTATAAAAGAAACACTAGGATTGATATCAAGGAG
GCATTATAATACAGATTGTGAAGAAGGACTTTAAATACCTCATTGTTGCAGAATAATTGATCTTTGACAAATCAGATCACAAT
ACAGTAAACAGTTGCAAGATGTTTACGCTATCCCTTAAAACAGCGTCGAAAATATCAAGTACTTTGTGATTGAAGCATCAAA
TAAACTGTTGATTGAAAAACAATTACCAACGCTTTATAAAAAAGATTAGGAGATTATATTAAATGGCCCGCTATTACACAGTT
CCAGACACAGAATCTGGAAAATTAATAAACATTTTTTCTTTCAAACCCTTGTAATTTGAGATTATTACAAACGAATAAAGATAT
AGTTAAAATTATTTAGTTCTAAATTTTGGTCATCACTGCTATGACAAGTTGAATTACTATAACTAAAAGTTGGAACACTACAAC
CACAAGATAAATTACTACAAGCAGAAGTTAGATTACTGCAACTATAAGTTGAATCAGTACAACCACCAGTTGAGTCACTGCA
ACCACCAGTTGAATTACTATAACTAGAAGTTGAAACACTACAACCACAAGAAAAAATACTACAATCAGAAGTTAGATTACTG
CAACTATAAGTTGAATCAGTACAACCACCAGTTGAACCACTGCAACCACAAGTTGAATTACTATAACTAGAAGTTGAAACAC
TACAACCACAAGATAAATTACTACAATCAGAAGTTGAATTACTGCAACTATAAGTTGAATCAGTACAATCACAAGTAGGATCA
CAATAACTACAAGTTGAATCAGTACAAAACAAGTTTAATCACTGAAAGTTAAACAACTACATTCGAAACAGTTATGTATTTGA
AACAGAAGCATTATATGCAGAATTTAAAAGTCCTTGTATAATCAATTCATGTAGACAAAAAAAATTGAGATTACCGCTTTTTTA
AAACAGGAATTTACAACTGGAAATAAAGGAGGACGTATGTTTTATTCATACATCGTCTTGTATTTAGTATAAGACTGAATGCC
TGATCGTTTTGTCTTTACAAACTATGTATAGGTAGTTTACTACATATGTCAAAGAAACTTCTAGACCACCATAAACTTCACATT
GTTATTGTTTCTTTCGTACAAAATGAAATATAAATGTGTTGTAAATAATCAATTACATCGTTTTTAGACCATAAAAATTATTGTC
GCCCTTTGTGTAGTCAAAACATTGTGCGCCCCCCTGAAATTATTTCGCTTCATACGGACCTGTTTGGCTCAAATTTATCTTC
AACAAATAACTTTTTTAGATTCAAGACATAACAGCACATCTGGCCGATTCCGTTATCAATTCAACCTAAGCTCGATTGAAGCA
GACTTGATTATCCGAAGGGTTGAGTTGCGTGTATTTAAAACAAAATCAACACGTCGAGAAAATGTATTTTACAGAACAGATA
TAGTCCTTGACGGTCCATCGTACCGAGACATTCGCTCAGTAATGGTCAGCTCAACAGGTTATGGTTGGTTGTCGTTTGAAA
TTACCGATGTTGCAAGTCGATTAATTCAATATGGGGATAAAATCGTGGATATTGATATTACATTGCAACCAATTCGTAACAAA
CTTCCAACATTTAAATTATTAACATCAACAAATACGTCTACAAGAGCCTTATTCATGGTGTATTCCGACACAAACGTCAAATC
ATCTAAGAAGGCGTCAAGATTGTACGGCACAGGTAAATACGCAGGTGTGCAAGAAAAAAATCGGCATCGGCGCGATGCTA
AAAATTCCCGTCAAGCTTGCCGATTGGCTAATCTGACAATTACATTCTCAGAACACACTGCATTTGGTGATGTCATGTTGCC
ACGCAAGGTAAACATTCGCAGATGTGAGGGATCGTGTCGATATTTGGTTGGAGCTGGAACAGACGTAGAAGAAACTTACC
ATGGTGTCATGCAGAATATGATTAAACGCGAACAGCCGAGTCATCTAAAAACGAAACGCATCCGATACGCCTGTTGTGCTC
CCCGTTATTTGCAAAGTTTGTTGTTGTTAAGACAGAGTTCGATAACTGGTGATATCGTCATGGTTACATTGGACAACGCTAT
TGTAGAAACATGTTGGTGTGTGTAACTTATCACGTTAAGCTGTTTTACAATGGTGGAGGGCCAAAGTAGTTATTTGCTATTC
CGACGCGTTTTGCTGTCGTTGGCAAAGAGAAACACATTCTTTAGTTAATCAAAACTTTGAAAAATGTGACAAGACCCTAACA
TACTTCTCATTTCTATAAACAATGCTTAAATTTAAACAATTGCTGACTGGTTCGCTTACAGCGAACAAATAAAAAATACCATTA
AATGGTATAAAATGAAAACAAACATTTCGCCATATGAACACAGACTTGTAAACCTTGCTTAACCAATTTTTCAAATATTTTTTG
TAGTTAAAAAATGCTTGGGCTGAAGTGCAGAAATCGTTTACAATCAACAGAAGGAGTGTCCTTAGTAAAAACATTGATTATA
TTGAACGGCAACGAGAGTCT

Fwd/rev; Intron; Exon2 ; sgRNA, Deletion

- ***Gls* mutant sequence**

TACCCAGCATAGCTGCAGAATTTTTGAAATGATTCCAGCTTACAATAATAACTTCATATCAACGGTTCTTGACTCAGCTATAA
AACAGGACTCATGTCTAAAGCTTGTTTTTTTATCAATTCTATAACTATTCTATAAAAGAAACACTAGGATTGATATCAAGGAG
GCATTATAATACAGATTGTGAAGAAGGACTTTAAATACCTCATTGTTGCAGAATAATTGATCTTTGACAAATCAGATCACAAT
ACAGTAAACAGTTGCAAGATGTTTACGCTATCCCTTAAAACAGCGTCGAAAATATCAAGTACTTTGTGATTGAAGCATCAAA
TAAACTGTTGATTGAAAAACAATTACCAACGCTTTATAAAAAAGATTAGGAGATTATATTAAATGGCCCGCTATTACACAGTT
CCAGACACAGAATCTGGAAAATTAATAAACATTTTTTCTTTCAAACCCTTGTAATTTGAGATTATTACAAACGAATAAAGATAT
AGTTAAAATTATTTAGTTCTAAATTTTGGTCATCACTGCTATGACAAGTTGAATTACTATAACTAAAAGTTGGAACACTACAAC
CACAAGATAAATTACTACAAGCAGAAGTTAGATTACTGCAACTATAAGTTGAATCAGTACAACCACCAGTTGAGTCACTGCA
ACCACCAGTTGAATTACTATAACTAGAAGTTGAAACACTACAACCACAAGAAAAAATACTACAATCAGAAGTTAGATTACTG
CAACTATAAGTTGAATCAGTACAACCACCAGTTGAACCACTGCAACCACAAGTTGAATTACTATAACTAGAAGTTGAAACAC
TACAACCACAAGATAAATTACTACAATCAGAAGTTGAATTACTGCAACTATAAGTTGAATCAGTACAATCACAAGTAGGATCA
CAATAACTACAAGTTGAATCAGTACAAAACAAGTTTAATCACTGAAAGTTAAACAACTACATTCGAAACAGTTATGTATTTGA
AACAGAAGCANTNTATGCAGAANTTAAAAGTCCTTGTATAATCAATTCATGTAGACAAAAAAAATTGAGATTACCGCTTTTTT
AAAACAGGAATTTACAACTGGAAATAAAGGAGGACGTATGTTTTATTCATACATCGTCTTGTATTTAGTATAAGACTGAATGC
CTGATCGTTTTGTCTTTACAAACTATGTATAGGTAGTTTACTACATATGTCAAAGAAACTTCTAGACCACCATAAACTTCACA
TTGTTATTGTTTCTTTCGTACAAAATGAAATATAAATGTGTTGTAAATAATCAATTACATCGTTTTTAGACCATAAAAATTATTG
TCGCCCTTTGTGTAGTCAAAACATTGTGCGCCCCCCTGAAATTATTTCGCTTCATACGGACCTGTTTGGCTCAAATTTATCT
TCAACAAATAACTTTTTTAGATTCAAGACATAACAGCACATCTGGCCGATTCCGTTATCAATTCAACCTAAGCTCGATTGAAG
CAGACTTGATTATCCGAAGGGTTGAGTTGCGTGTATTTAAAACAAAATCAACACGTCGAGAAAATGTATTTTACAGAACAGA
TATAGTCCTTGACGGTCCATCGTACCGAGACATTCGCTCAGTAATGGTCAGCTCAACAGGTTATGTTGGTGTGTGTAACTT
ATCACGTTAAGCTGTTTTACAATGGTGGAGGGCCAAAGTAGTTATTTGCTATTCCGACGCGTTTTGCTGTCGTTGGCAAAG
AGAAACACATTCTTTAGTTAATCAAAACTTTGAAAAATGTGACAAGACCCTAACATACTTCTCATTTCTATAAACAATGCTTAA
ATTTAAACAATTGCTGACTGGTTCGCTTACAGCGAACAAATAAAAAATACCATTAAATGGTATAAAATGAAAACAAACATTTC
GCCATATGAACACAGACTTGTAAACCTTGCTTAACCAATTTTTCAAATATTTTTTGTAGTTAAAAAATGCTTGGGCTGAAGTG
CAGAAATCGTTTACAATCAACAGAAGGAGTGTCCTTAGTAAAAACATTGATTATATTGAACGGCAACGAGAGTCT

- **Podocoryna carnea sequences**

>Podocoryna TRINITY_DN90629_c0_g1_i1 len=1471
MQLKFSSTHSVLFYVAMVAELVALSNASSKTKSNTLKLQVPQFMMDLYNVVADKNGYSRNGAPDLGKTITCISAHDSRYLNKSH
AFHYRFNLSSIEKNLNIKRVELRVFKTKSKRRENIFYRTDIMLDGDSYRDTRSIMVSSTGYGWLSFDVTNIARQLIKYDDKAVNIDIT
LRPIRNQLPAFRLLTSMNTSTRALFIVYSDGPGSLSQHTSDKGTLLPYDTSKNHNRQRRNARKTRHACRLLNLTINFDKHDEFKN
VIIPSVAHIQRCAGSCRLPSGFGSEAEETYHAIMQNMIKRVESSKKKRMKYACCAPRELHSLLMLTKCSSSDDIKMRTLEDAVVA
SCWCV

>Podocoryna_TRINITY_DN13881_c0_g2_i3 len=2420 path=[0:0-422 2:423-457 3:458-950 4:951-999 6:1000-1033 7:1034-1096 9:1097-1120 10:1121-1159 12:1160-2419]: 2311 to 1157: Frame -2 385 aa
MEYMVFVFYVLFVCNIEYTTATPVSYIETNKVVNTDTEKFVNHFNEQHRIDRENDVTVEEEEKEAIEEEKLRKAHVPKYMRDLYK
VLNKYADEGLLHHGNIVRSFSDQGNAKSERNLKKYQFDISKASKDTETLTKAELRLFKLPNRKKGSLSSYIQIDVEDVYSGKIVSS
KVSSIHSNGWQVFPLTGIVKIWLADQSKNKGVRVTARSLYGDNNGITFATKDVISKEPILVVYTKDHNELTLASLLKFKVNTTALFT
KETSDYENKHKRERRDSGYDLCHLEKLSVPLDEIGWDKNFASPTKFKINQCKGVCRHDSTDSTKQTNHALIQALYAAVTGGRKV
SYPCCAPAKYASGTALVVDRLPTGGTVELVYLQKMTVTRCECL

Multiple sequences alignments:

- **TGFβ alignment**

>AID65996Hydra
------------------------------MKVTLLSIIRLTVYVFV--
-------NFVNGIPTKERNIIQNTLLDLFGKHNRPRPDVFSHLGRNETSAKK-YMLDLYEYSVNTER---------------
QGMNNFTTNSQNISNIVDDADTVVSFLN----NAYVPRPNTTDESGEMFFDVSS-NYVVE-KVLATALQVYLDI----TYKTVIAEK-
LIISVYKI---VVP-----------KKKYVLLASKLINASVSQ-WHEFNVLEASLSWIEFSETNNGILLVCQNLQKVNIPIE----------------------------
---SCGIVDFKGREEFRPFLVSFYQSGKEEEFPAKQIRNTESTLKL------------QERLRRSMQDSIFIEAAEQLS----
FVRNKTRSNGSENNLGSRCDKHPLYIGFK-DLGWSDWIIAPDGYRANYCGGDCSFPLDNN-
ANATNHAIIQTLVHMMYPEIIPKPCCAPNKLNTLQVLFLDE-RNNVVMKRYSNMIVQHCGCQ
>XP_002165935_2Hydra
------------------------------MKVTLLNIMVLIIHTH--
------SFVNGVTTIERNVIQNTLLDLFGKHNRPSPEVFSHIGRNATSAKK-YMLDLYEYSVNTER---------------HGMNNFT-
SPKNISNIVDDADTVVSFLN----NAYIPRPNTSDEAGEMFFDVSS-NDVVE-KVLATALQVYLDT----TYKTVTMEK-LRISIYKI---VVP-
----------KKKYKLLTSKLINASVSQ-WHEFNVLEASLSWIEFSETNNGILLVCENQQKETIPIE-------------------------------
DCGIVDFKGREQFRPFLVSFYQSGKEKEFPAIKIRDIESTSNL------------HERLRRSMQESIFVEVAGQFA----
AAQKKMRLNISQNIHGSRCDKHSLYIRFK-DIGW-NLIIAPEGYEANYCGGECSFPLDNN-
ASATNHATIQTLVHMMFPEIIPKPCCAPNKLKRLKVLFEDE-RHNVVLSHFPNMIVQHCGCQ
>XP_002165531_2Hydra
------------------------------MKKTVLFVLVSLISST--
-------SFLNGIPTKERNVIQNTLLDLFGKHNRPRHNLFSDVGKIVTSATK-YMLNLYQYSLGSEL---------------
QNINNPTINLKNISKKVNFADTVVSLLN----NGYVPRPNTTDESGEMYFDVSS-NYDIE-KIIETNLQIFLDV----TYKTVPLNK-
FRLEVFKI---VVP-----------KKKYILLDSKIIMTSVSQ-WHEFNVLEALLSWVKYSETNNGILIVCKSLQQEAILVE----------------------------
----
NCGVVGFKGSKDYQPFLVSFYQSSKNEEFFALKAPDFDSTSRVQYFLDNKFKTSTPKRFTRGIEDNDFLKAAATLAEKNNFVQS
KLISKRSKNNKISDCGKHLLYVSFK-DIGWSDWIIAPDGYITSYCEGDCSFPLESN-
LNATNHAILQALVHTIFPKKIPKPCCAPNKLNAMSILFFDD-RNNVVMQEIPNMIVHQCGCQ
>HyS0021.105
--------------------------------MRLLQMCIYIYGVFE--
----MIQHEVMCIPQGTRSEIEKNVLEIFGMDNRPRPDLIQSFSRDVTSAKK-YMIGLYETVQEKAV-----------
SQNTTESTMNTTVTGNSTDLLHKADTIISFVN----NGLVPRPNASDEDGAMFFDVES-NLVIE-DTLASEVRIFLNV----
SKKKLADNV-LRVTVYKI---IIP-----------KTRYLVLASRIVNATESQ-WHEFDVLKASISWKEDPSQNNGILVVCETLTNQVKAFK------
-------------------------ECGLIDLRSDDENRPFLVSFYHSGDEDEILAEQLPEDGDTDYKTQA---------RERRRRNLDH-LFPRGSMSG---
---------SFRNLKRHNSTCGRHPLYIAFR-DLGWNDWIIAPEGYKAAYCGGECKFPLHEN-
MNASNHAIIQTLVHMMMPEKIPEPCCAPVRLEPLKVLYLDR-SNNVVMKTYSDMIVNDCGCQ
>HyS0021.106
--------------------------------MQLLHIAYVLVALAC--
----------NYEAVPLSSNLQQTMDVVFGKNNIPNNSKFGKLGRDISSAKR-YMIGLYQYMNKTEC------------
SMGQCFNKSVIASTDSENMVHDADTIVSFVH----HPYIPRPNISDEEGAVFFNVES-NFNNE-DTIKSEIRIYLNT----SQKTIEGAT-
LRLSVFEI---VIP-----------KTKYTMLASKLVNATESS-WHEFDVLRATSTWNDEPSQNNGLLIVCETLERKKTPLK----------------------
--------ECGLIDVKGEDDYRPFLVSFYQSDEEEQEEYLAEQLPENENST------------NTRTRRSLND------------
LFKQASKNVPGFYEREANRFTCRKIPLYVTFE-ALKWTDWIIAPEGYKSYYCGGECKFPLHHN-
SNATNHAIIQTLVHSLSPKRAHPPCCAPSRLEPLTVLYVNN-DENVVIKKFSNMIVNDCGCQ
>human_BMP6_P22004
-------------------------MPGLGRRAQWLCWWWGLLCSCC--
LLGDGGSPGRTEQPPPSPQSSSGFLYRR----LKTQEKREMQKEILSVLGLPHRPRGLQQPQPPALRQSAPL-
FMLDLYNALSADNDAAHPLNRKSLLAPGSGSGGASPLTSAQDSAFLNDADMVMSFVN--LVEYDKEFSPRQRHHKEFKFNLSQ-
IPEGE-VVTAAEFRIYKDC----VMGSFKNQT-FLISIYQV---LQEHQHR-------DSDLFLLDTRVVWASEEG-
WLEFDITATSNLWVVTPQHNMGLQLSVVTRDGVHVHPR------------------------------
AAGLVGRDGPYDKQPFMVAFFKVSEVHVRTTRSASSRRR----------------QQSRNRSTQS-------------
QDVARVSSASDYNSSELKTACRKHELYVSFQ-DLGWQDWIIAPKGYAANYCDGECSFPLNAH-
MNATNHAIVQTLVHLMNPEYVPKPCCAPTKLNAISVLYFDD-NSNVILKKYRNMVVRACGCH
>XP_032219054.1 bone morphogenetic protein 7 [Nematostella vectensis]

---------------------------MRRIRDLLLVCFLILKASQ---
--SEQNSSLAS----RNDDTPPTLKQELLNLMGLPRKPKLSLVTRMYGKRFSGPR-YMIALYNSLSQNQT---------------
TDTTGFCCNGTLADSRAVGADTIMSYLN----HEGGRRPKISHMHATYKFKLLT--PEGE-RVTATEFRLYKED-TRGDPSNWRNAT-
YVVRLYQV---IYP-----------AQVLDLIDQRVLRSWEKG-WQEFDISAAGRVWSESPEKNYGLELSVVNFNNQELSPH-------------------
------------LAGFVGFHGLKEKRPFIVSFFKQDGEKKYTHEIHAHDIRQHR-------------ATRVSRSLPN------------------
AQVGGVDPRASNKKVCQRQALHVSFR-KLRWQDWVIAPEGYSAFYCSGECSFPLNAN-
MNATNHAIVQTLVHLMNPKTVPKPCCAPTELSPISVLYFDQ-DNNVVLKKYNKMVVKACGCH
>Sea_urchin_DVR1_Z48313
-----------------MEYSRKTYLDLNIMAKYILILSLFFGPGL---
WDVFYSGDEDQLSLARERRAANYNPSPH----MSTWERNEIQQEILNILGLQHRPRPPSLRG------
GQAQFTEWSYYRTLNIDEQSNAIYNSPDSSGIGSVMSGTVFNYTRNEVQAVSQADTIMSLPV------
HYKDAAIEDTEHRYRFDIGR-IPQGE-TVTSAELRVFRDA-------GRQGRSLYRIDVLLL---RERGSDG-------
SRSPVYLDSTIVGAGDHG-WLVFDMTSATSTWRSYPGANVGLQLRVESLQGLNIDPT--------------------------------
DAGVVGVGNNEGREPFMVVFFQRNEEVIATNSHLRRNRRA---------------ATRQKKGGKR---------
PRKPDTDNDIASRDSASSLNSDWQCKRKNLFVNFE-DLDWQEWIIAPLGYVAFYCQGECAFPLNGH-
ANATNHAIVQTLVHHMSPSHVPQPCCAPTKLSPITVLYYDD-SRNVVLKKYKNMVVRACGCL
>Drosophila_Gbb_M77012
-------------MSGLRNTSEAVAVLASLGLGMVLLMFVATTPPA--
-----VEATQSGIYIDNGKDQTIMHRV----LSEDDKLDVSYEILEFLGIAERPTHLSSHQLSLRK-SAPK-
FLLDVYHRITAEEGERGHRSRRSADLEEDEGEQQKNFITDLDKRAIDESDIIMTFLN---KRHHNVDELRHEHGRRLWFDVSN-
VPNDN-YLVMAELRIYQNA--NEGKWLTANRE-FTITVYAI---GTGTLG--------QHTMEPLSSVNTTGDYVG-
WLELNVTEGLHEWLVKSKDNHGIYIGAHAVNRPDREVK------------------------------
LDDIGLIHRKVDDEFQPFMIGFFRGPELIKATAHSS----------------------HHRSKRSASH---------
PRKRKKSVSPNNVPLLEPMESTRSCQMQTLYIDFK-DLGWHDWIIAPEGYGAFYCSGECNFPLNAH-
MNATNHAIVQTLVHLLEPKKVPKPCCAPTRLGALPVLYHLN-DENVNLKKYRNMIVKSCGCH
>Crassostrea_gigas_mGDF1_CAA10268

--MAGCGDQNCAHFYFNVSS-IPVEE-
SLTGAELRLFVDQNNETNPVGNRKQFRHKIEIHEV---LQPETANS------EAITRLLDVRHVGGKNSS-
WESFDIHPAVLKWKKNPTLNHGLKVRVLSFKNKPSTDS-------------------------VKHVRLRRDVESVEEA-
WHERPLLVTFTDDNRGSRTKRATSDKKVKKNKKNR----------KNKNKRRKNR-----------------
KKNRKNKTKRKKYNNQCRRKELNVDFK-AVGWNDWIFAPPGYNAYYCDGSCHWPYDDH-
MNVTNHAIVQDLVNSIDPRAAPKPCCVPTELSSLSLLYTDE-HGAVVLKVYQDMVVEGCGCR
>human_BMP2_P12643
-------------------------MVAGTRCLLALLLPQVLLGGA--
AGLVPELGRRKFAAASSGRPSSQ----PSDEVLSEFELRLLSMFGLKQRPTPSRDA-------VVPP-YMLDLYRRHSGQPG--------------
------SPAPDHRLERAASRANTVRSFHH--EESLEELPETSGKTTRRFFFNLSS-IPTEE-FITSAELQVFREQ--
MQDALGNNSSFHHRINIYEI---IKPATANSK-----FPVTRLLDTRLVNQNASR-
WESFDVTPAVMRWTAQGHANHGFVVEVAHLEEKQGVSK--------------------------
RHVRISRSLHQDEHSWSQIRPLLVTFGHDGKGHPL---------------------------HKREKRQAKH-------------------------
KQRKRLKSSCKRHPLYVDFS-DVGWNDWIVAPPGYHAFYCHGECPFPLADH-LNSTNHAIVQTLVNSVN-
SKIPKACCVPTELSAISMLYLDE-NEKVVLKNYQDMVVEGCGCR
>human_BMP4_P12644
-------------------------MIPGNRMLMVVLLCQVLLGGA--
SHASLIPETGKKKVAEIQGHAGGRR--SGQSHELLRDFEATLLQMFGLRRRPQPSKSA-------VIPD-YMRDLYRLQSGEEE----------
------EEQIHSTGLEYPERPASRANTVRSFHH--EEHLENIPGTSENSAFRFLFNLSS-IPENE-VISSAELRLFREQ--
VDQGPDWERGF-HRINIYEV---MKPPAEVVP-----HLITRLLDTRLVHHNVTR-
WETFDVSPAVLRWTREKQPNYGLAIEVTHLHQTRTHQG--------------------------
QHVRISRSLPQGSGNWAQLRPLLVTFGHDGRGHALTR-------------------------RRRAKRSPKH----------------------
HSQRARKKNKNCRRHSLYVDFS-DVGWNDWIVAPPGYQAFYCHGDCPFPLADH-LNSTNHAIVQTLVNSVN-
SSIPKACCVPTELSAISMLYLDE-YDKVVLKNYQEMVVEGCGCR
>Halocynthia_BMPb_BAA31132
-------------------------MFTNNKYELVFIMVMVLRASA--
MIPEIGRNNLIEQAAKYGTNT----REDEIVNEFEKKLLNMFGLTRRPTPNEDM-------PIPR-IMQHLYKAHMGDTS----------
DGPHDLYPGWEPGFDLPSDDIASTVNTARSFHH---KDEFERIEGLPDHHIRLLFNIST-LPPNE-
NLKYAELLLHREEIKQHVSLPDEDRM-QRINIYEV---IKFPDDDVTTEEQNEPITRLVATRLVDTSNTT-WERFDVSAETQKWLKDK-
-NHGLVVEIVRDGDESTPAP----------------------DVTSHVRLRRDVRNENEPWHHKRPLLLTYTHDGKETNLSR-------------------------
RSRKKRKAH------------------------RKKERKPRRKSCQRQDLYVDFS-DVNWDDWIVAPHGYHAFYCNGECPFPLAEY-
MNATNHAIVQTLVNSVDPSLTPKPCCVPTELSPIAMLYVDE-CELVVLKTYQQMAVEGCGCR
>XP_032235826.1 bone morphogenetic protein 2 isoform X1 [Nematostella vectensis]
ISDQKDQIIVYKRTLYILTSITEVNTVPILSGKMLLLRCFLLLSMY---
-------------FPVHGLPAESSSQRAKHEV----RNSADFQMMEQRFLDTMGMRSRPRPKRGT-------RIPQ-YMLDLYNSHKAHPD-------
-------------WISTQFRFGDKWIGANTIRAFHH---TDNDGIDASENSNLTRIIFDLST-LPMYE-TVTSAELRLSTRS--GDLKQPSNSTQ-
LWIRVYQV---LLPGMGDT------PALRRLLDSREVDARVAG-WESFNVELAIQHWVKNPEQNYGLDIQVTTKEGHSIKDA--------------
---------------VRTREHHAEEDWHEERPLIVTYNHDELHHH----------------------------HTRRKRSLR------------------------
SGGAKRRRPQYCQRHPLYVDFT-DVGWNDWIVAPPGYHAFYCTGVCPYPIAKH-LNATNHAIVQTIMNTVD-
SNVPNACCIPTTLNPISILSLNE-FDKVVLKNYKDMVIEGCGCR
>Drosophila_Dpp_P07713
MRAWLLLLAVLATFQTIVRVASTEDISQRFIAAIAPVAAHIPLASASGSGSGRSGSRSVGASTSTALAKAFNPFSEPASFSDSDKS
HRSKTNKKPSKSDANRQFNEVHKPRTDQLENSKNKSKQLVNKPNHNKMAVPANAKAIIAEQGPSTYSKEALIKDKLK----
PDPSTLVEIEKSLLSLFNMKRPPKIDRSKI------IIPE-PMKKLYAEIMGHEL---------------------DSVNIPKPGLLTKSANTVRSFTH---
KDSKIDDRFPHHHRFRLHFDVKS-IPADE-KLKAAELQLTRDAQVVASRSSANRTR-YQVLVYDI---TRVGVRGQR-----
EPSYLLLDTKTVRLNSTD-TVSLDVQPAVDRWLASPQRNYGLLVEVRTVRSLKPAPH--------------------------
HHVRLRRSADEAHERWQHKQPLLFTYTDDGRHKARSIRDVSGGEGGGK-------------GGRNKRQPR------------------------

RPTRRKNHDDTCRRHSLYVDFS-DVGWDDWIVAPLGYDAYYCHGKCPFPLADH-
FNSTNHAVVQTLVNNMNPGKVPKACCVPTQLDSVAMLYLND-QSTVVLKNYQEMTVVGCGCR
>Halocynthia_BMPa_Q94580
EVNADNFNCDCPLTNYRKNLSKTSVSRYDFIKKLTFLYALIIATND--
-------------LIITPHLGVTSYVLSQTDTSNRR----LTSREKFEKQREMLSILGLSHRPRPAGDPARGLQHDSAPL-
YMLGLYNTVSGFSDFTNEVGHIEITGSLNSPNVQRLQTNSVEDKLLGDADMVMTLINEDDEAHDVAMRSLLKRHRSFYFEINQ-
LPEND-YLITSKFRLYKDI----TDHSLGNIT-LRVNVYQA---MKDEQN--------NYSLYLLGSRSVLGQQEG-
WLVFDITSATRDWVEVQESNLGIRVAVETREGRSVNLN-------------------------------
KAGIVGKRGDASKQAFLVAFLSEANDRLPSRMWRRIRRNAATDRE----------
ATRRRRSEQSTEMEEEKRLRRMRRRLRKKSQRRKKKLKETGQACHREELYVSFQ-
DVNWEDWIIAPSGYMAYRCSGECDFPLSAN-MNATNHAIVQTLVHLLKSKLFPEPCCTPQDLDSISVLYYDD-
HRNVVYRKYRNMVVLSCACY
>Drosophila_Screw_P54631
------------------------------MLNVFFLTSLFYAASA---
TTYVTTNNHIEMPIYQKRPLSEQMEMIDILDLGDRPRRQAEPNLHN---SASK-FLLEVYNEISEDQE-----
PKEVLHQRHKRSLDDDILISNEDRQEIASCNSILTFSS-----RLKPEQLDNELDMHITFNTND-VPVDL-SLVQAMLRIYKQP-----
SLVDRRAN-FTVSVYRK---LDNRQ---------DFSYRILGSVNTTSSQRG-WLEFNLTDTLRYWLHNKGLQRRNELRISIGDSQLSTFA-
------------------------------AGLVTPQASRTSLEPFIVGYFNGPELLVKIQ------------------------KLRFKRDLEK---------
RRAGGGSPPPPPPPPVDLYRPPQSCERLNFTVDFK-ELHMHNWVIAPKKFEAYFCGGGCNFPLGTK-
MNATNHAIVQTLMHLKQ-PHLPKPCCVPTVLGAITILRYLN-EDIIDLTKYQKAVAKECGCH
>XP_001623157.1 growth/differentiation factor 6-A [Nematostella vectensis]
-------------------------MQAWFRAFSVICFTRIFIQSY--
KPQNLDSKWEALHRPL----LRKRALELTQKHMLNVLGLSEMPRVHRRRVR-------PHSFMLELYRTLSSKMD-----------------------
RNKARKSRHAFVNTVRGVVD----QESLDKSVLELKDQLYVFNTSN-IPYSE-KLVSAELRLLRIPTNTDNEVVIEHGTAYRAGIYAK--
-NTRSSFYG------SAGLEMLDSFVITDTDKR-WFVMSVTKAVQRLESRKNVCFLILKVMSLTSGKLIAP------------------------------
VRMGFSKEFRVHDQRALLVLFADDGKMKSEEGTRDGDLGRPIGGQNHVRDYYETQETTRAKRSVNR-----
NIPKAVASAIDTSRKRRKKSRRQREKRKCRRKRMYVDFR-LLGWSDWIIAPQGYDAYLCEGECKYPIDNY-
LRPTNHATVQTIVNSLDPSIAPKACCTPNELSPISILYTEDGSNNVVYKNYKDMVVERCGCS
>Cassostrea_gigas_mGDF3_CAD67715

---NMYIGVD-------------------------------
RKVSVYEV---TDD-----------NVGYHVIDTQHVYGRDSG-WETFDVTSAVRRWVTKPSSSQILEIRIESVFHSVTDGD--------------------
-------------LDFLTEPQHKNEPLLVVFSTDKQKLQLHKTERHELITREDSIQSP--------LSGSKNSNNS--------
LNETNTIGHKIHSRVKRRGRRSGGLCRRRPMTVNFA-DIQWDSWILAPSSYEAYECVGKCHFPVNER-LSPSL----------
TMPKEYPESCCVPTKLDSISILYYDE-YGVLTYKKYDGMVVTECGCR
>Gallus_Dorsalin_P34822
---------------------MHYFGVLAALSVFNIIACLTRGKPL---
ENWKKLPVMEESDAFFHDPGEVEHDTHF---DFKSFLENMKTDLLRSLNLSRVPSQVKTKE------EPPQ-FMIDLYNRYTADKS------
------------------------SIPASNIVRSFST-EDVVSLISPEEHSFQKHILLFNIS--IPRYE-EVTRAELRIFISC--HKEVGSPSRLE-
GNMVIYDV---LDGDHWENK-----ESTKSLLVSH--SIQDCG-WEMFEVSSAVKRWVKADKMKNKLEVVIESKDLSGFPCG-------------
-------------------KLDITVTHDTKNLPLLIVFSNDRSNTKETKVELREMIVHEQENKLGKNDSSSEEEQREEKAIAR-----------------
PRQHSSRSKRSIGANHCRRTSLHVNFK-EIGWDSWIIAPKDYEAFECKGGCFFPLTDN-
VTPTKHAIVQTLVHLQNPKKASKACCVPTKLDAISILYKDD-AGVPTLINYEGMKVAECGCR
>Mouse_BMP10_Q9R229
----------------------MGSLVLPLSAVFCLVAHSASGSPI--
MGLEQSPLEEDMPFFDDIFTEQDGI----DFNTLLQSMKNEFLKTLNLSDIPVQDTGRV------DPPE-YMLELYNKFATDRT-------------
-----------------SMPSANIIRSFKN--EDLFSQPVTFNGLRKYPLLFNVS--IPHHE-EVVMAELRLYTLV--QRDRMMYDGVD-RKITIFEV-
--LESADGSEE-----ERSMLVLVSTEIYGTNSE-WETFDVTDATRRWQKSGPSTHQLEIHIESRQNQAEDTG------------------------------
RGQLEIDMSAQNKHDPLLVVFSDDQSNDKEQKEELNELITHEQDDSDAFFSGPDEEALLQMRSNMI------------------
DDSSARIRRNAKGNYCKKTPLYIDFK-EIGWDSWIIAPPGYEAYECRGVCNYPLAEH-
LTPTKHAIIQALVHLKNSQKASKACCVPTKLDPISILYLDK--GVVTYKKYEGMAVSECGCR
>XP_032219916.1 bone morphogenetic protein 2 [Nematostella vectensis]
----------------MHSSSTQSLSACREMNHFKLLLFLTLLYPP---
-----------------YRTVAD----KRSLEIDDLKSDLLNLLEVKEKPAISRSRW------QVPR-YVIELYRKQAFIDG-------------------------
FTKNGHSTPGRTVRTFFR-------DEQDKKGPHKHLFTFNISA-MRPAE-KVEHAEIRIFKRR----SSLPNPRAL-YKVTVSRL---
TKPWSSVNK---RWKRKLVALDVQLVKCRKVGEWITFNVTSAVKFWSKYPSKNYGLWVSVRSYQAPPSDFK--------------------------
-----------IATGGRKDPILVEFGVDREKLQKAQMAEIQDE-----------------LQEKKNALNY-KVPNVDDLSRDNSKIIMASR-
TRSRRDVADNLCRRHRLFVKFQ-ELNWSDWILAPRGFSAYYCTGTCPEVIEQY-
FNPTNHAIIQNLLHHRYSKSVPAACCVPTRLHSISMLYFEL-DGSIVLKEYGEMVAASCGCR
>HyS0021.28
-------------------MKYRNITSKPILTILLFISIHYVTLTR--
TERRQMMPRLKEVSTDQLVNIYGNET----QKSELLEETKINLIKEFGLRKNMLTNLRR-------RMPHKFMRYLVEHFKDLRR----------
------------------TGKAAAANTIRGCTD----LGNQRRKLVPFRHISFKFDVSE-IPKDE-LTIFTELKVLMKV-----PKNLRHNS-
YTLTLYDVLPRRKDGAHK-------EPKLYRLRHKVVPGQSER-WITIDATESFLLAKSRNKKHVKLVLKAQPEGGLAIDPT---------------
----------------LIGLNNNHAEETDKALLVIYGGDLSDAPARRRRRTKRGVSSPKQSK---------KSQKRRQKNG--------------
KKKKKGKNRNKKNRKNRKACRRKNMEVDFH-QFGWTNWLFSPTKYNAYYCHGQCMYPIPSH-
LKPTNHAIIQSMMHDVERKIAPRPCCVPDEFEVLPFLLLDG-SDRVVFKLKEGLIVKSCSCR
>HyS0003.357
HCSAGDDKYHILKKDDWKQLLHGDCISLLPNSLFFTVEVTQDKTQAYSIDENVEDSATTSVDVITTKGDMSLLQSTTALTTVPSST
TSNSFVSKTRTLPSWMLQTESSQPKKKTKPAKKDTKERDSSPNEKVTDVLQNVCLQMEKTQETPSTNRGKFTTKSPV----
KNKRMLKMVEQRFMNFLGVKGAPTKSLNKEL-----KVPE-HLLKLYYKWSDDNF---------------------------KDYDKNNADTARVVYH---
SIEEDNDERRSSHKQKLHFNISD-IQRKE-KLHRAELLLFTER-----RNNKTKTK-NYICIYVN-------------
NTNNIIHDHPIIRKKLPDNRLG-YASFDVTATVNTWLNKSIENTVLNVKINEEDNHRVHLR-------------------------------
QRRDVSSYEWHKKRPLLVIYTRSENLRDSKTK-----------------------KERKRRNAYH-IKLKDYKKR------------

NETHQRVRRETCRLRYLRLDFK-TLNWNKWIVLPVGYNINYCSGVCPRPLAPH-
FNTTNHAIIQNSIISLDPSRASALCCVPTKLKEQAFLYLTS-ENKLILKNAVQMTAESCGCR
>NP_001296592_1Hydra
----------------------------MDIGSRSKWWVFLVLNFILETAF---
GLHENNRFYESSDKPI----RREEIKAVLEAKFLSILGLPFKPEKPKNKV------KIPN-HVWKLYTNGQIIYH--------------------------------------
DLDKKNADTIQFHYHEKPLINHVNEQKYKQNVEHLEFEIIF---KRVQATHRAELHVFSEP-----HNDLRERKPYQVTIYSN------------------
----NGTVPFIKKKIAGSRLG-FTTLEVTEIVQKNMNNT---PVLNIGITQNDRLHLRRR----------------------------------
RNTDDNEWNTKRPVLVIYTLCGEKTTKVEKS-----------------------HHRSKREAIE----------------------
KSPNSDNKQDLCHLHPFEFDLV-KVGWNEFVLYPSLYRLNFCAGKCPNPLSHH-
FNGTNHAVIQNNVLKIAKDKVPPLCCIPSELEAQTIIYIDH-ENKMIIKRPEKMIATACGCR
>Xenopus_Vg1_ AAA49727
-------------------------------MVWLRLWAFLHILAIV---
-----------------TLDPELKRREELFLRSLGFSSKPNPVSPP-------PVPS-ILWRIFNQRMGSSI------------------
QKKKPDLCFVEEFNVPGSVIRVFPDRFIIPYSDDIHPTQCLEKRLFFNISA-IEKEERVTMGSGIEVQ---------
PEHLLRKGIDLRLYRTLQITLKGMGR------SKTSRKLLVAQTFRLLHK--SLFFNLTEICQSWQ-DPLKNLGLVLEIFPKKESSWMST--
------------------------------ANDECKDIQTFLYTSLLTVTLNPLRCK----------------------------RPRRKRS--------------------------
YSKLPFTASNICKKRHLYVEFK-DVGWQNWVIAPQGYMANYCYGECPYPLTEI-
LNGSNHAILQTLVHSIEPEDIPLPCCVPTKMSPISMLFYDN-NDNVVLRHYENMAVDECGCR
>HyS0105.20
----------------------------------MCIIINKTIASFP--
---------DTQQQYVGHNELHTPAITQRDKTQERVT------SLSV-YLKRIYQ----------------------------------DPNYELNGANEITALLP----
IPFTRRPTYSDPEGVISFKIMESVENKR--IIKSALRVFVNS----EEAKTTNDR-FKLLVSQI---------------QRGACPKLIGSLSIYTTHSK-
WHEVDTTISALAWQQDPSENDGLLIECKTLKGKKKTLS----------------------------------
DCGINLNGLDEHLPLLTLFLYSDSGNQINANEMLPHGEFGKD-------------KKTPKRST----------------------------
SLANKRNARKLCRLKKFHVSFQEDLEWNEWIIAPTGYNFSICRGKCP-------RYNSNHADYDNVAIA--
YDQIPSPCCVPTKMSPLKVLFH-----NFKIIIYSNMIADECGCR
>BiomphalariaNODAL
----------------------MRASLTAQNVLWLLTIWSFIYVYQI---
WAQTFHPSLVLSSRAMSSQTSSGSMFSRAQTLEVKEDQVMFHFYRNGRSRKDYRKP--------STKFMVSLFNELQRVGN----------
---------------IRTRTSAANMTDVVRSFSG-------TSSQSKKTSRTITEFHIPY-LPPHE-RLKMAEIRFLREP----KAKPKLQQIKIRINIKKG-
-----------------SKALKKIVIRR-PQTQKE-HDVIDVTNFVSPWINSFHGNFSLAIRITGGQVSQTKNS---
DNLAQSLIVLYLQDGEFLANMYSSFTDSADTARR------------LSRRKRSSSS----
ILGGNLSSTIRNKRDRNLKKWNRAGKKVNCQLHNFEVDFN-TIGWGQWIIHPKKYNAQFCFGVCPSPVDSK-
YKPTNHAMLQTLMRLKRPNVAPAPCCVPTRLNPLSMMYYEF--NELVVRHHEDMIATECGCR
>BranchiostomaNODAL
---------------------MPAPRPAMGTFLTASVAFFAVQLLQCEC--
LSNEHTLAASGLLRASGGEGLTL----GRYEHGDGVVNVTMRGMAVAGRMT---------------PE-FMLDFYQSLSSGTD-------------------
LNVTREENQALPLPSDTVRSFAL-------KDAKQKGSKKFSYVFDTSN-ISPDT-DIRLAELRMR--------GPHFSPPRRLSVRVFSH---
EEKKCKKKKATCTVKHRIASFKHIQEDLTADG-
WRVVDLTKKLSRWVRQNVSADTIEIQITSKPERRRPRDPVREERDTSFDESHSEDEESDFYGTDEPDAESRQKRRVEIKLSEDV
SLVVFSQDQKADLLRDSQKAKRAQHPVRHDHQAETRVAR-RRRQKKSKKG-----------------------
KTDSSKKEDTPCKKVEFWVDFD-HIGWGTWIIYPKRFNAFRCEGVCPTPVDQL-
YHPTNHAVMTSILNLHKPGKAPMPCCIPTKLKALSMLYLEH--GEVVLRHHEDMIVDECGCQ
>mouse_Nodal_P43021
-------------------------------MSAHSLRILLLQACWA---
--------------LLHPRAPTAAALPLWTRGQPSSPS--------------PLAYMLSLYR---DPLPRADIIRSLQA-------
QDVDVT-GQNWTFTFDFSF-LSQEE-DLVWAELRLQLPG-----PMDIPTEGPLTIDIFHQ---AKGDPERDPLERIWMETFTVIPSQ--
VTFASG-STVLEVTKPLSKWLKDP---RALEKQVSSRAEKCWHQP------------------------------------
YTPPVPVASTNVLMLYSNRPQEQRQLGGATLLWEAESSWRAQEGQLSVERGWGRRQRRHHL----------------------------
PDRSQLCRRVKFQVDFN-LIGWGSWIIYPKQYNAYRCEGECPNPVGEE-
FHPTNHAYIQSLLKRYQPHRVPSTCCAPVKTKPLSMLYVDN--GRVLLEHHKDMIVEECGCL
>NP_571041.1 nodal-related 1 precursor [Danio rerio]
-------------------------MFSCGLLSVVLALAVGLVSCTG--
-------------------------NLAGFQETLRNKMRAAGRNGAGHGRHLT----RYPL-YMMHLYRTLLTGDE-----------------------
KHFSHENPTLYESDSVLSLVA--------KSCHQVGDKFAVTFDMSS-ISASD-DVQRAELRIRLPH----------LRSELEVDIYHA---
STPECERSP-CEEVRVHLGTLNANINSTFRSS-WRIFNITALLKYWLHQS---ERVPFEEPTQMPPMAEGH--------------------------------
---KSVHHPTANRVMMVVYSKQNRAKTSTLIRTAEHSKYVALDRAGGGSEPV--PRRHRRNHRT-DDRVRDAAAGMIPG-------
VSHEGGEKKPLCKKVDMWVDFD-QIGWSDWIVYPKRYNAYRCEGSCPTPVDET-
FTPTNHAYMQSLLKLHHPDRVPCLSCVPTRLAPLSMLYYEN--GKMVMRHHEGMVVAECGCH
>NP_624359.1 nodal-related 2 precursor [Danio rerio]
----------------------------------MHALGVARLACYC---
----------------------QLLLLGVFGKHTRYRYNSLRNMSHRM-HLPT-YMMHLYRHYKMNQT---------------------------
RIPAESLEHEHADTIRSIMS--------KNVLNNDNHYVAIFDLSPVLSERQ--IQAAELRIR--------
VPRDLHPDGLTLELQHQQGAPCPRHQPCP----KSQSLGLLPEELLSVTQH--WRVYNVTNLLLHWPRPR---
TSPRSRVKTKRPAAAPGGPAVAPGAQELQSGPQRPAAAPGGQKIQNRPRRPSAAPKGQEVQSRPMLLLFSEQQDGASLLHTA
GASKFLFSRNKKEVKRGRALR-SRRGRRGPPVRSP-------------------LQRTPLHKSTTCRRVDMHVDFN-
QIGWGSWIVFPKKYNAYRCEGACPNPLGEE-LRPTNHAYMQSLLKYHHPSRVPASCCAPTRTSALSMLYYEN--
GEMILRHHEDMQVEECGCL
>human_BMP3_P12645
------------------------MAGASRLLFLWLGCFCVSL---
----------AQGE----RPKPPFPELRKAVPGDRTAGGGPDSELQPQD-----KVSE-HMLRLYDRYSTVQA-------------
ARTPGSLEGGSQPWRPRLLREGNTVRSFRA--------------ERKGLYIFNLTS-LTKSE-
NILSATLYFCIGEPVSGGCSHHAQRKHIQIDLSAW------------TLKFSRNQSQLLGHLSVDMAKSS-
WLSKDITQLLRKAKENEEFLIGFNITSKGRQLPKRRLPSHIRAALSIERRKKRSTGVLLPLQNNELPGAEYQYKKDEVWEERKPYK
TLQAQAPEK------------------------------NKKKQRKGPH--RKSQTLQFDEQTL-------KKARRKQWPRNCARRYLKVDFA-

DIGWSEWIISPKSFDAYYCSGACQFPMPKS-LKPSNHATIQSIVRAVGVPGIPEPCCVPEKMSSLSILFFDE-
NKNVVLKVYPNMTVESCACR
>Crassostrea_gigas_mGDF2_CAD67714
--------------------MKLYYECTFPTVLITVVQLTIVSFA---
KPASVTTFAGTDHQER----TNSHRIEVFKQKILDGLQYKNVPRVTSFNE------TIEKRKLIQMYRNYMRKRD---------------------------
RNYPQESEPITGTARMHRYNLRPGNEKASNEKRLKLFVVPE-LKHTRERVSSARLKLFKHT-----SLATTRTSEVEIKLFNN-----------
-----NQVIDTLVESRTIDLSRDG-WEIFDITQDVQDWIEDPELNNGIEIFVDGLDAGQLVFP----------------------
SLNITERMSSKSSTNTTIPNVILPILEMKTHERSI-----------------------------LKRVKRQNDI-ER-------------------
RDCVKGDGESRCCRFTTTIAFS-DLGWNDWILAPPDYEAHYCDGSCPDRF----
KMANTFAGIQARLHALYPNKFPKPCCVPSKLSPLTILHKDS-SGKYQLTDYPDMIVEDCKCA
>BiomphalariaTGFb

MSNLAPHAFISLLALLLMSGTNVHSLHTKSLSNSSNNKKSNQHQNGFTEDSSLSNKDYTWDSNNDINNISDTKTSKFIANELELE
QNNHKIFNSSLVFKLSDNENDQLSSN-
TNLSHFNVSFENYADIKAKTSHCLSCQIRNDSRDFRIQSIKHQILKHLNFKSLPNATRNAIP-----KMPA--LYHIYNANPELLN----------
------DDPHSHGHESDTDEFVVKTERVFTAAK------EASSPNFNNISDSVFFSQPAQLATAR--VRSAKLWIYIRK-------
AELQRPYLLLTVRRIFLEQGT-----------NVILKFVYSSINVSKAFG-WKRIELRDIVVDWMKHPLFDIGLQIRAENDKGQNLVVL--------
--------------------------PPTDDIDKGYEPWLDIKIQEVRS-----------------------------NSRHKRSDS-----------------------
LVCSGNTTENRCCRYPLYVSFI-DFGW-EWIIAPTHVKADYCSGECRMTM----QDNTPYSWVNQQLPG-----
SAGSCCSPIKMSALPLLYFDE-NHNILYQILQNMKVEKCGCT
>Mouse_Myostatin_AAC53167
-------------------------MMQKLQMYVYIYLFMLIAAGP--
VDLNEGSEREENVEKEGLCNACAWRQN----TRYSRIEAIKIQILSKLRLETAPNISKDAIRQ----LLPLRELIDQYDVQRDDSS---------
-------------DGSLEDDDYHATTETIITMPT-----ESDFLMQADGKPKCCFFKFSSKIQYNK--VVKAQLWIYLRP-------VKTPTT-
VFVQILRL---IKPMKD-------GTRYTGIRSLKLDMSPGTGIWQSIDVKTVLQNWLKQPESNLGIEIKALDENGHDLAVT-------------------
----------------FPGPGEDGLNPFLEVKVTDTPK-------------------------------RSRRDFG-----------------------
LDCDEHSTESRCCRYPLTVDFE-AFGW-DWIIAPKRYKANYCSGECEFVF----LQKYPHTH---
LVHQANPRGSAGPCCTPTKMSPINMLYFNG-KEQIIYGKIPAMVVDRCGCS
>XP_001641598_2Nematostella 4907 bp
---------------------------MFLTPTLFIAFLALCECSR--
-CPLCADPMEN----LKQDRLQAIQQQILDKLGLPFAPNLTDPKIP-----NIPP--LLRLLETSRNAEL--------------------
AASRVKHEDNYHAKTKTIIMFPE------KAPTIIREHSAKCCFFQFREKASRLR--ISKATLDVFVKR----NPNASSTNRDPQIKLYKR---
VFPVTEG-------IPEKELVTTKPIRPGQSG-WYHFKVKKLLREWRREPHKNLGVEFEIEGNDGSLSLVT------------------------------------
--EGNEAKKPFLEVFTHDTGN-----------------------------KRRAKRRAG-----------------------LDCDPYSHERRCCRYELTVDFE-KFSW-
NWIIAPKRFRAYYCTGECQEIM----LPMYPHAHMVK------KSTGKRPCCSPTKMSSISMIYFDF-NQQIMFEEVPAMVAETCGCT
>Drosophila Myoglianin_AAD24472
RFNLDSKKINIGRMNILGHKRGNFRVTRYVTVTVLLILSTAVNAYAQPENKSFSNSSNNDSPEMVMLSNETNNSAKVLSEKNGSS
SISADDKINENMGIFQMKVQSKPGKKSTPLAKVSEHGDLSRVQSVSLYRN----------TLI--NIESMLQRQLREK----
AKVDSIESIKMHILMRLNLKKLPNITKPI-------SVPQ-
NIIDNFYRDYNASSTVNEFLIDLNKNQAKKSDIPINTNDEEYESILSHISSIYIFPE-----EIQPHVRHNRKVDVFRFQIDS---SYS-
DLSYATLHLYLRGPGLLEEIKKQPRKDIVVTIHRAIRVANTTSF--------NPKVKMFEFRHSIPSGLGQWVAVDLKSLLGNL----
GSNMTQEILIKGAETWMKSLV-------------------------------------VTTDNTSKNPLTVHIEIGSQ-----------------------------KHRRKRSVY----
-------------------MDCTENDHDMRCCRYPLKVNFT-SFGW-HFVVAPTSFDAYFCSGDCKVGY----LEQYPHTHLAAL------
TTSATPCCSPTKMSSLSLLYFDD-NHNLVLSVIPNMSVEGCSCS
>Drosophila_Activin_2_AAN10416

MAKYFIALVLLVCLALENRNVYNRLHARSHPSSRGDILRPHPKHQPHPHVQHTSQQLQQQHHIRQQRSRQLKHLEPDPTSEED
DVPITKREYYARLRRHVIHKRQHLLQQITISVKSSAGGCPKCESNRQVEHITEEQ----LTHLRIEFVKQQILEKLRLKESPKVSAVEL-
-------PK-PIFDGMTLSHPDDS---------------------TKNKELDDYYARTSKKFILLNR-EEVECNRARDGKSNPSMCFTFKIDD-
ADAEGFDVSTAVLWLFKNKNRTDTASVNSTSAQQTIVVSEVEVDQQKDSKY-------LSAAKTIAIQSVNVQDE--
WMKIDIEWPIKHWISGHELSHLIQITCGGCDVSDMEEI-------------------------------------ISVDKDYRPFIVIDMQNRRR--------------------
---------KSRQKRS-------------------------INCSSG--MTECCREHLYISFR-DIGWSNWILKPEGYNAYFCRGSCSSVASVT-
QAASHHSSIMKILSTANKSLELVPCCTAKQYSSLQLVVMDS-SNTATVKTLPNMVVESCGCR
>HyS0035.111
-----------------------------MSYHIIFTLAVAVACTS---
GNPIQTDNSKNSPVEIGLGESGVDNETM----NYVERVEKIKRDILLKLGLTKPPSERFSRA------NIPS-AILSNFMLDEDIRR-----------
-------------RKNEENDGYTRSRVNIIGK-------IERYRLCGSKRCLQVRFKN---KPFNLVDSARLWIHVAS-------RQNITR-STIKLYKV---
-------------AGNRKKLLQISRSRSLRPG-WYAMEITKHIIRITSTGESYMDFILEIESENRLFELSK---
KADVPFLELTTKTKIG------------------------------KRKRRSAE-----------------------ENCTS---KSACCRAEFVINFK-
DIGWDDWVVSPDKYNAYYCNGHCKNKY------NYRHAGILQAATSQLKRKDVKVCCTPYKYSSLKLLYWNE--
GNMYEKVIDHMIVDECRCP
>HyS0118.21
-------------------------MSLKSRALLFLIFQKVCMRYF--
GESLPTTCDGKCGQKFINDTL----IHQQRIEMIKQEILDKLGLQAPPEKRGSLN------DIPK-ALLDGFRRSVEKSR-----------------------
-RLKIMKERENVKKMIILAE---------NVSSGEKRQVIKLKYAS---QSHFSVENMRLWLYIKP--------INTSRISVISVVQS---SKEK---------
-EQIQRIMLRKKLNYFKTG-WFEIKIND-IKTWTRKTYSGVDLKLRIHCKNCKLEM--
SGENQPFLELTEKSRVL------------------------------RRNRRN-------------------------IMCRKD--SKVCCLQRFYVSFK-
DIGWDDWIISPPGYNAQQCRGSCDSLG----VGSTDHASIIKAVADKR-KIDAKLCCTAQQYDSLSILYKDS-
EGNLVKQGIKDMIATHCGCG
>XP_032230527.1 inhibin beta B chain [Nematostella vectensis]
----------MLLSLRRLPLAMMPAMELSMCCYVLPIILSAVLASQVSATARRNVTADNALLNATRYKHVQYLGNAT--------------------------
---------------------------------SVRLQFPTGDKTALTGCKNCGVKQPPKPGSLEYKIRIDMIKTQILQKLHMDSEPKIKVPKL------SIPT-
PLMDSYFLETTLNR---------------------DRGKDVSESEEGKVSQVIVIGRKVASPPRLPRQRQSRKAHLFEYRFSKKIHTKH--
ISSAVLWFYKNS---------THQMEESTLVFQDSATRKHTRGPLP-----TSKHHTVASKQLHPSDHG-
WISVNVLSMVRQWIDNAEAKTNLGVSCPNCRSLLADLI--------------------------------------ASNDQYRPFITLGLGETRK------------------

------------QRKKRA-------------------------IHCSPG--MRECCRQEFYVSFE-EMGWDNWILVPRGFNANYCTGSCYGHI----
LPVYHHTEVIQKVALLRKQRELSPCCAPTKMFDLSLLYYDK-DENLFQENVSNMVVEECGCS
>human_activin_bB_P09529
--------------------MDGLPGRALGAACLLLLAAGWLGPEA--
PPPPGSPGGSQDTCTSCGGFRRPEELGR----VDGDFLEAVKRHILSRLQMRGRPNITH---------AVPKAAMVTALRKLHAGKV-----
-----REDGRVEIPHLDGHASPGADGQERVSEIISFAE---------TDGLASSRVRLYFFISN-EGNQNLFVVQASLWLYLKL--
LPYVLEKGSRRKVRVKVYFQ--------------EQGHGDRWNMVEKRVDLKRSG-
WHTFPLTEAIQALFERGERRLNLDVQCDSCQELAVVPV-----------------------------------FVDPGEESHRPFVVVQARLGDSR----------
------------------HRIRKRG-------------------------LECDGR--TNLCCRQQFFIDFR-
LIGWNDWIIAPTGYYGNYCEGSCPAYLAGVPSASSFHTAVVNQYRMLNPGTV-NSCCIPTKLSTMSMLYFDD-
EYNIVKRDVPNMIVEECGCA
>human_Inhibin_bA_P08476
------------------------MPLLWLRGFLLASCWIIVRSSP---
TPGSEGHSAAPDCPSCALAALPKDVPN----SQPEMVEAVKKHILNMLHLKKRPDVTQ---------PVPKAALLNAIRKLHVGKV----------
GENGYVEIEDDIGRRAEMNELMEQTSEIITFAE------------SGTARKTLHFEISK-EGSDLSVVERAEVWLFLKV----PKANRTRTK-
VTIRLFQQ---QKHPQGSLDVGLKGERSELLLSEKVVDARKST-WHVFPVSSSIQRLLDQGKSSLDVRIACEQCQESGASLV---------
-LLGKKKKKEEEGEGKKKGGGEGGAGADEEKEQSHRPFLMLQARQSEDHP---------------------------HRRRRRG-----------------------
--LECDGK--VNICCKKQFFVSFK-
DIGWNDWIIAPSGYHANYCEGECPSHIAGTSSSLSFHSTVINHYRMHSPFANLKSCCVPTKLRPMSMLYYDD-
GQNIIKKDIQNMIVEECGCS
>Drosophila_Activin_1_O61643
QPAAMRKKVADLEVLRVSRFVAVILVLARWVTAVATLLTSCILLDIFSVPGQSGVADRSQASSRTVHVSVPTTPNETPSSTSETKL
KLLYGYTSYDINNDQQVKSNNLCRVLCKSRNRKRQRRRRRRRNHRRRRH-
NPHTDYNIYNKYHSNNNFNKKTNQPHNNIAPSDEVRLESIKRQILTKLGLSHKPNVSRNRSLERIDKSMPI-
ELKSHHNSSPKELKQPAIHNIEYENQKGHHESFADDHENIDHEDFFGNTQEIITFAE---------
EGTQYRQYRILEFSAQNRRVPSQKLIRSAQIHIRIDKTKRKWGANKPHHR-IKIWVFQL------------
ITEKGIDKAIIFRASQVDPKNLG-WQKFDLTDTIREWYGHTHEKLRLLIDCTGCGGRYSLHL-------------------------
FQTSKLRGNSSDYLSTNPNRPFLVLHTESSRT-----------------------------RRVRRRA-------------------------VDCGGAL-
NGQCCKESFYVSFK-
ALGWDDWIIAPRGYFANYCRGDCTGSFRTPDTFQTFHAHFIEEYRKMGLMNGMRPCCAPIKFSSMSLIYYGD--
DGIIKRDLPKMVVDECGCP
>BranchiostomaTGFb
-----------------------------MRPLVGYMFSGLVLMNA--
VVSSSANCRLDIGE----YKKRRIHAVKGQILSKLGMSDSPTDPGPA-------QTPE-DVMAVYNQTRDLLA-----------EQSLQREALC-
TDWVKDEYYGRTVTRFPAVPNGDKDHMYENLRQEKRFSLFFKFDVQS-LYQNVSDVTEAELRIYQVP-----NPMADLPE-
QRIELHQL---LPPINGNG------SPRQRYLGSDVVKTRDKG-
WLSFVVTNTVREWLINPASNLGLELTVHCAGQTFDANLELTSDTPEVLETLIAGTPEAEAIDDG-RGDTAQVDS----
PQKKEPHLLVFTKRPPPET---------------------------HSRRKRSLDS----------------------SYCFKKPKEPNCCLRELYIDFQRDLGW-
KWIHAPKGYNANFCAGSCPYLW----STDSQHSTIIGLYSTLNPHASASPCCVPSELEPLTILYYQG--RKPRIEQLSSMVVTSCKCS
>danioTGFb2
-------------------------------MNLYVLSLLLLLDLA--
AVAVSLSTCSTVDMDQ----FKKKRIEAIRGQILSKLKLSSPPEIYPEPE------EVSR-GIIAIYNSTRDLLQ-----------
EKANERAATCERQRSEEEYYAKEVHKIDMQPF-YPSENVILSQHYYPYFRRLMFDVSS-MEKNASNLVKAQLRIFRLQ-----
NPKARVFE-QRIELYQV---LGHKDLT-------
SPTQRYINSKVVRTRTEGEWLSFDVTEAVSEWLLHRDRNKGFKISLHCPCCTFVPSNYIIPNKSEELETRFAGID-DSFVN---
GGDLKMFK--KRRHSGQSPHLLLMLLPSYRLESQHK-----------------------SHRQKRALDT----------------------AFCSRNV-
QDNRCLRSLYIDFKKDLGW-RWIHEPKGYNANFCAGACPYLW----SADTQHSNILGLYNTINPEASASPCCVSQDLEPLTILYYIG--
KTPKIEQLSNMIVKSCKCS
>human_TGFb2_P08111
-------------------------------MHYCVLSAFLILHLV--
TVALSLSTCSTLDMDQ----FMRKRIEAIRGQILSKLKLTSPPEDYPEPE------EVPP-EVISIYNSTRDLLQ-----------
EKASRRAAACERERSDEEYYAKEVYKIDMPPF-FPSENAIPPTFYRPYFRIVRFDVSA-MEKNASNLVKAEFRVFRLQ-----
NPKARVPE-QRIELYQI---LKSKDLT-------
SPTQRYIDSKVVKTRAEGEWLSFDVTDAVHEWLHHKDRNLGFKISLHCPCCTFVPSNYIIPNKSEELEARFAGIDGTSTYT---
SGDQKTIKSTRKKNSGKTPHLLLMLLPSYRLESQQT-----------------------NRRKKRALDA----------------------AYCFRNV-
QDNCCLRPLYIDFKRDLGW-KWIHEPKGYNANFCAGACPYLW----SSDTQHSRVLSLYNTINPEASASPCCVSQDLEPLTILYYIG-
-KTPKIEQLSNMIVKSCKCS
>danioTGFb3
------------------------------MHLGKGLLFVLLLNSV--
TMSLSLSTCTTVDIDN----IKKKRVEAIRGQILSKLRLTSPPQTLGPS-------QVPY-QVLALYNSTRDLLD-----------
GFGKDRHQSCGQDNTETEYYAKEIHKFNMIQG-SPENNDLPYCPKGITSKVFRFDVSI-MEKNASNLFRAEFRALRMP-----
NLSTSRTE-QRIELYQI---LRPDEH--------
IGKQRYIGRKNVMIGGTDEWVSFDVTETVREWLTNRATNLGLEISVHCPCHTFRPNGEIIENVNEALEVKFRGMDVEDDGPI--
RSDMGRLK---KPKEQNLPHLILMMLPPHRLDVLPT-----------------------SRRRKRALDT----------------------KYCFSNY-
EENCCVRKLYIDFRQDLGW-RWIHEPKGYHANFCSGPCPYLR----
SADTTHSSLLSLYNTLNPEASASPCCVPQDLEPLTILYYVG--RTPKVEQLSNMIVKSCKCS
>Human_TGFb3_P10600
----------------------------MKMHLQRALVVLALLNFA--
TVSLSLSTCTTLDFGH----IKKKRVEAIRGQILSKLRLTSPPEPTVMT-------HVPY-QVLALYNSTRELLE-----------
EMHGEREEGCTQENTESEYYAKEIHKFDMIQG-LAEHNELAVCPKGITSKVFRFNVSS-VEKNRTNLFRAEFRVLRVP-----
NPSSKRNE-QRIELFQI---LRPDEH--------
IAKQRYIGGKNLPTRGTAEWLSFDVTDTVREWLLRRESNLGLEISIHCPCHTFQPNGDILENIHEVMEIKFKGVDNEDDHG---
RGDLGRLK---KQKDHHNPHLILMMIPPHRLDNPGQG----------------------GQRKKRALDT----------------------NYCFRNL-
EENCCVRPLYIDFRQDLGW-KWVHEPKGYYANFCSGPCPYLR----
SADTTHSTVLGLYNTLNPEASASPCCVPQDLEPLTILYYVG--RTPKVEQLSNMVVKSCKCS

```
>human_TGFb1_P01137
----------------------MPPSGLRLLPLLLPLLWLLVLTPG----------------------------------------------------------------------------------------------------------------
-RPAAGLSTCKTIDMEL----VKRKRIEAIRGQILSKLRLASPPSQGEVPPG-----PLPE-AVLALYNSTRDRVA-----------GESAEP-----
EPEPEADYYAKEVTRVLMVET---HNEIYDKFKQSTHSIYMFFNTSE-LREPV-LLSRAELRLLRLK---------LKVE-QHVELYQKYS----
-----------NNSWRYLSNRLLAPSDSPEWLSFDVTGVVRQWLSRGGEIEGFRLSAHCSCDSRDNTL----------QVDINGFTTGR------
RGDLATI------HGMNRPFLLLMATPLERAQHLQ------------------------SSRHRRALDT----------------------NYCFSST-
EKNCCVRQLYIDFRKDLGW-KWIHEPKGYHANFCLGPCPYIW----
SLDTQYSKVLALYNQHNPGASAAPCCVPQALEPLPIVYYVG--RKPKVEQLSNMIVRSCKCS
>danioTGFb1
-----------------------------MKAESLLLALQCLLGFV------------------------------------------------------------------------------------------------------------
QYSRALSTCNPLDLEL----IKRKRIEAIRGQILSKLRLPKEPEVEEKELIE----NIPA-ELISVYNSTMELNE-----------
EQAANPVQHTIEDPTEEEYYAKEIHKFTMMEE--------------KPEKYLVFNITD-IKANH-VLYQAEFRL-RIK-----EPKMGDSE-
QRLELYQV----------------TGNKSRYLNSRFISLQTAGKWVSFDVTSTLKDWLQMPEEKQEFQLQLACSCKPESQNT--------
EFLFKIAGLSRN-------RGDTGLLA-----DQVAKPYILVMSHPADGHSPA-------------------------KSRRKRETD----------------------
AVCTEK--SEGCCVRSLYIDFRKDLGW-KWIHEPSGYYANYCTGSCSYVW----
TSENKYSQVLALYRHHNPGASAQPCCVPQVLDPLPIIYYVG--RQHKVEQLSNMIVKTCKCC
>GLS-HyS0012.146
------------------------------MSSLLIFLMLLSVIDP-------------------------------------------------------------------------------------------------------------
-------------------------VVLVINNDPSKGNTRNP-----QVPQ-FMMDLYNVVADENG-------------------------YARKGAPNLGKTINCVSA-----
-HDSRHNSTSGRFRYQFNLSS-IEADL-IIRRVELRVFKTK------STRRENVFYRTDIVLD-----------------GPSYRDIRSVMVSSTGYG-
WLSFEITDVASRLIQYGDKIVDIDITLQPIRNKLPTFK-----------------------------------
LLTSTNTSTRALFMVYSDTNVKSSKKASRLYGTGKYAGVQE----------KNRHRRDAKNS-----------------------------
RQACRLANLTITFS-
EHTAFGDVMLPRKVNIRRCEGSCRYLVGAGTVEETYHGVMQNMIKRPSTKRIRYACCAPRYLQSLLLLRQSSITGDIVMVTLDNA
IVETCWCV
>Podocoryna TRINITY_DN90629_c0_g1_i1 len=1471
-------------------------MQLKFSSTHSVLFYVAMVAEL--------------------------------------------------------------------------------------------------------
---------------------------------VALSNASSKTKSNTLKL-----QVPQ-FMMDLYNVVADKNG-------------------------
YSRNGAPDLGKTITCISA------HDSRYLNKSHAFHYRFNLSS-IEKNL-NIKRVELRVFKTK------SKRRENIFYRTDIMLD----------------
-GDSYRDTRSIMVSSTGYG-WLSFDVTNIARQLIKYDDKAVNIDITLRPIRNQLPAFR-----------------------------------
LLTSMNTSTRALFIVYSDGPGSLSQHTSDKGTLLPYDTSKN----------HNRQRRNARKT-----------------------------
RHACRLLNLTINFD-
KHDEFKNVIIPSVAHIQRCAGSCRLPSGFGSAEETYHAIMQNMIKRVEKKRMKYACCAPRELHSLLMLTKCSSSDDIKMRTLEDA
VVASCWCV
>HyS0079.35
---------------------------MFKMGPTLFIFYALFVCNI-------------------------------------------------------------------------------------------
QNIPATPVNIIGIKK-------------VQETVKNYIKEKKYEENKEDEEEEERLRKA------HIPE-YMRNLYKILNKQAD----------------------------
AGLLHDANIVRSYFD-------QGTTTTEMEQQSYQFDIVD-INRKREKLIKAELRIFKYP----NRKRTPLASFVQIDVKDK------------------
NTNKIISSRILTSSGSG-WQVFPVTNIVSAWISKEHLNKGVQLSVRSLYGANEEIK-----------------------------------
FATKKTVRREPILVVYTKDSYDLVLATLSNLKQNAAAFFATKTNAEPSAEEPRRVRRRAR-------------------------------
FDPCRVEELSVPLV-
QIGWHKSFAAPRKFRINQCKGTCGYDASKAYHKQTNHAIVQALYAAITGRKTSYPCCAPSKFAPGNALIVDRSGEVVKVVSLEKL
TVVQCQCL
>Podocoryna_TRINITY_DN13881_c0_g2_i3 len=2420 path=[0:0-422 2:423-457 3:458-950 4:951-999 6:1000-1033 7:1034-1096 9:1097-1120 10:1121-1159 12:1160-2419]: 2311 to 1157: Frame -2 385 aa
------------------------------MEYMVFVFYVLFVCNI------------------------------------------------------------------------------------------
EYTTATPVSYIETNKVVNTDTEKFVNHFNEQHRIDRENDVTVEEEEKEAIEEEKLRKA------HVPK-YMRDLYKVLNKYAD------------
----------------EGLLHHGNIVRSFSD-------QGNAKSERNLKKYQFDISK-ASKDTETLTKAELRLFKLP----NRKKGSLSSYIQIDVEDV--
----------------YSGKIVSSKVSSIHSNG-WQVFPLTGIVKIWLADQSKNKGVRVTARSLYGDNNGIT-----------------------------------
FATKDVISKEPILVVYTKDHNELTLASLLKFKVNTTALFTKET-----SDYHKRERRDSG-------------------------------
YDLCHLEKLSVPLD-EIGWDKNFASPTKFKINQCKGVCRHDSTDS-
TKQTNHALIQALYAAVTGRKVSYPCCAPAKYASGTALVVDRTGGTVELVYLQKMTVTRCECL
>human_GDF9_O60383
-------------------MARPNKFLLWFCCFAWLCFPISLGSQA----------------------------------------------------------------------------------------------------
SGGEAQIAASAELESGAMPWSLLQHIDERDRAGLLALFKVLSVGRGGSPRLQPDSR--------ALHYMKKLYKTYATKEG--------------
-----------IPKSNRSHLYNTVRLFTPTRHKQAPGDQVTGILPSVELLFNLDR-ITTVE-HLLKSVLLYNINN-----
SVSFSSAVKCVCNLMIKEPKSSSRTLGR------APYSFTFNSQE-FGKKHK-
WIQIDVTSLLQPLVASNKRSIHMSINFTCMKDQLEHPS-----------------------------------
AQNGLMTLVSPSLILYLNDTSAQAYHSWYSLHYKRRPSQQERSLSAYPVGEHHRHRRGQET------
VSSELKKPLGPASFNLSEYFRQFLQNECELHDFRLSFS-QLKWDNWIVAPHRYNPRYCKGDCPRAVGHR-
YGSPVHTMVQNIIYEKLDSSVPRPSCVPAKYSPLSVLTIEP-DGSIAYKEYEDMIATKCTCR
>Drosophila_Maverick_AAF99658
IRNSKAREPPCKHPPITDNAEAKLTLVCSRSLKLYKIWFILILLMSTQHQVSGHGIFRQTSSSKAVFSYYQPKENITFTNLQLQNLE
NEAKRLETNQHPIIRAKSTPKMGLKNVFESFSKQSRDSIYNASSNKYS----------RQTRETRDIGLETKRHSK----
PSKRVDETRKHLVLKGLGIKKLPDMRKVNI------SQSK-
YIEYLSRLRSNQEKGNSYFNNFMGASFTRDLHFLSITTNGFNDISNKRLRHRRSLKKKHQNYGDLLRGEQDTMNILLHFPLTNAQ
DHDK--IDEANVRLMRGPDVNLNQSNKNSSQQLTLKVYQL---------------LSANRRRKITSRKIQETRTQ-
WIEFDVTKAVRSWLNKSHENLGIEIQCDKCKSIGARIL----------------------
SDFSPSTPPRSTASSDEHLNLMPVLNIIGHGTLNSQQHGDADIHQIMLTNNRSD---------QYVHHRSNHD-STWRKDKWT-----------
-NNCYKL--HQRCCRNQLDVAFK-SIKGFEFILQPKVFDAGYCHGRCPPRH----
NPAHHHALLQSLIWQEDHKRAPRPCCTPSKLEMLEILHVDEHSDKLKISTWSDMQVVECACS
>human_Lefty_O00292
------------------------MWPLWLCWALWVLPLAGPGAAL-------------------------------------------------------------------------------------------------------
---------------------------TEEQLLGSLLRQLQLSEVPVLDRADMEKL---VIPA-HVRAQYVVLLRRSH------------------------
GDRSRGKRFSQSFREVAG--------RFLASEASTHLLVFGMEQRLPPNS-ELVQAVLRLFQEP--RHGRLSPRSAQ-ARVTVEWL------
```

------VRDDGSNRTSLIDSRLVSVHESG-WKAFDVTEAVNFWQQLSRPRQPLLLQVSVQREHLGPLA------------------------
SGAHKLVRFASQGAPAGLGEPQLELHTLDLRD----------------------------YGAQGDC------------------------
DPEAPMTEGTRCCRQEMYIDLQ-GMKWKNWVLEPPGFLAYECVGTCQQPPEALA--------------FNPFLGPRQ-
CIASETASLPMIVSIKGRTRPQVVSLPNMRVQKCSCA
>Human_GDNF_P39905
-----------------------MKLWDVVAVCLVLLHTASAFP--
---------------------------------LPAGKRPP-----------EAPA--
EDRSLGRRRAPFALSS---DSN--MPEDY--
PDQFDVMDFIQATIKRLKRSPDKQMAVLPRRERNRQAA--AANP------ENSRGK----------------------
-----GRRGQRGK------------------------------NRGCVLTAIHLNVT-DLGL--GYETKEELIFRYCSGSCDA------
AETTYDKILKNLSRNLVSDKVGQACCRPIAFDD-DLSFLDD---NLVYHILRKHSAKRCGCI

- **TGFβ receptors alignment**

>XP_057305023_1_activin_receptor_type_1C_like_Hydractinia_symbiolongicarpus
KRCMIHCLLLLYLTFKQFNGGVGNGQCYCNGVDGGTNNVCLH--ECYTVLHKNGH---ME
KGCI---------LTHCFEIKNAHKHERLINCC-KGDMCNKNVGEFIEPPTKSKEIISIP
---------TAAAIAVPASLIFLLATL-FVLYKDRRKKRLMRRIAEENHLLTQRLDEING
INLVTTSGSGAGLTLLVQRTIARQ-VLLHERIGAGRFG-SVHRGTWHGQEVAVKIFSSNE
EASWFREAQIYQTTMLRHENVLGFIAADN-K-DT-GSCTQLWLVTEYYPFGSLYDFL-QE
QYLTPRETLKMVYSMINGLCHLHMEVIGTE---G-KPAMAHRDMKTKNILVKNNKTCAIA
DLGLTVLHTSINDKVDMPTG-TRTGTTRYQAPELLSEIVNFS-YFDSYRRADVYAFGLCA
WEVCRRTE--ID-GHVDEYQLPYYDCV-QPDPSHEEMKKVVCDENRRPAF-AEQ-WHTEE
PMKILVKIMKECWYPKGAARLTSLRIKKNIVAICRELDIKVTY----------------

>XP_057304402_1_activin_receptor_type_1_like_isoform_X2_Hydractinia_symbiolongicarpus
KLIGEAGNVLNYTKNVRHNSGKRV-RCHCD--GCA-NHVCESYAGCFSSVTHKSGALTKR
YGCLDIEQMHLRLRMYCANINGTVTNGITMRCC-ETDDCNAVFPPARHCMSNLSSATPTN
TSHSFSKN-MAINIGTPVAALIFLLAVIIGAFKLWQRKSLKFIKKKENQMQPGTALTPLL
PGDDCSSGSGSGSGLPFLVQRTVARQ-IVLCDLIGRGRYG-EVWRGSWQGESVAVKIFNSRD
EESWKRETEIYNTVLLRHDNVLGYIAADI---ATRHGVTCMWLVTHYHMHGSLYDYL-NL
HTLDPHQMCLIAYSAVSGLAHLHTEIFGMQ---G-KPAIAHRDMKTKNILVKKNGQCVIS
DLGLSVLHSSDSNKLDLAIN-RRVGTKRYMAPELLNETMCLK-SFDAFKQADMYAFGLVL
WEVARRCD-----GVSEDYQPPYYDKL-CQDPPFEDVKRVICDESYRPRI-PKK-WYGEE
HLMTLAKLMSECWYETPVARLTALRVKKTLGRLMERLDNEKKLFCKSIQFSMLTSSDKVD
SLSE
>XP_057303824_1_TGF_beta_receptor_type_1_like_Hydractinia_symbiolongicarpus
MLFLTLQCFYVLWNLVAG---ASNLTCYCNSHRRCTNNKCFLDGFCYIMRKNGKSTTFTE
KGCL-------------FVDNGLNKNKDNYMSCDNEMMCNMNLVPSSRNVTETNLPTTLK
STKHFTTFHITLLVVLPFMIGVSMMI-AWLVCKYKKGRNAGVDRSLLLHTVPASEERFRK
NEIDSSTGSGMGLPVLVQKTIAGE-INLTRRIGQGAFG-FVYHGLWRGQDVAVKVFSSTE
ESSWSREVKIYHTPMLRHENVLGFIAADS-K-DT-GTVTQQWIITDYHSNGSLFDFL-QD
NTLDITMLHKMLLSIVNGVLHLHTEIIGTF---G-KPAMAHRDIKTKNILVKQNFTCCIA
DLGLTVMYSSDKKHTDKPEG-TKTGTRRYQAPELLNNTLDQL-GFQVCRRADIYALGLCL
WEICRRTK--ID-GKVEEYQLPYFDAV-QPDPSSEEMIQVVCVEKRRPHF-SEH-WKKHE
SMRILMKTMTECWYHEGSARLTALRIMKDLSSHLVVEKS---------------------

>XP_057303312_1_TGF_beta_receptor_type_1_like_Hydractinia_symbiolongicarpus
MLFLTLQCFYVLWNLVAG---ASNLTCYCNSHRRCTNNKCFLDGFCYIMRKNGKSTTFTE
KGCL-------------FVDNGLNKNKDNYMSCDNEMMCNMNLVSSSPNVTETSFPTTLK
STKHFTTFHITLLVVLPFMIGVSMMI-AWLVCKYKKGRNAGVDRSLLLHTVPASEERFRK
NEIDSSTGSGMGLPVLVQKTIAGE-INLTRRIGQGAFG-FVYHGLWRGQDVAVKVFSSTE
ESSWSREVKIYHTPMLRHENVLGFIAADS-K-DT-GTVTQQWIITDYHSNGSLFDFL-QD
NTLDITMLHKMLLSIVNGVLHLHTEIIGTF---G-KPAMAHRDIKTKNILVKQNFTCCIA
DLGLTVMYSSDKKHTDKPEG-TKTGTRRYQAPELLNNTLDQL-GFQVCRRADIYALGLCL
WEICRRTK--ID-GKVEEYQLPYFDAV-QPDPSSEEMIQVVCVEKRRPHF-SEH-WKKHE
SMRILMKTMTECWYHEGSARLTALRIMKDLSSHLVVEKS--------------------

>XP_057297539_1_bone_morphogenetic_protein_receptor_type_1B_like_Hydractinia_symbiolongicarpus
SSFTLIILLILYEESVFS------IRCDC-HFHCPDNFTCLSDHKCFTIRKVIEGDTRLE
YGC-INKQGA---EFQC--ARSNSFVPNRIMCCDDYDLCNEDLIPVIEPEVINRYYENLN
ISKKEPVNNIALTISVTICVVFLVIALSYFYLR-YRQITSKRERDSERKLFITDGSTPVS
SYGEMSSDTGSGLPALTKRSIACE-ISFYEEIGRGQFS-VVYKGRWKQQYVAVKSICEED
QLSWNRELEIHKTAYLRHENILCYIGSDT-Q-EN-DTTQEVLIITAYYPYKSLYDFL-QT
HSFDYRILYRLAYSAVCGLHYIHRSISGVQ---G-KPAIAHRNVKSKNILVKENLTCCIG
DLGLAINIDPETREINFNSC-QKLPAIRYMAPEILEDTELLH-SFDTYQKSDIFSFGLVL
WELTRRYE--LA-GVPLDYQPPYFEYV-GTNPTLEDMRNIAVHKQIKPTL-PPR-FGDDF
HGKVMKRLIQECLHYSPSARLTAARVKKTLHKCVIDQNFEDSGGSNTCNTTSTGLSL---

>XP_057306640_1_activin_receptor_type_2A_like_Hydractinia_symbiolongicarpus
NQKWIIIFVTMALSFLDGLFQTLKSECRCDPTSDCTIEVC--KGSCYALAKNTTKVLKS-
SGCF------SCGVVGC---YEPQSAGIYLCCC-ENNLCNGDLNKIKKPTATTAVPVITG
PGNSKSHGATIIYIVTPIFGFVIVVAVLFKLWSKHKRMQARRAHLFSRSPP---------
----------STPPIQSKT-----IQMHDVVSRGQFG-YVWRACYEHKMVAVKVIPPHE
RMSWETERKMYEGYQLRHESILNFYSAEK-R-SE-DGMLQYWIITEYHEEGSLADYL-HK

TVVNFQGLLTLVLGMVSGLAYLHGEDTSSSVT---KPIIAHRDFKSRNLLVKSNRTCCIG
DFGSACSFGDSYEPEEAKAQ---VGTKRYMAPEVLEGAVAFQ--AEAFLCIDVYALALVM
WEVLSRCD--ASSAPVEEYRSPFEDEV-GLRPTMEDMRACVLDRKARPMI-KPE-WIQVE
KIAHLCDTIEDCWDDDADARLTAHCVLERITQLSRRPDWMEDEDYGVNTQLVDLSASEEK
IDFS
>XP_057293621_1_activin_receptor_type_2A_like_Hydractinia_symbiolongicarpus
ILFILVVGFSCTQFLFTNKDNVMREKCKHNDSFCI-NEECRNDIACMSYVIKSPSREYLQ
SGCISDNAHKKCVYHKCYQPAALTKKNEYYCCC-YGDLCNRNLTDDNWIKSKANSASTTS
VTPKENHSSMVVYIVLPIVLLGALFLVYLIKFH-KRTKLIKEPLLVELT-----------
-----------EVPLESETKA----VQVLEVIHEGRIS-CVYKASYQNKVVAVKMLHADN
KFMWDMERDMYEKHQLNHENILKFIGAEK-R-SL-SSMTQFWIITEYHNKGSLIDFL-QT
STVDVAQLFAMLLGVVTGLNYLHRGDLSTG-----RSIIAHRDLKTRNIMVKNDLTCCIG
DFGLAIPLSNTSPTDGATAQ---VGTIRYMAPEILQGAITFQ--RESYLAIDIYALALVM
WEVLSRCR--TA-DVTPEYEAPYVNEV-GYQPLLKDMCICVVSEKRRPVI-KEE-WRRDY
KMDDLCSTIEDCWDDDPDARLTVSCVKERLTRLSRDNEYPTYIGRKSSLVKEPDCFECTE
STGL
>XP_057316730_1_activin_receptor_type_2A_like_Hydractinia_symbiolongicarpus
----
MSCK-------------------------FLCL-KRSIRQPRVS-----DSDQPTLITTG
S---------------------------------RQKSCLEDGNTPKE-----------
------------LDSLSNFFAYET-VNFMELIYKGRSS-QVWKVSRGCHILTAKISTTKH
QV--QNEKRLFQDYQLKHENIIRFVHCLH-Q-AV-DNLKTDVLLFEYHPNGDLLNFL-KF
NIVPVSNALPLLKEIISGVAYLHSENMNTTPR---KYRIAHRDIKSSNILMKSDTRCCLA
DFECAYVFITNIKICEIIIAFLSVGTLRYLPPEVLAGTTDFD--EEAFFQADMYSTALIM
WELLSRCC--YSSDDVPSYMAPYENKV-GRQPNVNDLESCVLQQNYRPEFRPII-WK-DE
ELNFICKTIQDCWDEAPTARLTADCVYERLSSLM--------------------------

>XP_057294165_1_bone_morphogenetic_protein_receptor_type_2_like_Hydractinia_symbiolongicarpus
PKMFTLLLLAFLLDTDHYQLDRTKINCYVGSEKGSSYMKCEKNYSCFSHISFLVVITKKS
QGCWLRKKSGNCTSSACVERSGNNTIKSRFCCC-TGSLCNAKLQEKHDTTTLPTTYKVLV
RNDSKLIPTMVVSSITLVFIIVCLIVLYCWKQKQKRNTNARLSQAFYYGQYP--------
-----------ALEINIIASPTLDGILHGNLIHQGCHS-AVRLGTFNDKTVAVKVFPPRS
KLQWKNEKEVYGI--LGHPNIAEFITCDQIQ-GN-ESFEGV-IMMLYYKEGSLLNYL-RQ
RTLSWKQMLRMTYDVVNGLAYLHGESENTREGQL-RHGIAHRDLNSRNILVKSDGTCVLA
DFGFALQL--SGHPPEKYPL---VGSPRYMPPEVLDDSLVLTDWMSSMKQVDCYALGLVL
WELCRRCYDICQAADVPDCMLPYEEEL-GTGPSLRRVKYHVCRKRLRPKF-PVQ-WRKDD
SLSYFKLIMQECWDQDGDARLTSLCVLNRLKQLIDALSTEELERRETSLAPETCIYFDEG
RELN
>CAA80256_1_ALK_2_Homo_sapiens
DGVMILPVLIMIALPSPSPKVNPKLMCVCEGLSCGNEDHC-EGQQCFSSLSINDGFHVYQ
KGCFQVYEQG---KMTC---KTPPSPGQAVECC-QGDWCNRNITAQLP-TKGKSFPGTQN
FHLEVGL--IILSVVFAVCLLACLLGVALRKFK-RRNQERLNPRDVEYGTI----EGLIT
TNVGCTSGSGSGLPFLVQRTVARQ-ITLLECVGKGRYG-EVWRGSWQGENVAVKIFSSRD
EKSWFRETELYNTVMLRHENILGFIASDM-TSRH--SSTQLWLITHYHEMGSLYDYL-QL
TTLDTVSCLRIVLSIASGLAHLHIEIFGTQ---G-KPAIAHRDLKSKNILVKKNGQCCIA
DLGLAVMHSQSTNQLDVGNN-PRVGTKRYMAPEVLDETIQVD-CFDSYKRVDIWAFGLVL
WEVARRMV--SN-GIVEDYKPPFYDVV-PNDPSFEDMRKVVCVDQQRPNI-PNR-WFSDP
TLTSLAKLMKECWYQNPSARLTALRIKKTLTKIDNSLDKLKTDC----------------

>CAA80255_1_ALK_1_Homo_sapiens
LGSPRKGLLMLLMALVTQSRGPL-VTCTCESPHCK-GPTC-RGAWCTVVLVREEGHPQEH
RGC------GNLHRELC--RGRPTEFVNHYCC--DSHLCNHNVSATQPPSEQPGTDGQLA
---------LILGPVLALLALVALGVLGLWHVRRRQEKQRGLHSELGESSL----ILKAS
EQGDCTTGSGSGLPFLVQRTVARQ-VALVECVGKGRYG-EVWRGLWHGESVAVKIFSSRD
EQSWFRETEIYNTVLLRHDNILGFIASDM-T-SR-NSSTQLWLITHYHEHGSLYDFL-QR
QTLEPHLALRLAVSAACGLAHLHVEIFGTQ---G-KPAIAHRDFKSRNVLVKSNLQCCIA
DLGLAVMHSQGSDYLDIGNN-PRVGTKRYMAPEVLDEQIRTD-CFESYKWTDIWAFGLVL
WEIARRTI--VN-GIVEDYRPPFYDVV-PNDPSFEDMKKVVCVDQQTPTI-PNR-LAADP
VLSGLAQMMRECWYPNPSARLTALRIKKTLQKISNSPEKPKVIQ----------------

>AAH71181_1_TGFBR1_protein_Homo_sapiens
PRLLLLVLAAAAAAAAALLPGATALQCFCH--LCTKNFTCVTDGLCFVSVTETTDKVIHN
SMCIEIDLIPRDRPFVC-APSSKTGSVTTTYCC-NQDHCNK---IELPTT----------

-----------GLPLLVQRTIART-IVLQESIGKGRFG-EVWRGKWRGEEVAVKIFSSRE
ERSWFREAEIYQTVMLRHENILGFIAADN-K-DN-GTWTQLWLVSDYHEHGSLFDYL-NR
YTVTVEGMIKLALSTASGLAHLHMEIVGTQ---G-KPAIAHRDLKSKNILVKKNGTCCIA
DLGLAVRHDSATDTIDIAPN-HRVGTKRYMAPEVLDDSINMK-HFESFKRADIYAMGLVF
WEIARRCS--IG-GIHEDYQLPYYDLV-PSDPSVEEMRKVVCEQKLRPNI-PNR-WQSCE
ALRVMAKIMRECWYANGAARLTALRIKKTLSQLSQQEGIKM-------------------

>CAA80258_1_ALK_4_Homo_sapiens
SSFFPLVVLLLAGSGGSGPRGVQALLCACT--SCLQNYTCETDGACMVSIFNLDGMEHHV
RTCIKVELVPAGKPFYC----LSSEDLRNTHCC-YTDYCNRIDLGHLKEPEHPSMWGPVE
---------LVGIIAGPVFLLFLIIIIVFLVINYQRVYHNRQRLDMEDPSCEMCLSKDKT

LQDLSTSGSGSGLPLFVQRTVART-IVLQEIIGKGRFG-EVWRGRWRGGDVAVKIFSSRE
ERSWFREAEIYQTVMLRHENILGFIAADN-K-DN-GTWTQLWLVSDYHEHGSLFDYL-NR
YTVTIEGMIKLALSAASGLAHLHMEIVGTQ---G-KPGIAHRDLKSKNILVKKNGMCAIA
DLGLAVRHDAVTDTIDIAPN-QRVGTKRYMAPEVLDETINMK-HFDSFKCADIYALGLVY
WEIARRCN--SG-GVHEEYQLPYYDLV-PSDPSIEEMRKVVCDQKLRPNI-PNW-WQSYE
ALRVMGKMMRECWYANGAARLTALRIKKTLSQLSVQEDVKI-------------------

>NP_001194_1_bone_morphogenetic_protein_receptor_type_1B_isoform_b_precursor_Homo_sapiens
---------MLLRSAGKLPTPRPKVRCKC-HHHCPENNICSTDGYCFTMIEEDDSLPVVT
SGCL----GLEGSDFQC-RDTPIPHQRRSIECCTERNECNKDLHPTLPPLKNRDFVDGPI
HH-------RALLISVTVCSLLLVLIILFCYFRYRQETRPRYSIGLEQDETYIPPGESLR
DLIEQSSGSGSGLPLLVQRTIAKQ-IQMVKQIGKGRYG-EVWMGKWRGEKVAVKVFFTTE
EASWFRETEIYQTVLMRHENILGFIAADI-K-GT-GSWTQLYLITDYHENGSLYDYL-KS
TTLDAKSMLKLAYSSVSGLCHLHTEIFSTQ---G-KPAIAHRDLKSKNILVKKNGTCCIA
DLGLAVKFISDTNEVDIPPN-TRVGTKRYMPPEVLDESLNRN-HFQSYIMADMYSFGLIL
WEVARRCV--SG-GIVEEYQLPYHDLV-PSDPSYEDMREIVCIKKLRPSF-PNR-WSSDE
CLRQMGKLMTECWAHNPASRLTALRVKKTLAKMSESQDIKL-------------------

>CAA80257_1_ALK_3_Homo_sapiens
LGAYLFIISRVQGQNLDSEDTLPFLKCYC-SGHCPDNNTCITNGHCFAIIEEDDQETTLA
SGC-MKYEGS---DFQC-KDSPKAQLRRTIECC-RTNLCNQYLQPTLPPVVIGPFFDGSI
RW-------LVLLISMAVCIIAMIIFSSCFCYKHSISSRRRYNRDLEQDEAFIPVGESLK
DLIDQSSGSGSGLPLLVQRTIAKQ-IQMVRQVGKGRYG-EVWMGKWRGEKVAVKVFFTTE
EASWFRETEIYQTVLMRHENILGFIAADI-K-GT-GSWTQLYLITDYHENGSLYDFL-KC
ATLDTRALLKLAYSAACGLCHLHTEIYGTQ---G-KPAIAHRDLKSKNILIKKNGSCCIA
DLGLAVKFNSDTNEVDVPLN-TRVGTKRYMAPEVLDESLNKN-HFQPYIMADIYSFGLII
WEMARRCI--TG-GIVEEYQLPYYNMV-PSDPSYEDMREVVCVKRLRPIV-SNR-WNSDE
CLRAVLKLMSECWAHNPASRLTALRIKKTLAKMVESQDVKI-------------------

>NP_001265508_1_activin_receptor_type_2A_isoform_1_precursor_Homo_sapiens
KLAFAVFLISCSSGAILG---RSETECFFNTNQTG-VEPCKR--HCFATWKNISGIEIVK
QGCW-LDDINCYDRTDC--VEKKDSPEVYFCCC-EGNMCNEKFSPEMEVTQPTSNPVTPK
PPYYN----ILLYSLVPLMLIAGIVICAFWVYRHHKMAYPPVLVPTQDP-----------
-----------GPPPPSPLLGLKP-LQLLEVKARGRFG-CVWKAQLLNEYVAVKIFPIQD
KQSWQNEYEVYSLPGMKHENILQFIGAEK-RGTS--VDVDLWLITAFHEKGSLSDFL-KA
NVVSWNELCHIAETMARGLAYLHEDIPGLKD--GHKPAISHRDIKSKNVLLKNNLTACIA
DFGLALKFEAGKSAGDTHGQ---VGTRRYMAPEVLEGAINFQ--RDAFLRIDMYAMGLVL
WELASRCT--AADGPVDEYMLPFEEEI-GQHPSLEDMQEVVVHKKKRPVL-RDY-WQKHA
GMAMLCETIEECWDHDAEARLSAGCVGERITQMQRLTNIITTEDIVTVVTMVTNVDFPPK
ESSL
>NP_001097_2_activin_receptor_type_2B_precursor_Homo_sapiens
WVALALLWGSLCAGSGRG--EAETRECIYYRTNQSGLERCDKRLHCYASWRNSSGELVK-
KGCWLDDFNC-YDRQEC---ATEENPQVYFCCC-EGNFCNERFT-HLPEAGGPEVTYEPP
PT-----APVLAYSLLPIGGLSLIVLLAFWMYRHRKPPYGHVDIHEDP------------
-----------GPPPPSPLVGLKP-LQLLEIKARGRFG-CVWKAQLMNDFVAVKIFPLQD
KQSWQSEREIFSTPGMKHENLLQFIAAEK-R-GS-NLEVELWLITAFHDKGSLTDYL-KG
NIITWNELCHVAETMSRGLSYLHEDVPWCRGEGH-KPSIAHRDFKSKNVLLKSDLTAVLA
DFGLAVRFEPGKPPGDTHGQ---VGTRRYMAPEVLEGAINFQ--RDAFLRIDMYAMGLVL
WELVSRCK--AADGPVDEYMLPFEEEI-GQHPSLEELQEVVVHKKMRPTI-KDH-WLKHP
GLAQLCVTIEECWDHDAEARLSAGCVEERVSLIRRSVNGTTSDCLVSLVTSVTNVDLPPK
ESSI
>NP_003233_4_TGF_beta_receptor_type_2_isoform_B_precursor_Homo_sapiens
RGLWPLHIVLWTRIASTINGAVKFPLCFCDNQKSC-MSNCCEQEVCVAVWRKNDENITLE
TVCHHDFILEDAASPKCIMKEKKKPGETFFMCSCSSDECNDNIIEEYNTSNPDLLLVIFQ
---------VTGISLLPPLGVAISVIIIFYCYRVNRQQKLSSTWETGKTRKLMEFSEHCA
IILEDRSDISSTCANNINHNTELLPIELDTLVGKGRFA-EVYKAKLKQNTVAVKIFPYEE
YASWKTEKDIFSDINLKHENILQFLTAEE-RKTE--LGKQYWLITAFHAKGNLQEYL-TR
HVISWEDLRKLGSSLARGIAHLHSDPCGRP-----KMPIVHRDLKSSNILVKNDLTCCLC
DFGLSLRLDPTLSVDDLANS-GQVGTARYMAPEVLESRMNLE-NVESFKQTDVYSMALVL
WEMTSRCN--AV-GEVKDYEPPFGSKV-REHPCVESMKDNVLRDRGRPEI-PSF-WLNHQ
GIQMVCETLTECWDHDPEARLTAQCVAERFSELEHLDRLSGRSCSEEKIPEDGSLNTTK-

>NP_065434_1_anti_Muellerian_hormone_type_2_receptor_isoform_1_precursor_Homo_sapiens
LGLWALLPTTCVFFEAPGTELPRAIRCY---------SRC-----CFGIWNLTQDAQVEM
QGC-RDSDEPGCESLHCSPRAHPSPGSTLFTCSCGTDFCNANY-SHLPPPGSPGTPGSQG
PQAAPGESIWMALVLLGLFLLLLLLLGSIILALLQRKNYRVRGEPVPEPRPDS-------
-----------GRDWSVELQELPE-LCFSQVIREGGHA-VVWAGQLQGKLVAIKAFPPRS
VAQFQAERALYELPGLQHDHIVRFITASRGGPGR--LLSGPLLVLELHPKGSLCHYL-TQ
YTSDWGSSLRMALSLAQGLAFLHEEQNGQY-----KPGIAHRDLSSQNVLIREDGSCAIG
DLGLALVLPAWTPTQPQGPA-AIAGTQRYMAPELLDKTLDLQDWGMALRRADIYSLALLL
WEILSRCPDLRPDSSPPPFQLAYEAEL-GNTPTSDELWALAVQERRRPYI-PST-WRCAT
DPDGLRELLEDCWDADPEARLTAECVQQRLAALAHPPRGCPPLCPEDCTSIPAPTILPCR
PQRS
>AAA28996_1_thick_veins_protein_Drosophila_melanogaster
LLVFGVNVVLVHLTTGEFVENARSLTCYC-DGSCPDNGTCRPGGSCFSAVQQLYDEEERT

```
YGCMPEDNGG---FLMC---AVPHLHGKNIVCCDKEDFCNRDLYPTYTPKLTTPAPDLPV
SSESLHT--LAVFGSIIISLSVFMLIVASLCFTYKRREKLRKQPRLINSMCNSQLSPLSQ
LVEQ-SSGSGSGLPLLVQRTIAKQ-IQMVRLVGKGRYG-EVWLAKWRDERVAVKTFFTTE
EASWFRETEIYQTVLMRHDNILGFIAADI-K-GN-GSWTQMLLITDYHEMGSLHDYL-SM
SVINPQKLQLLAFSLASGLAHLHDEIFGTP---G-KPAIAHRDIKSKNILVKRNGQCAIA
DFGLAVKYNSELDVIHIAQN-PRVGTRRYMAPEVLSQQLDPK-QFEEFKRADMYSVGLVL
WEMTRRCYTPVSGTTCEDYALPYHDVV-PSDPTFEDMHAVVCVKGFRPPI-PSR-WQEDD
VLATVSKIMQECWHPNPTVRLTALRVKKTLGRLETDCLIDVPIKIV-------------
----
>AAA53242_1_SAX_Drosophila_melanogaster
NIYLVFLLTFLLYNNARAVQTHPRYKCSC-EPPCRDTHTCQNAIQCWKSRTRHADQVQES
RGCSTSPDQL---PMICINGPSKRNTGKNVVCC-AGDYCNEGDFPELLPFDSNDVTVITA
DTSSISK--MLVAVLGPFLVIALLGAVTIFFIRRRKRLAASRTKQDPEAYLVNDELLRAT
SAGDVTSGSGSGLPLLVQRTLAKQ-VTLIECIGRGKYG-EVWRGHWHGESIAVKIFFSRD
EESWKRETEIYSTILLRHENILGFIGSDM-T-SR-NSCTQLWLMTHYYPLGSLFDHL-NR
NALSHNDMVWICLSIANGLVHLHTEIFGKQ---G-KPAMAHRDLKSKNILVTSNGSCVIA
DFGLAVTHSHVTGQLDLGNN-PKVGTKRYMAPEVLDESIDLE-CFEALRRTDIYAFGLVL
WEVCRRTI--SC-GIAEEYKVPFYDVV-PMDPSFEDMRKVVCIDNYRPSI-PNR-WSSDS
LMTGMSKLMKECWHQNPDVRLPALRIKKTIHKLASADEKIRLDFDEVCV-----------
----
>NP_001262574_1_punt_isoform_C_Drosophila_melanogaster
QLTLVCCLIGIHGSILPGEHFDEK-MCT--TQQCETIEHCMEADSCYVLWSVNETLRIKM
KGCF--TDMHECNQTECVTSAEPRQGNIHFCCC-KGSRCNSNQKTTQVPKEKTQDGSNLI
---------YIYIGTSVFSVLMVIVGMGLLLYR-RRKQAHFNEIPTHEAEITN-------
-----------SSPLLSNRP-----IQLLEQKASGRFG-DVWQAKLNNQDVAVKIFRMQE
KESWTTEHDIYKLPRMRHPNILEFLGVEKHM-----DKPEYWLISTYQHNGSLCDYL-KS
HTISWPELCRIAESMANGLAHLHEEIPASKTD-GLKPSIAHRDFKSKNVLLKSDLTACIA
DFGLAMIFQPGKPCGDTHGQ---VGTRRYMAPEVLEGAINFN--RDAFLRIDVYACGLVL
WEMVSRCD--FA-GPVGEFQLPFEAEL-GLRPSLDEVQESVVMKKLRPRL-LNS-WRAHP
GLNVFCDTMEECWDHDAEARLSSSCVMERFAQLNKYPSTQLLIKNHTNIDDAKESTNCL-
----
>AAL16073_1_wishful_thinking_Drosophila_melanogaster
WAIYLLLALISLGRATPVHDDDGDQDSSGESTPIPSRRTCPDGYFCFTIWNQTANRVVK-
QGCWNTDRTSICSQSECSSAPTSKTSSLYYCCC-SGGVCNAQYSVVEPAPLELGSNEGRT
SITNRATEKTMLGLAGGLTALTIGIFLAVQYCRTAKEKPEPEESPLAPS-----------
-----------GPGYSSNLRNVDN-MNLIGMLGSGKYG-TVMKGLLHDQEVAVKIYPEEH
HQYYVNERNIYALPLMECPALLSYFGYDE-R-CTMDGRMEYQLVLSLAPLGCLQDWL-IA
NTLTFSECCGMLRSITRGISHLHTERLGDQH----KPCVAHRDINTRNVLVQADLSCCIA
DFGFALKVFEYKGEVAMAETKSIVGTLRYMAPELLEGAVNLRDCETSLKQMDVYALGLVL
WEVATRCSDFYAPQATPPYKAPYEQEV-GSHPSFDQMQALVVRHKARPLF-PTG-WGGGA
AAKVVRDTCEDCWDHDADARLTSLCAEERMQEMSTLPQENSLSCLEHDVSVEELIASHQH
QQQK
>CAH1249916_1_ACVR1_Branchiostoma_lanceolatum
QACLQTFYRLLGLKSARIDSGVPPVECWGGEGEGAEPESCCRQ-ECYSAIVYGDL----K
KGCFGQEEQS---RMSC---NTPNQRTVNVVCC-DYEMCNKNITPTLPTDPDPTFAVDNE
MTQ------LVIYAVSPIGALIILAAAAICIARFHKRRLQRYQRDVERGGVHVGDMHAYP
AGDNQSSGSGSGLPFLVQRTVARQ-ITLVEQIGKGRYG-EVWRGQWQGENVAVKIFNSRD
EKSWFRETEIYNTVLLRHENILGFIASDM---TSRNSCTQLWLITHYHEFGSLYDFL-QR
TTLDAHRMMKLACSIAGGLVHLHVEIFGTQ---G-KPAIAHRDLKSKNILVKSNGQCCIA
DLGLAVMHSQATDTLDLGSN-PRVGTKRYMAPELLEETMNFE-CFDSYKRIDVYALGLVL
WEVARRCT--SG-GIAEEYKPPYYDVV-PSDPSYEDMKKVVVVDQQRPSI-PNR-WSSDQ
TLTAMAKLMRECWYHNPAARLTALRVKKTLNKLQNSLEKLKELKQDV------------
----
>CAH1241670_1_ACVR2B_Branchiostoma_lanceolatum
VRVWLRLAILLYAAGVSLTCEFFDNECH--PENCNAFVMCQDEGHCYALWKNESNITLVK
KGCW-LDDNDCYDKSECYEHEEQPENGMFYCCC-EGNLCNQHFDIPQPDRSTAETPRTIP
IDNNKELFRSLMYSLFPICGIALVVAVTFYMY--KRHKSEHIQIPTQD-----------
-----------PLPPPPSPLLGLRPLQLLEVKARGRFG-AVWKAQLLNEYVAVKIFPLQD
KASWLNEQDIFTTQLMKHDNLLQFIAVEK-R-GD-GLEMELWLITDFHERGSLTDYL-KG
HLLKWSEMCHIAETMARGLAYLHDDVSGRNGEY--KPAIAHRDFKSKNVLLKQDLTVVVA
DFGLAMKFDPGRSCGDTHGQ---VGTRRYMAPEVLEGAINFN--RDAFLRIDMYAFGLVL
WEIVSRCS--AVDGPVDEYQLPFEEEI-GQHPTLEDMQEVVVHKKMRPQF-RET-WLKHQ
GLEILCETIEECWDHDAEARLSAGCVEERLGQLSRTVNITTSNTMDITTNQQTPPKESSL
----
>CAH1249913_1_TGFBR1_Branchiostoma_lanceolatum
RVLLPFLGLFLAVSWAPA----AALQCACD--RCEKNFTCETDGVCFKQLILESS-VTMT
MRCMADRLTPPENPFIC---QDSNNPRFAIRCCKTHDFCNQELNVVLPASTTQVEDRESP
GGSAGLGTVLAAIIAGPICLACIVFMIVLCLCQK-----RGARMPLSDPEESMDSDNPLM
PPGHTTSGSGSGLPLLVQRTIARQ-ITLMESIGKGRFG-EVWRGRWRGENVAVKIFSSRE
ERSWFREAEIYQTVMLRHENILGFIAADN-K-DN-GTWTQLWLVSDYHEFGSLFDFL-NQ
RAVSAHGMIKLALSTANGLAHLHMEILGTQ---G-KPAIAHRDLKSKNILVKKNGQCAIA
DLGLAVKHDPITDSVDIAPN-NRVGTKRYMAPEVLDDTINIT-HFDSFKRADVYALGLVF
WEIARRCN--VG-GIYEEYQLPYFDMV-PSDPSIEEMRKVVCDLRQRPSV-PNR-WHENE
YLRVMSKVMKECWYHNGAARLTALRIKKTLAQLAPYDEIKLV-----------------
----
```

>CAH1241290_1_TGFBR2_Branchiostoma_lanceolatum
WAATSCRAC---------VCDDVTGRC---DVRCDYVRTCGEGDGCFASMYLMEDTYVVD
TGCWAGSAFNAPQADDC---RADDSFPGMYTCLCGTDQCNGEMIPNDPPSPPVGSGGFPW
PGLDDPTVRVLIGIVAIIPALLVLVLVIFAAYVLYRRRHPKNPPSSIFGAEDGNGNYQIK
LDDFDSEMADPSLENTLNHNEEHLPIKLDTSVGKGRFA-EVWKGKLFQETVAVKIFRYDE
KQSWQREKDIFSDPALKHPNILDFLTAEE-R-GN-GIDRQYWLITSYHSNGCLRAYL-GR
HILTWEQLCGMSQTTAAGLAYLHADTDGAA-----KMPIAHRDVKSSNVLVKSDGTCCLA
DFGLALNLDPTIRVEEYANS-GQVGTPRYMAPEALESRVNLV-NLESFKQIDVYAMALVL
WEMLARCE--VI-GDVGPYQPAFADKLSGDHPGIDTMKDVVIRGRERPDI-PTS-WLAHQ
GLCRMSEVIEDCWDHDGEARITASCAEERINELQKRITIDSEDEEKEPLRPKKRYIEETD
V---
>CAH1246056_1_BMPR1B_Branchiostoma_lanceolatum
WRLGVFLAVWCSLIGLLSTTEGRSIKCYC-SNHCPENGTCITNGRCFMQVESDLAGQIVS
HGC-LSPEGP---LLQC--KAQVHSIPRSIQCCMDSDMCNLQLNATLPPSANDTPGLPPN
AYYDPSIHHITLVISVTVCTTVLMLTIAFVYFRYKRELGTRYDMEVGDH------DETFI
PPGDQSSGSGSGLPLLVQRTIAKQ-IQMLHSIGKGRYG-EVWKGKWRGEYVAIKVFFTTE
EASWFRETEIYQTVLMRHENILGFIAADI-K-GT-GSWTQLYLITDYHENGSLYDYL-RG
NTLDRAGLLRLAYSAACGLSHLHTEIFGTQ---G-KPAIAHRDIKSKNILVKKNGACCIA
DMGLAVRFISETNEVDIAPN-TRVGTKRYMAPEVLEETMNKN-SFDAYKMADIYSFGLVL
WEIARRCI--VG-GIVEEYQLPYYDCA-PHDPSYEDMRRVVCIERQRPSI-PNR-WHTDE
VLRPMAKLMSECWAHNPNARLTALRVKKTISKMMVEAQIKA------------------

>CAH1242226_1_BMPR2_Branchiostoma_lanceolatum
-----------------------MC---------------------RGTEA-----

----------G-------------------AEERIG---------------------
--------------ME------------------GTVEYLLVMDYAHHGSLYHYL-KT
YMVNWTGMCRLGHSLARGLAHLHSDVKGGES----KPAVAHRDLTSRNILVKADGSCALS
DLGFAMRLTCNPGQEDTAHI-TEVGTVRYMAPEVLEGAVNLRDFESALKQVDMYAVGLII
WEIATRCQDLYMGQPVPEYMQPFQAEL-GQHPTFEELQVIVSREKRRPLL-PDA-WRENS
AVKSLKETIEDCWDQDAEARLTALCVEERMAELMSMPSLLLEMESTNSLTPSTTTTTLSE
HTNT
>NP_495271_1_sma_6_Caenorhabditis_elegans
NITFIFILIFGFFNTQKCPNDILKNRCYCNDQSICGNMTCQDGAACYHAVEEVYNTLHK-
WGCATLERGSGASHLTC---A--HHSPKSIGCCYEGNYCNKNLIVHHHKEKALQEKTDNP
EDYDSPLENMFIMVFATVMSVFAVIGCIYLCITRKSKARARAKTVSLKT------ESTYM
ES-----GSGSGQAALIQRTVRQD-LTIIKTIGQGRYG-EVRKALYRGSYVAVKTFYTTD
EDSWKNERDVYQTNMINHENILQFVAADIWS-EE-DSMTKMLLITDYHELGSLSDYLCRE
ETLTTDEALRLIHSCICGIEHLHAAVHGTGSF-R-KPEIAHRDIKSKNIIVKRPNVCCIA
DLGLALRY-QNDKILPEKFN-VQVGTKRYMAPELISNKLNPK-DFSQFKMADIYSMALVM
WEVAIRCEEVLTVGNISQHVPPFDGIV-HNDPNFDEMNDVICVRRIRPP--PDLAWKNVP
ALNELSKLMEDSWHSIPHFRHSALKLKKEMAELIKNPDRQNQSQRKVEFQQQDSGLVESA
TNQS
>NP_001023160_1_Cell_surface_receptor_daf_1_Caenorhabditis_elegans
IRHVVFCLLALVYGAETSVNFTRLHLCHCSREVGCNENTCYTDGSCYQSARPSPE--ISH
FGCMDETEFHDTAAKVC---TNNTKDPHVWICCDKGNFCANETIIHLAPGPQQSSTWLIL
---------TILALLTFIVLLGIAIFLWYIRFKPGSSVPEVAPIEQQGSTMSTSAGNSFP
PGIMETSGSGMGPTTLHKLTIGGQ-IRLTGRVGSGRFG-NVSRGDYRGEAVAVKVFNALD
EPAFHKETEIFETRMLRHPNVLRYIGSDR-V-DT-GFVTELWLVTEYHPSGSLHDFL-LE
NTVNIETYYNLMRSTASGLAFLHNQIGGSKESN--KPAMAHRDIKSKNIMVKNDLTCAIG
DLGLSLSK-PEDAASDIIAN-ENCGT-RYLAPEILNSTMQFT-VFESYQCADVYSFSLVM
WETLCRCE--DG-DVLPATVIPYIEWT-DRDPQDAQMFDVVCTRRLRPTE-NPL-WKDHP
EMKHIMEIIKTCWNGNPSARFTSYICRKRMDERQQLPQKPKDESPANGAPRIVQKEIDRE
DEQE
>NP_498211_1_Cell_surface_receptor_daf_4_Caenorhabditis_elegans
LKALVLICLPLFLIATPVEDEVISIECYYDEMECEKKKTCLKAVGCLAVFQEINSPQYKS
LGCMPYQHAD---SMNCCRQGRSFRGGIGMCCC-STNNCNMPDLEMVNPSLKKDSDNSAL
LWASTPSNMIIIALSVILCIALLILAYVGWKFQQEIKKQQKIKFDMEKTDALEAGNVPLV
EPEE-------EMIEMVETPKELP-ITDFQLISKGRFG-KVFKAQYTPDLVAVKKLNEFQ
KASFLAEKRIFDELNPKYKSIVEFVCAEK-------IGDEYWIVTEFHERLSLYELL-KN
NVISITSANRIIMSMIDGLQFLHDDFFGHP-----KKPIIHRDIKSKNILVKSDMTTCIA
DFGLARIYSYDIEQSDLLGQ---VGTKRYMSPEMLEGATEFT--PTAFKAMDVYSMGLVM
WEVISRTKLHQT-DEPPNYQMPF-QVI-GFDPTIGLMRNYVVSKKERPQW-RDE-IIKHE
YMSLLKKVTEEMWDPEACARITAGCAFARVWNHIMSPDNAEPEPEELPDLPIVEKIYDIA
TNML
>KAJ8035571_1_TGF_beta_receptor_type_2_Holothuria_leucospilota
-----------------------MC--------------NQQLFYATALTIPPEVFT
KGYY---------------NVSPSNDN----STRRNRSSNLEEPFDPLPDREEASTPRT
PENKEPVFIAVILVVTIACVAIATSTIFFLLIKLRQRADGMCTVTSNTDSTSLDIGTEYS
DTKAVDGGTPPQSPNLYENSRLN--LHLDEVIGRGRFG-AVWRAELKTETVAVKVFYEFD
STSWNVEKELFNDPALKHENIIRFMKAEVRQITYPQPQRQYWLISQYYPGGSLQDYL-SE
NTVTWDEFCRMACSTAKGVAHLHADVIGEDPPMN-KYPVAHRDLKSSNVLIKDDGSCVIA
DFGLALKLDPRASEQELANS-GQVGTPRYMPPEALESKINLQ-NIESFKQIDVYSLSLII
WEIARRCT--VL-PDVPDYELPYTEQLHGCHPTIDEMRAIVAKRQERPVI-PET-WQGIN

GMSIVANTIIECWDEDPEARLTASCIATRFSQFHEWKDGEHLASPGDLHPTTII------

>KAJ8022909_1_Bone_morphogenetic_protein_receptor_type_1B_Holothuria_leucospilota
-----MIILLLIQNAMANSANEQGLMCYC-QEHCPNNNTCFVGGWCFKSIQTGEEEPIVT
LGCLPEEDGG---LMLC--KAKKHDIPRTMKCCNDRPFCNIYLNTTLPPTTTTSIPGGIP
HGSSEPHMLIVLLFSVTVFAAAFILILTFFYLRYKRVFRRRFDLETREE------DESFI
GPGEQTSGSGSGLPLLVQRTIAKQ-VQLIRCVGKGKFG-EVWKARWRGENVAVKIYQNTE
EDSWFRETEIYQTVLMRHENIMRFVAADI-R-GT-GTYTQLYLITEYHEYGSLYNFL-RT
NVLHSSVAVRLAFSAASGLTHLHTEICGMK---G-KPAIAHRDITSANILVRHDGQCVIG
DLALAARYFSDTDEIDLPPS-KRLGTRRYLAPEILDETMATN-SFEAYKLVDIYAFGLVL
WEIARRCV--TKAGIVEECWLPYYDCV-PSDPSFEDMRRVVAFERKRPSI-PNR-WCGDE
VLSSIAKIISECWHPNPAARLTSLRVKKSLAKLQNTDHHKV-------------------

>KAJ8036284_1_Activin_receptor_type_2A_Holothuria_leucospilota
NVTTVIGIAIAVVSVLAVADGPTTTKCFYNNKSCKNTEECNEMTACFAVWKNTSDFVMQN
QGCW-DNGNDCLDRTQC-ISDNTVTDNTFFCCC-IGDLCNGEVSTDSPQSTKGSGSNPTP
VLTGDPLICTIIYSLVPISIFTIVTIVIYWMCR-RYHYTSLVSIQTSEPSPL--------
-----------TTPLTPTATDFQP-LQLIEVKARGRFG-AVWKAQMNSRYVAVKVFPLQD
RQSWISEQEIYSLPNMQHENILQFLGTEK-R-GE-GLDTAFWLITEYHQRGSLYDYL-KA
NLVSWNNLCHIAESMARGLAFLHEP-CGA------KPPIAHRDFKSKNVLLMSNTTACIA
DFGLAVKFDPGKKLCENHGQV--VGTRRYMAPEVLEGAIQFN--QDAFMRLDMYACGLVI
WELVSRCS--AQDGPVDEYMLPFEEEV-GQAPSLEEMIEAVVSHRKRPAF-RDT-WMKHS
GLASLCETIEECWDHEAEARLTAGCVEARLSQLLRTSNQVITTTDLNHTNSSTTPLITDL
SNGS
>KAJ8036516_1_Bone_morphogenetic_protein_receptor_type_2_Holothuria_leucospilota
ALLFVLLSVYCVSFVGTE------TLCYSSTKNVPGTIRCGDKYKCFAAWSQVNDELLW-
QGCWSKAKNNQCIQSNCRYRGANKTDRIYFCCC-EGNYCNVNVTYIKPPQPSTLPPKPLP
TQGHSVVEMAILLSVVTFTGISILVIISVLFCRVCFRKPENSGQPLPY------------
-----------IEPSPVEPDFDMNDLKILELIGQGRYC-NVYKGVLNNREVAVKIFAGAS
RECYNNELDIYGIALLEHDNVLKFIGSGERKAED--GWQEYMLVTELIHCGSLMSYL-KE
YTYDWHAMCKLAQTAAAGLAHLH--LAATKEGEH-KPAIVHRDINSRNILVRTDGTCCIG
DFGFAMKVCVRDGNRDNSNI-TDVGTVRYMAPEVLDGAVNLRDCECALKQVDIYSLGLVL
WELATRCKDLYP-GTVPSYKLPFQDEV-GLHPSFAEMQVLVCTDRGRPAF-PDE-WKDHM
ALRSLKDTIEECWDHDAEARLTALCVEERIIELMVLQDGLSHQEASSVSEPTISPSQGLI
VNEM
>KAJ8019424_1_Activin_receptor_type_1_Holothuria_leucospilota
RTIFSTFLLFLVGIAFGQSNITESFLCGCDQISCDYEPTCQTNLKCYIYLTTDVDRIYR-
KGCLTDGDQV---IMIC---------RNEVKCC-SGELCNNNITLAVPTTQSPQPSSSTD
-------YSMVLAVIFCIILFFGSIAISLCLYHRKHEMHRLAPLDMEYGPST---DLRAV
PAGDNTSGSGSGSGLPYLVQRTLARQ-IVLVQQVGKGRYG-EVWRGHWQGENVAVKIFSSID
EKSWFRESEIYNTVMLRHDHILGFIASDM-T-SR-NSCTQLWLITHYHELGSLYDYL-NR
NVIDAPLACRLALSAASGLVHLHTEITCNNS----KPAIAHRDIKSKNILVKRDLRCCIA
DLGLAVLHTQENGCIDMGVN-NRVGTKRYMAPELLDETMNIT-CFESFKRVDIYAFGLVL
WEIATRCQ--VG-GMVEDYQPPFHDKV-PSDPSFEDMRKVVCVDQCRPTI-PNR-WATDP
TLTNLAKLMRECWCQAASSRHTSLRVKKTLIKISESCQKSSKLDMENDRIIV--------

>KAJ8019706_1_TGF_beta_receptor_type_1_Holothuria_leucospilota
VASISILFLLVKGVALQEEDGQGWRKCRCNSDLCRQDSTCAANAYCLATKVIEKGILV--
LKCENHVYGIVDHPISC---STIRGSFT-AACCNDTDYCNEKLDPKVPPTPPPVHKCEQP
GHDPGSRDPMATLIASPVCVICIAVTLYVILQHKCRKMPMYLPTDIQDPSYPHHHDCGPN
VLVEEVADAPQGLPLLVQRTIARQ-IILLDTIGKGRYG-EVYKGRWRGEYVAVKIFSSRE
ERSWFRETEIYQTVMLRHANILGFIAADN-K-DN-GLCTQLLLITDYHERGSLFDYL-DR
NTVDIQGMLTLALSLATGLAHLHMEIVGMQ---G-KPAIAHRDLKSKNVLVKGNGQCAIA
DLGLAVRHISATDTVDIAQN-NRIGTKRYMAPEVLNDTLNRN-HFDSFKRADIYSLGLIL
WEIARRCC--VG-GIYEEYHLPYYDMV-PTDPSLDEMRRVVCLENRRPSI-PNR-WNSYE
ELRVIAKIMKECWYANGGARLTALRIKKTLATLHPLEDIKG-------------------

>XP_065656268_1_activin_receptor_type_1_isoform_X1_Hydra_vulgaris
KVISEFPAMNLFGLTSKDLNKFSEHSCFCD--TCE-NKMCFTKSGCFSTITYGTKLVGKS
NGCFQDDSKSVHSLLSCSTGSAYTAQCYHTSCC-RYNLCNENVYLTKPHEEYLKIPTSAP
PGNNFPST-LMISIVAPFAAVIILLLILFKVWKRKNFHIKIKKSLLPYSKSEKVADDVFK
QLFDISSGSGSGSGLPFLVQRTVARQ-IILVDLIGKGRYG-EVWRGDWHGESVAVKIFNSRD
EDSWKRETQIYNTVMLRHDNILGYIASDI-AIKK--DETCMWLIAHYHEHGSLYDYL-NT
HSLSVYEMALISHSAVCGLVHLHTEINGMQ---G-KPAIAHRDIKTKNILVKSNGQCCIS
DLGLAVLHSKESNCLDVSCN-RRVGTKRYMAPELLTETMCMT-SFDAYKHADMYAFGLVL
WEIARRTE--FQ-GTVEEYQPPYYDYL-PQDPSFEEVKKVVVDDHYRPDI-PKH-WLNDE
DALKFSKVMRECWREEPTARLSALRVKKSLSKLLPALPLEKKEFYIYNPSQYAGLTIDRD
KLSS
>XP_047137189_1_bone_morphogenetic_protein_receptor_type_2_isoform_X1_Hydra_vulgaris
SLLFLVNCLFCRFQFIVLKLDNNFLNCYFKYIRCPNGSFC----LAYALFSYSYGVNILK
QGCFENVNLDKCSNNQC---AIYENEESKFCCC-SGSLCNMPVLPNKNFSVSAKYSGSSL
HGVAQVISISLLLVLVSVMASVFVIAVVIYNWKFSSKPVSNKKKFMDNNLQNQ-------
-----------QVQTINEELSNLDDYIHGYIVHNGNHT-TVRLATYKGSTVVVKVYPPRF
TAQLVNENEIYCLL-PRHSNISNLIRTSF-V-QN-G-----YLLLTFYPQGSLFNYL-RS
NTLTWNQLFVMITSISNGLSFLHNEVRPTRPVLPDRPVIVHQDLNSRNILLKDNGECVLA

```
DFKFSLRL-GKSKHGENFSL---IGSAVYAAPEVLLQSFIPRNWSDSLKKVDIYALAMVM
WELCKRCPTFYKLAVVAESTLPYLEEL-GIHPCIQKLRNFVCVDNKRPAF-PIE-WNYDP
IVEMLKTTITQCWDRNPSVRLSSSCILNRVKYFHEKKLKECHLSYNSTNKQNIPEFFDVS
----
>XP_065643772_1_bone_morphogenetic_protein_receptor_type_1A_isoform_X2_Hydra_vulgaris
QLSFLFFYSLLYQKILFG------LECFC-SSDCPDNETCYTSYKCFAAVEYLEGTKVHQ
YGCTPKFEGG---IFQCRQKHIQHEVPMVFKCCDDQPRCNENLILEIPIEEQQKYEAVFK
EHKIFQRRQVGLVVSVLLFILCFSLFSSYFYLSAKIFKKSNNSSEVKLSPVDKSENLTC-
-----NSGFNGFKSRLVQRSFARD-ISIVKEVGRGCFS-VVYIGLYQSQTLAVKIVDESD
EYSWQREQHFYTTRNLGHENILGFIGADI-V-ER-GTVIRV-LVTQYHPFGSLYNFL-RI
HSYDFKIFFRLLYSSVCGICYLHNSISGLY---G-KPAIAHRDISSKNILVKDNLECCIS
DFALAVE--ENPDEFNLVPSKAKLPNPRYMAPEVLENPSCRNQTVFFYQKADMYSFSLVM
WEILLRYE--IK-GVAEPYRLPFEEYV-GFYPSINQMNDIINVKKLKPVI-AKK-HILSS
SAQSIIKSMNESMKYSAMSRSSSCRLKTNLNKYMQKIAKLTETETGGSNNKR--------
----
>XP_002158603_3_bone_morphogenetic_protein_receptor_type_1B_Hydra_vulgaris
ILFFLICNYILHYLQVLA------LECFC-SFHCPHNETCNTNYKCFATLEYLEGTKEYR
YGCMPEFEGG---IFQCRQKHIQHAVPMVLKCCNDQPRCNENLILEIPIEEQQRYEESFN
KHHVFQSQQIALVVSVTLSIIFFAMFAAYIYLRYKKQKKSNCDRQVMLPPDEKTENSTC-
--------STGLKSKLAQRSFARE-ISIVKEVGRGRFS-VVYIGLYQDQVLAVKIVDESD
EYSWQREQHFYKTGYLCHENILSFIGADI-V-ER-NTVARM-LITQYHPFGSLCTFL-QN
HSFDFSIFFRLVYSSVCGIRYLHNPISGSH---G-KPAIAHRDISSRNILVKDNLQCCIS
DLALAVE--ENAGESKLISSKAKLPNPRYMAPEVLENPYSTNQTVSFYQKADMYSFSLVM
WEILLRYE--IE-GVAEPYRLPFQEYV-GLNPSINEMTDIINVKKLTPEI-PKK-YILDQ
NARNIIKSMKNSMKYSAMSRFSSYRLKKSLNKCMQEKTKLTEIGDTSNNTCSTTVT----
----
>XP_002155736_4_bone_morphogenetic_protein_receptor_type_1B_isoform_X1_Hydra_vulgaris
QLSFLFFYSLLYQKILFG------LECFC-NSDCPVNETCYTSYKCFAAIEYLEGTQVYQ
YGCTPEFEGG---IFQCRQKHIPHQVPMVFKCCDDQPRCNENLTLEIPIEEQQKYEKNLK
RRNMVQSQQVGLVVSVLLILLVFSLYSTHFYLSTENLKKPNNSSNVKSPDDKSENSTCNS
GFTGFNSGFTGFKSRLVQRSFAKD-ISMVKVVGRGRFS-VVYIGLYQDQTLAVKIVDESD
EYSWQREQHFYKNQNLGHENILGFIGADI-V-ER--DTVDRFLITQYHPFGSLYNFL-QN
HSFDLPIFFHLLYSSVCGICYLHDPISGFH---G-KPAIAHRDISSKNILVKDNLQCCIS
DFALAVE--ENPGEFDLVPSKAKIPNPRYMAPEVLENPSSCNKSVFFYQKADMYSFSLVM
WEILLRYE--IK-GVAEPYRLPFEEYV-GFNPSINQMNDIVNVKKLKPVI-AKK-YILDS
KVRSIIKSMQRTMLHSALSRSSSRRLKIDLYKCMQEIAKLTEIGGSNNNS----------
----
>XP_047138021_1_activin_receptor_type_1C_isoform_X1_Hydra_vulgaris
KVVLGILIVFAYVGIKPFAFEKMA-QCYCNPHRKCVNNICLEETVCYASLEKDYS---IE
KGCT---------PSLCYKEKQTPVHDRVFECC-ETNLCNFNLTKYFKMYEKDTQSTVLT
AKQIESKPSVAISIAIPLSTLFIICSIIALYVHKKKRAIRLIQGKTRQRLLQEEMDLNEL
SLVDTSSGSGAGLPLLVQRTIARQ-IILHECIGRGGFG-DVYRGTWNEQDVAVKIFSTNE
EASWFREYQIYQTTMLRHENILGFIAADS-K-DT-GACTQLWLVSEFHKLGSLFDFL-HT
NTVSLEELFIMAISIVNGLAHLHTEVIGTQ---G-KPAMAHRDMKSKNILVKNNKTCCIA
DLGLSVLHTSFNDKVDMPNT-SKIGTVRYQPPEILCGSFNVQ-NFESYRQADIYSLGLCF
WEICRRTD--IGTGKIHSYELPYFDAV-NSDPSFEEMNDVVCRQNKRPLF-DES-WHQDE
KLKAMVKLLSECWYNDSAARLSALRIKKTLLTLSKDICTNIKQK----------------
----
>XP_012562347_1_activin_receptor_type_2A_isoform_X1_Hydra_vulgaris
GCMFLLLISRVFAYSVNRAVESQSSLCYKDESKCD-EEICIEMSQCQAIYRNISGLRHWF
FSCFHGDQECGTDTCPL---HYVPSHDLWYCCC-TTSLCNTRIYPTLNILTENASTTIAA
HKNFTTEGSILVYILVPLSVVIVMIGLFACIKWKYKKKE---------------------
-----------DILLVYERDSSK--YEYLNIIHQGQFT-KLWLVQHQDQKMVVKLLLPTL
QNLWTNEREIFVKYNLKHRNIIKFLAAET---ENINNKLHYLLVVDYYEKGSLVDYL-KK
NVITVQQMLKFIFSIVRGLVYLH--MPESK-----KPRIAHRDLKSANILITNDLECVIC
DFGLAIAV-DKNNFQTCFDK-AQVGTKRYMAPEILDGAIVFN--SESYFLMDIYSTALII
WELVSRCN--YESEVIKHYQPPYFDQV-GENPSIWQMKDCVVDRNVRPPI-PLE-WRNNT
ILNMICITMEECWDRDPEARVSANCIHERLLAIKDQTSTEIALHMQEETVKTLLLGHNL-
----
>XP_012557412_2_activin_receptor_type_2A_isoform_X1_Hydra_vulgaris
CIKRLICITFCILHSYPRLNNCSD-QC-CVENHCVNLTTCCVKNVCFVLFNKKNGFEPFL
SGC-GDFCDA---KAQC--KTENRGDEISYMCCCNGNLCNKHSVVSIQPTPNTTSNVIQI
NYKSFQKKSFIGYIFIPFFGILAALIV-IKHFRRKRKRNVIVQQSLIAP-----------
-----------LSPCQLISKQDKL-IKLQELIAHGPLS-AVWKGIYGVEYVAVKIIPKTD
FDSWENEVKVYSLFKIPNENILKFYLAKK-HIEN--SCIQHWIVTEYHKNGSLKKFL-KL
NVISLPILKKFFMCFTDGLKYLHSKL-------GHKPAIAHRDIKSKNILVKDDLSLCIS
DFGLSLVF--DGKYFDSKHAKVQVGTKRYMAPEMLEGSISFL--TDSLLFIDMYAFALVL
WEILSRCS--IDEESVNDYIPPFQDMV-GVKPSIDDMLNCVCVKKMRPCF-QSN-WIRHP
LISKICKTISECWDYDAESRLSSSCVYIRFVTLFEQETELINSE----------------
----
>XP_065676910_1_activin_receptor_type_2A_isoform_X1_Hydra_vulgaris
FKSWTVYYILTVYINRFAMDSENKNDCH--PLNCTVTETC--EDPCFTLYSATNELLPHS
SGCWHQDDTHVFLKDECLEHQTTIQNFITYICLCTGHLCNKNVIVTKPPIEMATMLQTLK
QSNKPPYNYKFLYIVVPCFGVITIVGILVYMWR-KNKIINETHLPLI-------------
-----------GLPPPPSPPIISKHIDLHEIISHGQYG-HVWKALYENKLVAVKMMLPSE
```

KDSWETERKIYSNYILHHENILNFHAAEK-RLEN--NYIQYWIITEFHQHGSLTDFL-TF
NIIDLKTLINLSLSIANGLTYLHEN--------MKKPSIAHRDFKSRNVLVKDNFTCCIS
DFGSSFAFDDNADKEKAKFQ---VGTKRYMAPEVLEGAIAFR--TEAFLCIDIYALGLVL
WEILSRCG--ETQAPIGNYQAPYENIV-GLHPSISDMVECVCERKLRPII-LPE-WTEHP
ILKEFCETIEECWEMEADARLSAWCVYERLVEQARNLNNDDISQYKFCDSNYLC------

>XP_052079115_1_TGF_beta_receptor_type_1_like_Mytilus_californianus
LIFMILITIWLNLIGVSD-----GLKCYCD--LCP-NSTCIANAQCLTVVVKGKTGLVHR
FRCLIEGQFLNGEHVSC-NTAKNKKTDNYRLCCDDYDFCNRALMPTLAPPTTTTPSPDED
LDKLEFGTVLVVLISGPVVLVSLLFMIGFFAY--YQYQHRRPSGYMLPPDTSNADVAPFA
QQGTEYSGSGSGLPLLVQRTIARQ-IHLCEIIGKGRYG-EVWRGKWRAENVAVKIFSSRE
ESSWFREAELYQTVMLRHENILGFIAADN-K-DN-GTWTQLWLITDYHENGSLFDYL-NR
TQINIPQMIQLALSAANGLCHLHMEIIGTEVHAG-KPAIAHRDLKSKNILVKNNLTCCIA
DLGLAVRHDIATDSVDIAPN-DRVGTKRYMAPEVLDATLNRT-HFESFKRADVYAFGLVL
WEIARRCS--IG-GIVEEYQLPYYDMV-PSDPSLEEMRKIVCDEKYRPGI-PNR-WQTVE
SLRLMGRLMKECWYHNAAARLTTLRIKKSLAAMTSMEDTKVYIRTA--------------

>XP_052091605_1_activin_receptor_type_2A_like_Mytilus_californianus
ATLRLLFFLLAVAKNCDGEDAVNIITCVIPKSRCSQIEKC--ASSCFIAWQSVNGDIVKH
QGCWTASHTSRCTTSEC--KQDPNIGALNFCCC-NSDLCNTNYTSTTPPEGARPVETEKP
DSVRE----TVLYSLVPIAILVFIIIGIFFMWRFYKDRFGHEQLPTFDP-----------
-----------VQSTPPSPQPLKP-VQLIELRAHGRFG-EVYKGTMATEAVAVKIFPMKE
KVSWMMEQDIYKLPHMKHDNVLRFIAAEK-------RDDKLWLISQFHDLGSLCDYL-KG
HKLTWLDMLKIGESMAKGLAYLHDDIPGAGHSEA-KPSIAHRDFKSKNVLLKSDLTACIA
DFGLALKFDAGKSAGEIHGL---VGTRRYMAPEVLEGAICFN--RDAFLRIDMYACGLVL
WELVSRCS--VAEGQIDNYQLPFEEEV-GTHPTLEDMQEYVVLKKCRPTI-KET-WLRHS
GLEVMINTIEECWDQDAEARVSANCVHERLIALSRLTNVSPVIFSPSEEIPYQISSISA-

>XP_052068713_1_bone_morphogenetic_protein_receptor_type_1B_like_Mytilus_californianus
RILFLSLFISSISIFFIE--QVNGIKCKCD--PCPHNETCEATSKCFTAIRVTYEEHYWT
YGCFHEKDKAGNSVLQC----VPHALPKTIECCDEEDYCNEEIEPDLQDKSTASPEVHPT
ASLSDITQ-IALIVSVSTCLIIFIITITCLYIRYKRNLSQRLLLKDDIGQ-----SESFI
CNGE--SGSGSGIPFLVQRTIARQ-INLIKSIGKGRYG-EVWKGKWREENVAVKIFFTTE
EGSWFRETELYQTVLLRHDNILGFIASDI-K-GT-GSWTQLFLITDYHENGSLYDYL-ST
HILDVDDTLKMVHTISCGLTHLHTEIFGTK---G-KPAMAHRDIKSKNILVKRDGSCCIA
DLGLAVRYISENNEVDVPPI-TRQGTKRYMAPEVLDETLLTH-HFDAYRKADIYAFGLVM
WEICRRCV--ANEGVVEDQQVPYYDCV-QTDPSFDEMRKVVCIEKMRPEI-PNR-WLNDQ
YLKAMIKVMTECWSQNPAARLTSLRVKKTLNKMYQLSQKNEKLELSC-------------

>XP_052092005_1_bone_morphogenetic_protein_receptor_type_2_like_Mytilus_californianus
VITLISLCEECFGRVCISGTTSHNFCCYCGESNSSMTVLCPGDDYCFTVWQVLNKIRIVK
QGCWQKSDDKHCTVGQC--VANRRSTQVKYCCC-YGNLCNTNMSPSQHTTEDPQFPGAKH
LAEEYTQYKTIIISLVSVVSVALIIMGVYLLYRLCLVSKKTSNDSLH-------------
-----------LVEAPPPRGMELEGVEIEKQICRGRNS-EVWQGTMGTMNVAIKMFQSNH
RQLFLNEKYIYSLPFMEHENLLKFYGCEE-RLTM-EGYKQYWVVLSYCPVGNLHCYL-KN
NTVDWSTFCRMALTIVKGLSHLHTQIEKGD---QFKPTIAHRDINTRNILVNPDNTCVIG
DLGFSIATVGHYESAEQASL-QDVGTLRYMAPELLDGAANLRESETSLKQIDIYALGLVI
WEIATRCTCLYNGVPVPDYTLPYQKEA-GLHPTFEEMQILVSRNKVRPKF-SEV-WKDNQ
AVRALKDTMEDCWDHDAEARLTSMCVEERMLDMMTLLNDTQLDNVNENLDTSLVQSDVLN
MNTR
>XP_052080275_1_activin_receptor_type_1_like_isoform_X2_Mytilus_californianus
---------------MTR------FKCACTDHYCPVQNYCESDVGCFVMADYSNDVLIVR
RGCGYDEEKQ---MMNCRRDDNWGSQHVAMECC-IKEMCNKDIIPPVVPVRHDKPVAKEE
QVFNYM---VVLAIIVPVIVLVLSLAIAWYFFRSKNRQESQENLRVREELLPEEYGIRAT
QVGDCTSGSGSGLPFLVQRTIARQ-VTLLECIGKGRYG-EVWRGNYYGESLAVKIFSSRD
EASWARETEIYNTCLLRHDNILGYYASDM-T-SR-NSCTQLWLIMQYHEHGSLYDYL-QR
NVLNHESMLLLATSAAAGLVHLHTEIVGNQ---G-KPAIAHRDIKSKNILVKLDGTCCIG
DLGLAVTHSQENNKIDLGRN-NKVGTKRYMAPELLDETLNVY-YFDSFKSVDVYAFGLVL
WEITRRCY--TN-GMIEDYKPPFWDVV-PTDPSFEDMKKVVVIDQQRPAI-PNR-WSYDP
TLKQMTILIRECWAQNPKSRLTMLRVKKTLNSITQRPKKEKLDDCSDCNHN---------

>XP_032225445_2_TGF_beta_receptor_type_1_Nematostella_vectensis
MGARLILCLVVFLGAAFVMKLNRQITCKCD--YCQENSTCVTKGACLTILQRDSSEVKRG
HACT---YNPPADRLVCEPGYSTERPHQLYHCC-YYNMCNVDTNPSVPPTDPAEGKTSGR
SGRPSTAE-VVAMIVSPVLVICLATVSIIYCYQ-KRNPQRQCILNDGSESPEEVLIPAGK
SLCDISTGSGSGLPLLVQRTVARQ-ITPIELIGSGRYG-DVYRGQWRGEDVAVKIFSSRE
ECSWFREAQIYQTVMLRHENILGFIAADN-K-DN-GAWTQLWLITDYHANGSLYDYL-QR
VTLDMKSMLKLTISIASGLAHLHMEIIGTQ---G-KPAIAHRDLKSRNILVKDNGTCCIA
DLGLAVCHNSEQDTLDIPYG-NRVGTRRYMAPEFLEDSNQVR-NFCAYKHGDIYAFGLVL
WEITRRCI--CS-DKCEDYQLPYFDKI-CVDPSIEEVWKVVCLEKYRPSH-PNY-WLQDQ
VMMRVAKLMQECWHHSPEARLPALRIKKTLQSLYTEECSDHCVKA---------------

>XP_032222986_1_activin_receptor_type_2A_Nematostella_vectensis
PLFWSFLALFCGLLRADYLQTCESFGQLCHETGECNTIVCFRESHCYTLWENTTENIIK-
KGCFIRNHLECRGDHQC---RTKGGRTVFFCCC-SDHFCNANFSVHSPPITGITTLHATA

RPSRDVQKHTLLYSIVPIFGVILILAFVWFMYNHQKGISSRSCNAEESEPI---------
-----------NITTTPLQDVNRRPIQLLEVVSQGQFGVTVWKAKYSINTVAVKIFPHQE
FTAWVNERDFYKLCGLDHENVLTFIGDEVHHESQYNIYTEYWLITDFHENGSLSDFL-KQ
RSLDLAELLQLASSVCDGLAYLHSDVPAINNSSH-KPCIAHRDVKSKNILVKSDLTCCIS
DFGLALKFDQSDKLGETHGQ---VGTKRYMAPEVLEGAISFT--RESFLRIDMYACGLVL
WELLSRFS--VHDDPVEDYRLPFEQEV-GLHPSLEDMQQVVVQKKMRPQF-KDS-WRCHA
ALRVIIQTIEECWDQDGEARLTSHCVAQRLKQVAIVPTCTEQVSIVTPVINNRTTPPSRE
SSI-
>XP_032238543_1_bone_morphogenetic_protein_receptor_type_2_isoform_X1_Nematostella_vectensis
TMSWIWRFLVLLSLAFTG--FVKSKRLYCESYGNKDTEVCDPYFYCMVVWSTRFLDVVS-
KGCFGQKHPC---NHNCFDSMAKSKNISGFCCC-DTDYCNKNFTVEYKEKVVPTRTTGGK
VHLTSNIN-IIVGIVLATLLLITILVLFFWHYNNKKHKYSRPDKSLEKEPQ---------
-----------GPSLDLSQ------LTLGQLIGQGRYG-TVWKGTMFNEVMAVKVFGAEH
RLFWQAEKDLFSA--LDHPSILRFFAACE-R-ET-NSSPEYLLLSEYHPRGSLQNYL-RQ
HSVCWREMCIMGSSMSAGLAFLHQD--------ANKPSFAHRDINSKNILVKVDGSCVLA
DFGFAMNVSDATKPADDGTLITEVGTLRYMAPEVLDGAVNLHDIQGALKQIDVYAMSLVI
WEVSMRCEDIFGREQVPPYKAPFEAEL-GPMITSEHMHTYVVQKKLRPGF-PDA-WKRHS
GLRFLKETIEDCWDQDGDARLLALCIKDRMSDLLTHYPNGMNDSLQSSAGTVQEFQENLK
PEAQ
>XP_001633896_2_bone_morphogenetic_protein_receptor_type_1B_Nematostella_vectensis
TIFWAFCIGICLLIEVSQ------ARCKCSEHSCPGNDTCTTTGKCYKKIAEEEGYELIT
YGCLPEEQTD----MQCNTPASAHRNKISVLCCNNRDLCNFELDPTFPPPTSPTIDVRAR
VDDESSGSKFILFVTAGVCTVVLVIVLVILYIRFDVRRRLPLSYDLERRINFISSGETLK
DYFDQSSASGSGLPLLVQRTIAKQ-VTLVQSVGKGRYG-EVWKARWHGEDVAVKIFLSHC
EKSWMRETEIYQTVLLRHESILGFIASDI-I-GS-GQVTQMYLIMDYHPLGSLYDFL-RG
HQLNKKITGKLAFSAAAGIAHLHAEILGTQ---G-KPMIAHRDIKSKNILVKENLTCCIA
DFGLAVKYIPETKGIDLNKDTNRVGTKRYMSPEVLSQTMDPE-SFSAYKMADMYSFALVL
WEISRRCISDET-GLCEEYEVPYFDAV-SNDPTFEEMKRVVVLERRRPNI-PNR-WFRDE
MLRTMGKLMAECWSQQPAARLTALRVKKSLSKLVSAINSHEVIPAAAMSPD---------

>XP_048588435_1_bone_morphogenetic_protein_receptor_type_2_Nematostella_vectensis
LLLVCIIMLRCFRVTDGL-------ICY--NHENPDTEVCDGAFFCYVLWLDSPDPALLY
RGCVYQDFIQPCNKPVCPEYNSTLSSDIHVCCC-NEDFCNKNYSPRTKTHWSTPNNHRKE
TAFTQPLTIIIYALIVVLSLLLLILIVAVYC---IVKPYHMKEPISEQADIPRDIGQPAN
S----------IKEAEFEQMFDAD-LSKFHLIASGTYG-QVLSGFWKGKEVAVKLYTNDN
EKYWRNEVMVYKAKG-NHPGICRLITSAV-------IGPRLAIVTEYHPCGSLANYL-TR
TSVSWAEMCTLASSAARGLAHLHDMVS--------IEAYAHRDLCSSNILVKRDGTCALT
DFQFTKKLSRDLNKVEQIEI---AGTMRYWSPELLDQAISFEFFCESLCQADVYSLSLIV
WEIVMRCRDIYPDGTVPHYRQAYEDEL-GKNTTRAELDKHVRVLGLRPEF-PRI-FRQSQ
ALYILRQTLVECWDSDPDARIHPALMAFRLEGILSEKPELKNRGEQCKCVSIMIHDDEVG
QAL-
>AFP87425_1_activin_receptor_type_I_Nematostella_vectensis
RATQRILFHRLIQASVNINRTSVNVRCICS--DCRSEQTCSTETGCFSSLFLLENQSSVT
KGCL---KDADHYNMMC---RGPRGKPNDTRCC-THDLCNKHINPTHAPSTIPPIKQEKL
DVQF-----LIISICAPVAALIVCLIIVYMIFR-HLMKHHAHHQSVPRHDLGPVSHVQCP
CNEEISSGSGSGLPLLVQRTVARE-TQLIESIGKGRYG-EVWKGVYQGESVAIKIFSTTD
EASWKRETEIYNTVMLRHDNILGFIAADV-HSRK--STTYMWLIMHYHEQGSLYDFL-NR
STFDAETLCRLALSAASGLAHLHTEIFGTNRTGS-KPAIAHRDIKSKNILVKSNYTCAVG
DLGLAVMYSQDKDSVDMGEN-PKVGTRRYMAPEILEETINAK-CINSFKRADVYAFGLVL
WEIARRYV--SG-GIVEEYQPPFYDMV-QSDPSIDEMKKVVVTENKRPSL-PNR-WTGDP
TLQVMSKLIRECWNPNPAARLTSLRIKKSLLKLMDSPKQKSDSFATDVIAPTVSSHISEA
SSPP
>XP_001641146_2_activin_receptor_type_1_Nematostella_vectensis
IPAAIGCSLSGIGSRLRGNRTSVNVRCICS--DCRSEQTCSTETGCFSSLFLLENQSSVT
KGCLKDADHY---NMMC---RGPRGKPNDTRCC-THDLCNKHINPTHAPSTIPPIKQEKL
DVQF-----LIISICAPVAALIVCLIIVYMIFR-HLMKHHAHHQSVPRHELGPVSHVQCP
CNEEISSGSGSGLPLLVQRTVARE-TQLIESIGKGRYG-EVWKGVYQGESVAIKIFSTTD
EASWKRETEIYNTVMLRHDNILGFIAADV-H-SR-KSTTYMWLIMHYHEQGSLYDFL-NR
STFDAETLCRLALSAASGLAHLHTEIFGTNRTGS-KPAIAHRDIKSKNILVKSNYTCAVG
DLGLAVMYSQDKDSVDMGEN-PKVGTRRYMAPEILEETINAK-CINSFKRADVYAFGLVL
WEIARRYV--SG-GIVEEYQPPFYDMV-QSDPSIDEMKKVVVTENKRPSL-PNR-WTGDP
TLQVMSKLIRECWNPNPAARLTSLRIKKSLLKLMDSLKPRNNCLNRTPLITEVSDVNIHL
ESME
>AFP87430_1_bone_morphogenetic_protein_receptor_type_II_like_protein_Nematostella_vectensis
----

---MVSIEAYAH--

---RDLCSSNILVKRDGTCAIT
DFQFTKKLSRDLNKVEQIET---AGTMRYWSPELLDQAISFEFFCESLCQADVYSLSLIV
WEIVMRCRDIYPDGTVPHYRQAYEDEL-GKNTTRAELDKHVRVLGLRPEF-PRI-FRQRD
TLHLTSPSVITTLHLTDTLHLTSPCVITGIHYTLQQLHLTSPCVITGYTTPYITLCNHRI
HYTL
>UVJ89029_1_RAF_Paracentrotus_lividus

GNEQPPPAIFLEEYQDLTAFLPNQQRTSVLKHSLSKPEMCRTNPRMALSWETDMMELKA-
QNFPNFVRKTFFTIAFC---LLFHGFRCQTCSYKFHQRCANKVPPRERSTSAPNICQNAV
NVPGQQFDSPAKSSLNPSLASSLPNTTNMIVHSPKRESGSHSSQPSPTHQPRFKNALSGS
EHKD-------RRDSNDDWEIPADEIRLGVRIGAGSFG-TVFSGQWHG-SVAVKRLNVKD
PQAFKNEVAVLRKTR--HANILLFMGCTS-K-------PQLAIVTQWCEGSSLYKHL---
HVLDMHQLIDISRQTAQGMDYLHAK------------NIIHRDLKSNNIFLHDDLTVKIG
DFGLATVKSRWSGSQQFEQP---SGSILWMAPEV----IRMRDKNPYSLESDVYAFGVVL
FELVTQ-------------SLPYQNIK-----HKDQIIWMVGRGYLQPDL-SKV-RND--
TPKALKRLITDCYALNRDERPLFPVILASLEMLARQLPKIHRSASEPSSLNRARLHSDDF
MYCP
>ABG00201_1_FGFR1_Paracentrotus_lividus
AILYVICCWYIVTYCVKSSNTKVRLDCGARTWLKDGIIRKTGKYTCLVSNQHGTIERLPV
KPIMVTAVVGSNTSFVCQRLEDIDNHISMLTCCVAGNFYGMSYEDIVEPTTKAPVKTNNP
PAVFIPTNNLIIFCVVGFVIVVILVTCIAILCKQVRHRRPVDKPHVSKPLVYRQMSIDST
KSNACQFGRNRDIPLDPEWEFPRDRLTLGKTIGEGAFGKVVIGEAVGIVTVAVKMLKARE
FSDLISELAMMKMIG-KHPNIINLLGCCTQE-GP-P-----YVIVEFAHHGNLRDFLRSR
QTLTNKDLMSMAYQVSRGMAFLASK------------KCIHRDLAARNVLVTEDFEMKIC
DFGLA----RDIHYIDFYRK-TTRLPVKWMAPEALFDRMFTT-------QSDVWSFGILL
WEIMTLGGTPYPSVPVEDYLRSGKRLEKPQNTSLEIYHIMCECWRTSPGQ-RPN-FSE--
LVEDLDRIISVSSNQDGDAPVKTFQESERMAFMGFKAPLSPQVYYKVPQTRDCCPYAN--

>ACU12850_1_bone_morphogenetic_receptor_type_I_Paracentrotus_lividus
KAWWNLSFFSAFVGLLSSSVLATEMYCYC-DEHCPENNTCYPGGWCFAQIQAGEDTPIRS
YGCLPEEEGG---LMQCKGQLSSHRISRTVYCCNDEPNCNLKLNPTLPPTTTSSTTELPG
VEDTYNTTVIALLISVTFCAAAFIIALTFFYLRYKREVRRRYDFEVAQQDDSFIGAGETL
SELLSASGSGSGLPLLVQRTIAKQ-VQLIRKIGKGRFG-EVWKAKWRGENVAVKIYFTAE
EASWFRETEIYQTVLMRHTNILSFIAADI-R-GS-GAYTQLFLITEYHECGSLYDFL-GV
NILDTQSMLRLAYSASNGLAHLHTEICGMQ---G-KPAIAHRDIKSSNIMVRKNGTCMIA
DMSLAARFLSEGNEIDLGQN-TRLGTNRYLPPEILDNVMMRN-TFDAFKMADMYSFGMVL
WEIARRCV--TR-GIVEECQLPFYDAV-PLDPSIEDMRRVIVVERRRPDI-PNR-WSGDD
ILRTVSKVMSECWNHNPAARVTSLRVKKTLGKLQETEFKV--------------------

>ARV86154_1_activin_receptor_type_II_Paracentrotus_lividus
RLCKLFLFLSVVVVLLEGVNLPLGSHCLYSGTKCGNTEECNKEASCYVLWENETSIQVMM
RGCW-QNQGDCLDMDFC-MSSSPADQNLFFCCC-VGNLCNLNFSPEPTPTVTQTTVTGSS
IDLEKKRKNTILYSIVPIVAITLVIIMLYWMCR-RHRYHSLIAIPTQEPSPSPMS-----
-----------SLPP----------IQLIEIKARGRFG-AVWKASCLTDIVAVKVFPIQD
RNSWANEREIYNLPHMRHENILNFIATEK-H-GE-GLEVEYWLISEYHHLGSLCDYL-KA
NVISWKELCGIALSMAKGLAFLHEDIPATTAIPFIKHSVAHRDFKSKNVLLKNNTTACIA
DFGLACVFEAGKNPGDTHGQ---VGTRRYMAPEVLEGAIQFK--RDAFLRIDMYAFGLVL
WELVTRCT--IQDGPVPEYRMPFEEEL-GQGTSLEDMQEWVVVKRKRPVI-SDH-WLKHR
GLELLCETVEECWDQDAEARLSAGCVEERIAQFSHSHNSTSFENTGNTESTDIALPVYRS
SPSP
>AIF71193_1_Alk1_Alk2_Paracentrotus_lividus
RLEFIFILLALKVDFFVSEADNNTLQCYCDDYSCEGTNQCQALRKCFSSITLVDGIIMKR
MGCVNTEEQF---NFFC---KMHPYPDISVLCC-NHSFCNTDLQPTFVPTTTPRITEEIG
HMVPYNVTYTVLGVVCPLIACLIALVIAGYCFR-RRHRQRMDELSHQEAQYRQAHDIRAV
PAGDNTSGSGSGLPYLVQRTVARQ-INLIQQVGKGRYG-EVWMGTWQGDTVAVKIFSSID
EKSWFRETEIYNTVMLRHENILAFFASDM-T-SR-QSCTQLWLIMEYHERGSLYDYL-NR
HVLDARQMCRLALTACSGLVHLHTEITCNNS----KPAIAHRDIKTKNILVKRNLTCCIA
DLGLAVMHTQRDDFIDMGTN-TRVGTKRYTAPELLEETMDVR-CFESFKRVDVYAFGLVL
WEISRRCE--VG-GMVADYKPPFFEYV-PSDPSFDDMRKVACIEKLRPNI-PNR-WTNNT
VLQSMAKLMKECWCPTASARHTSLRVKKTLMKISETCPKLPDPYHWDKIIA---------

>XP_009300361_1_XP_009300361_1_activin_receptor_type_2A_like_isoform_X1_Danio_rerio
---MVK
QGCW-LDDVNCYDSSEC--VERKENIDVFFCCC-EGNLCNQKFHETVEPTLNPVPPKPDL
FP-------TLLYSLLPIMAVAVILFISFWMYRHLKLTYPPLLVPSQDP-----------
-----------GLTPPSPLLGQKP-LQLLELKARGRFG-CVWKAQLLSEAVAVKIFPVQN
KQSWQNEYEIYNASGMKHENLLHFIGAEK-R-GN-GVDIELWLITTYHEKGSLTDFL-KA
NVLSWNELCLIAQTFVRGLAYLHEDIPNLKD--GHKPAIAHRDIKSKNVLLKSDLTACIA
DFGLALKFEAGKSTGDTHGQ---VGTRRYMAPEVLEGAISFQ--RDAFLRIDMYAAGLVL
WELATRCT--AADGPVDEFCLPFEEEA-GLHPSLEDMQDVVVHKKLRPIF-REH-WLKHT
GLSLLCETMEECWDHEAEARLSAGCVEERIISMQRSTSIISPDDILSVVTMVTNLDFPPK
ESSL
>XP_021334336_1_XP_021334336_1_activin_receptor_type_2A_isoform_X1_Danio_rerio
KLAFGVFLISCSSGAILG---RSETECFYNRGNRSGIEPCKR--HCFATWRNVSGVEIVK
QGCW-LDDVNCYDSTEC--VEKKEDPDVFFCCC-EGNMCNEKFFPNTAPVQSKAPFSDTK
-SDTKVFKATLLYSIVPIMGIAAIVLLSFWMYRHHKLAYPPVLVPTQHAFHIMIED----
-----------PGPMPPSPTLVQKPLQLLEIKARGRFG-CVWKAQLLNDYVAVKIFPIQD
KLSWQNEYDIYNIPGMRHENILQFIGAEK-RGSN--LDIELWLITAYHEKSSLTDYL-KA
NVVTWNELCHIAQTMARGLAYLHSDFPGHRD--GHKPAIAHRDFKSKNVLLKTNLTACIA
DFGLALKFEAGKSAGDTHGQ---VGTRRYMAPEVLEGAINFQ--RDAFLRIDMYAVGLVL
WELAARCT--ASDGPVDEYMLPFEEEV-GQHPTLEDMQEVVVHKKLRPTL-REC-WQKHP
GLAMLCETIEECWDHEAEARLSAGCVEERVVQMQRQTSVSAPEEIVTVVTMVTNVDYPPK

ESSL
>NP_001034906_1_NP_001034906_1_bone_morphogenetic_protein_receptor_type_2a_precursor_Danio_rerio
ITILDVGLIAMVVLLGPVAQSEQR-ECYAERIGGRGTIRCYQGHRCFGLWGKKNNVVLVK
QGCWTNVGDHQECYDRCNPPSVAQNGTYRFCCC-NKDMCNLNFTEDFPPPSPTTAQPLQS
RRLHREE--VIVVALATVSMVAVLVILLFFGYRMGRGSHSLHTLNI--------------
-----------LETALSPPSLDLDNLTLQELIGRGRYG-TVYRASLDDRSVAVKVFISAN
RQQFTNERMIYRLL-LDHENIARFLESEE-RVGT-EGRTEFLLLLEFYPHGSLCTYL-SG
RTVDWLSCCRLALSVTRGLAYLHTEIQRGD---VYKPAVSHRDLNSRNVLVKTDGSCVIS
DFGLSMILTPGHGEEDNSAI-SEVGTVRYMAPEVLEGAVNLRDCESALKQVDVYALGLVY
WETFMRCADLFPGETVPAFQLAFQAEV-GNHPTIEDMQALVSREKERPKF-PEA-WKENS
TVHSLKETMEDCWDQDAEARLTAQCAEERLAELLLITNLCAQLTREDLEIPKLDPSEVQR
NMRE
>AAI63200_1_Bone_morphogenetic_protein_receptor_type_II_b_Danio_rerio
NRTSVLSLGLCSLLLLSAQSEEREREСFNDQSVAGDTILCSKSSRCYGLWEKTHDIRLVK
QGCWYIGDQQDCHDDRCTTPSQIQNGTYRFCCC-STNMCNVNFTENWVPSPTSTANRDQP
PIHRDE---AIFIALASVSIVAVLIVALFFGYRVGECKQGLHNMDM--------------
-----------IEAAPSEPSLDLDSLKLLELIGRGRYG-SVYKGSLDERPVAVKVFTYAN
RQNFVNERSIYRVPLLEHENIARFIVGDE-RMTT-DGRMEYLLVMEYYPHGSLCRYL-SL
HTLDWVSCCRLAHSLTRGLAYLHTELLRGDIY---KPAISHRDLNSRNVLVKNDGTCVIS
DFGLSMKLTVRPGEEENAAI-SEVGTIRYMAPEVLEGAVNLRDCESALKQVDMYAIGLIY
WETFMRCTDLFPGETVPEYQMAFQAEA-GNHPSFEDMQVLVSREKQRPKF-PEA-WKENS
AVRSLKETMEDCWDQDAEARLTAQCAEERMAELLLIVDKNLKESSDENLMEHSQKQFSAP
DPLS
>NP_878275_2_TGF_beta_receptor_type_2_precursor_Danio_rerio
YQLSVLCWWTFLISTVVDPYRIPQL-CFCDGKDTC-ESNCCEDEICVAIWRQSDDNNTIE
TLCHYGIMVENNNNTKCMKKRISNSGPFHICSC-NEVECNDMLLFKLP-----TNNQVVL
---------VILVSLLPLLVLAVLVIASFYWYR-LYRRRKLDEWETKKPSKHKGPKGALD
CSDADRSDSSSTHANNINHNMDPLPIELDLQVGKGRFA-EVYKAKLRQSTVAVKIFPYEE
YASWKNEKDIFQDIDLKHENILHFLTAEE-RK----VEKQYWLITAFHPRGNLQEYL-MR
HLLSWEDLRLLGSSLASGVAHLHSDPCGRP-----KVPIVHRDLKSSNILVKNDLTCCLC
DFGLGLRLDNSLSVDELANS-GQVGTARYMAPEVLESRINLE-NIESFKQTDVYSMALVL
WEITSRCN--AI-GEVKDYEPPFGSKV-REHPCVESMKDNVLRDRGRPEI-PSS-WMKHQ
GVAAVCATINECWDHDPEARLTAQCVAERFNEMDDDLDKLSTCSSSEEKIPEDCAVSVSD
DK--
>XP_009292206_2_TGF_beta_receptor_type_2_Danio_rerio
SGSVLLVFLILISECVLQSGFRLKQNCFCDASGVCENSICTPDEVCASAWRSNGGSIE--
TVCHHGHNLTDSQSRVC---MRRVEEDLYICSC-NDEECNDRLFNHDLKQYPGVQFDPPS
-------ESLAPVLISVFVLIPIVLLSVFYCYHIKLKLPNKKSLDSERCNIFMNPEEHSD
SSSI-------NANSLNHNTELLP-IQLDRVVGKGRFA-DVYKAKLKQSTVAVKIFPYEE
FSSWKSEWQIFSDVELRHENVLQFLTAEERT-----AERQYWLITAYHERGNLQELL-AL
QVIGWEEMCRLGVSLARGVAHLHSDRTPCGRA---KVSIAHRDLKSSNVLVKSDWSCCVC
DFGLSLRLDSCLSPEELANS-GQVGTARYMAPEVLESRMDLE-NTESFKQADVYSMALVL
WEIMSRCS--AT-GDVRDYEPPFGKL--KGHPCVESMKDDVIRDRLRPEI-PTS-WSKHA
GVHHLSSTIEECWDHDPEARLTAQCVVERFDDIIN-SDVILPISPDTKSAGDSGIQEM--

>XP_688938_2_TGF_beta_receptor_type_2_Danio_rerio
TRTSALLCCFCLLQVQSLSPIKTN-LCWCDDNVC--MSNCCFEEVCVAIWKKDNVSVSVR
TLCYENIMLANYSSKECLMSPHPSEDGLLFICCVGDQECNDRLIDKGPGFSKLKSKDVIP
---------VVVISLVPPLLVAVIATMAFYLYRTWGPRRTHYQSLDPAEGQANGIDFSAK
RPSL-------CANNLNHNTEQLP-IQLEALVGKGRFA-EVWRARLSHNTVAVKIFPAVE
YSSWCNERAIFSDANLKHENVVQFLTAEE-RGGT-SSQRQYWLIMAYYNMGNLQDFL-VG
NILTWAELCSLAGSVARGLAHLHSDPCGIQ-----KVPIAHRDLKSSNIVLKNQSECVLC
DFGLALRLDLSLTVDDFANS-GQVGTARYMAPEVLESRVNLE-DLESFKQIDIYSMALVM
WEMVSRCD--VI-GEVKSYEPPFGSKV-CEQPCVDSMRDLVLRDRGRPDI-PDS-WTTHS
GMQLLCATITECWDHDPEARLTAHCVVERFNTLAQ-EELENSICTHTPSTPPTEDQTPTL
PSHP
>NP_001108531_1_TGF_beta_receptor_type_1b_precursor_Danio_rerio
MMMEAVPCRLVFLAVFFGVQQTEGLQCYCH--RCP-NHTCTTDGLCYVSITRAGSLTTQQ
SWCIKGELVPPDRPFVC--APSTKDDSGIYPLCCDTDWCNKDPDKAFPPTQKPPALGPVA
---------FAALIAGPVCVLCFLMVTVFYICHSRSVIHHRVPSEEDPSL-----DHPFI
TVGTTTSGSGSGLPLLVQRTIART-IILQESIGKGRFG-EVWRGRWRGEEVAVKIFSSRE
ERSWFREAEIYQTVMLRHENILGFIAADN-K-DN-GTWTQLWLVSDYHEHGSLFDYL-NR
YTVTVEGMIKLSLSTASGLAHLHMEIVGTQ---G-KPAIAHRDLKSKNILVKKNGTCCIA
DLGLAVRHDSATDTIDIAPN-HRVGTKRYMAPEVLDDSINMK-HFESFKRADIYAMGLVF
WEIASRCS--IG-GIHEDYQLPYHDLV-QSDPSVEEMRRVVCEQKLRPNI-PNR-WQSCE
ALRVMAKIMRECWYANGAARLTALRIKKTLSQLSQQEGIKM------------------

>NP_001032772_2_TGF_beta_receptor_type_1a_precursor_Danio_rerio
LTSRSLLLLALLVLSVTHE---AQGLLCYCE--RCV-NRSCNTTGLCFAVIAKSSGTVTQE
RQCIQDDLYPRDRPFQC----APSNNGKHPYCC-NTHMCNKNPKPELP-EAEPQSMSPIA
---------LAAMIAVPICVLSFVLVLLFYMCHNRSIIHHRVPSEEDPTMDHPFLADGTT
LKDLTTSGSGSGLPLLVQRTIART-IILQESIGKGRFG-EVWRGRWRGEEVAVKIFSSRE
ERSWFREAEIYQTVMLRHENILGFIAADN-K-DN-GTWTQLWLVSDYHEHGSLFDYL-NR
YTVTVEGMIKLSLSTASGLAHLHMEIVGTQ---G-KPAIAHRDLKSKNILVKKNGTCCIA
DLGLAVRHDSATDTIDIAPN-HRVGTKRYMAPEVLDDSINMR-HFESFKRADIYALGLVF

WEIARRCS--IG-GIHEDYQLPYYDLV-PSDPSVEDMKRVVCDQKLRPNI-PNR-WQSCE
ALRVMAKIMRECWYANGGARLTALRVKKSLSQLSQQEGIKI-------------------

>XP_687633_5_activin_receptor_type_1B_Danio_rerio
QILRILIVLSGLNGVCDA------LLCNCTAPHCERGFKCETNGACVASTSVIEGQEQHV
RLCIKEKLVPPGQPFYC----LSAEGLMNTHCC-YTDYCNSIDLIVTNGPGAGQDWGPVE
---------LTAVVAGPVFVLCVLVLLGLFLFQHQRAYGHRQRLEVEDPSTEHMFLAKDK
TLQDSTSGSGSGLPLFVQRTVART-IVLQEIIGKGRFG-EVWRGKWRGGDVAVKIFSSRE
ERSWFREAEIYQTIMLRHENILGFIAADN-K-DN-GTWTQLWLVSDYHENGSLFDYL-NR
YSVTIEGMIKLALSAASGLAHLHMEILGTQ---G-KPGIAHRDLKSKNILVKKNCTCAIA
DLGLAVRHESITDTIDIAPN-QRVGTKRYMAPEVLEESINMR-HFDSFKCADIYALGLVY
WEIARRCN--AG-GIHEEYQLPYYDLV-PSDPSIEEMRKVVCDQRLRPNI-PNW-WQSYE
ALRVMGKIMRECWYANGAARLTALRIKKTLSQLSVDEDLKI-------------------

>NP_571065_1_activin_A_receptor_type_1Ba_precursor_Danio_rerio
RTAVALLALCGLLAVGDA------LKCNCT--ACESGYVCETDGACMASTSYINGEEQQV
RICIRVSLVPPGQPIYC----LSAKGLLNTHCC-YTDFCNSINLPNGIPDGKGGSWGPVE
---------LVAVIAGPVFLFCLLLIVGVLLFQHQRNYNHRQRLDVEDP------SCDHL
YLAKSTSGSGSGLPLFVQRTVART-IVLQEIIGKGRFG-EVWRGRWRGGDVAVKIFSSRE
ERSWFREAEIYQTIMLRHENILGFIAADN-K-DN-GTWTQLWLVSDYHEHGSLFDYL-NH
YSVTIEGMIKLSLSAASGLAHLHMEILGTQ---G-KPGIAHRDLKSKNILVKKNGIFAIA
DLGLAVRHESITDTIDIAPN-QRVGTKRYMAPEVLDETINMK-HFDSFKCADIYALGLVY
WEIARRCN--AG-GIHEDYKLPYYDLV-PSDPSIEEMRKVVCDQRLRPKW-PNW-WQSYE
ALRVMGKIMRECWYANGAARLTALRIKKTLSQLSVQEDIKI-------------------

>XP_021334502_1_activin_receptor_type_1C_Danio_rerio
CSDAALFIFTFVQLTAAL-------KCVCH--LCV-NHTCETEGACWNSVMLINGKEETV
KSCVPSELKG---QVFC---YSSRNVSKRNCC--FTDFCNNETLPEQP--PEDSGWSQLE
---------VAAVILVPSCLVCVGVMLGVCAIQNRCTHIKSLKQDPEEPLDDPTLVSPDK
CLKESTSGSGSGLPLLVQRTIART-IVLQETIGKGRFG-EVWRGKWRGEDVAVKIFSSRD
ERSWFREAEIYQTIMLRHDNILGFIAADN-K-DN-GSWTQLWLVSEYHEHGSLFDYL-NR
FTVSVEGMIVLALSIASGIAHLHMEIIGTQ---G-KPAIAHRDIKSKNILVKKNGAAVIA
DLGLAVKHDSNTNTIDIPIN-HRVGTKRYMAPEILDDSINMS-SFESFKRADIYSLSLVF
WELARRCS--IQ-GIHEDFQLPYYDQV-QSDPSLDDMRRVVCEQKLRPNI-PNQ-WQSCE
ALRVMGKIMRECWHANPAARLTALRVKKTISQVTVVKDVKE-------------------

>NP_001139468_1_bone_morphogenetic_protein_receptor_type_IBb_isoform_1_precursor_Danio_rerio
EKFWTLILLGVLWIYSSASVIPERMLCHC-YHHCPENNTCRTDGYCFTMAEEEEGSPVLT
SGCL----SLVGSEFQC-RDTGNARLRRILECCTDQDYCNQDLHPTLPPLKTPDVDSSVH
Y--------TALLISAMLSFIILGVIMFYCYFRYRQGTQTRYSMGLEQDESFIPAGESLR
DLIEQSSGSGSGLPLLVQRTIAKQ-IQMVKQIGKGRYG-EVWMGRWRGERVAVKVFFTTE
EASWFRETEIYQTVLMRHENILGFIAADI-K-GT-GSWTQLYLITDYHESGSLYDYL-KS
TTLDTRALLRLSYSAVSGLCHLHTEIFGTQ---G-KPAIAHRDLKSKNILVKKNSTCCIA
DLGLAVKFISDTNEVDIPPN-TRVGTKRYMPPEVLDESLNRC-HFQSYIMADMYSFGLIL
WEISRRCV--SG-GIMEEYQLPYHDLV-PSDPSYEDMREVVCIKRQRPSF-ANR-WSSDE
CLRQMGKLMSECWAHNPASRLTALRVKKTLAKMSESQDIKL-------------------

>NP_571532_1_bone_morphogenetic_protein_receptor_type_1B_precursor_Danio_rerio
LCVLLIGLLGLVEENLANPALPQRFWCYC-YHHCPENNTCRTDGYCFTMVEEEGGAAVVT
SGCL----GLVGSEFQC-RDTGNSKQRRALECCTDQDYCNRDLHPTLPPLRTPSYVVGDI
HH-------IALLISVSVCSFILTFIIIFCYFRYRHELAPRYSLGLHPDESFIPAGESLR
DLIEQSSGSGSGLPLLVQRTIAKQ-IQMVTQIGKGRYG-EVWMGRWRGEKVAVKVFFTTE
EASWFRETEIYQTVLMRHDNILGFIAADI-K-GT-GSWTQLYLITDYHENGSLYDYL-KC
TTLDSRAMLKLAYSSVSGLCHLHTEIFGTQ---G-KPAIAHRDLKSKNILVKRNGACCIA
DLGLAVKFISDTNEVDIPLN-TRVGTKRFMAPEVLDETLNRN-HFQSYIMADMYSFGLIL
WEISRRCV--TG-GIVEEYQLPYHDHV-PNDPSYEDMREVVCIKRIRPSF-PNR-WSSDE
CLRQMGKLMTECWAHNPASRLTALRVKKTLAKMSESQDIKV-------------------

>AAH81626_1_Bone_morphogenetic_protein_receptor_type_IAb_Danio_rerio
IIVFGVLVAAVLLSPLAEEDAARFLSCYC-SGHCPENNTCETNGHCFAIIEEDEHEAMLT
SGC-MKYEGS---HFQC-KDSPNAQTRRTIECC-STDFCNRDLQPTLP----PPIPGKPP
FWNAHL---LAFLISVTVCCFTLVAITIVCYYRFLQTGRRHYQRDLGPIEAFIPAGESLK
DLISSSTGSGSGLPLLVQRTIAKH-IQMVRQIGKGRYG-EVWLGRWRGENVAVKVFFTRE
EASWFRETEIYQTVLMRHENILGFIAADI-K-GT-GTFTQLFLITDYHENGSLCDYL-KY
TTLDTQALLRLAYSAACGLCHLHTEIYGTQ---G-KPAIAHRDLKSKNILIKKNGTCCIA
DLGLAVKYNSDTNEVDVPLS-TRMGTRRYMAPEVLDETLNKN-QFQAYIMADIYSYGLIV
WEMARRCV--TG-GIVEEYQLPYWDMV-PSEPSYEDMREVVCVKSMRPVV-SNR-WNSDE
CLRVMLKLMSECWAHNPASRLTALRVKKSLAKMVESQDIKI-------------------

>XP_005156936_1_bone_morphogenetic_protein_receptor_type_1A_isoform_X2_Danio_rerio
VVLTGVCLLLTLCSGGQNEDAARFLSCHC-SGHCPDNNTCETNGQCFAIIEEDENDVILS
SGC-MKYEGS---HFQC-KDSQFAQTRRTIECC-QFDFCNQDLKPELPPRDSEPPDPHW-
---------LAFLISVTVCFCALICVTVICYYRYWQTERQRYHRDLEQ-------DEAFI
PAGEQTSGSGSGLPLLVQRTIAKQ-IQTVRMIGKGRYG-EVWLGRWRGEKVAVKVFFTRE
EASWFRETEIYQTVLMRHENILGFIAADI-N-GT-GASTQLYLITDYHENGSLYDYL-KF

TTLDTQALLRLAFSAACGLCHLHTEIYGTQ---G-KPAIAHRDLKSKNILIKKNGTCCIA
DLGLAVKFNSDTNEVDLPLS-TRMGTRRYMAPEVLDETLNKN-HFQAYIMADIYSYGLVI
WEMARRCV--TG-GIVEEYQLPYYEMV-PSDPSYEDMLEVVCVKGLRPTV-SNR-WNSDE
CLRAMLKLMSECWAHNPASRLTILRVKKTLAKMVESQDIKI-------------------

>NP_571420_1_activin_receptor_type_1_precursor_Danio_rerio
IILFLLQFLQTSAKDVSI-------DCMCVGSDCN-EQQCTGD-QCYTSVIISNDVTTFK
RGCL---IGPASKRMTC---SATASASHVVECC-SQHMCNANVSLTSPEEKKTVHYRVEM
---------LVLFVLGPFVVLGLLSFLALLVC--RRLHHGRLERLHEFDTEQGAIDGLIA
SNVGCTSGSGSGLPFLVQRTVARQ-ISLVECVGKGRYG-EVWRGQWQGENVAVKIFSSRD
EKSWFRETEIYNTVLLRHENILGFMASDMTSR-N--SSTQLWLITHYHENGSLYDYL-QR
VAVEMADGLHMAASIASGLVHLHTEIFGTE---G-KPAIAHRDLKSKNILVKKDLQCCIA
DLGLAVTHTQSDNQLDVGNN-PKVGTKRYMAPEVLDETIQTD-CFDAYKRVDIWAFGLVL
WEIARRTI--SN-GIVEEYKPPFYDLV-PNDPSFDDMRKVVCVEQQRPFI-PNR-WFSDP
TLSALVKLMKECWYQNPSARLTALRIKKTLDKIHSSLEKGKTDC----------------

>AAI00044_1_Acvrl1_protein_Danio_rerio
SVMLAVLFVLVYSGSYATKNDESSLQCSCENTNLCENNSC--GRICFYTS-----HERVV
RGCF--------TAEQC---YVPAIPGVYTKCC-YTHHCNANLTPEKP------VEKPIR
---------VILLVGVPLLVLLVMAMALVLWLRTRRQYCHPVEHDTSMLKVPSGGDPTYG
DIFDCTSGSGTGLPYLVQRTMARQ-IFLVECVGKGRYG-EVWRGTWMGESVAVKIFSSRD
EQSWFRETEIYNTVQLRHENILGFIASDM-TSKN--SSTQLWLVTHFHELGSLYDFL-QY
STLDPEGCLRMCLSIASGLVHLHTEILSTQ---G-KPAIAHRDLKSRNILVKRNGQCCIA
DLGLAVIHSQSTDYLDVGTN-PRVGTKRYMAPEVLDETIRVD-VFESYKQTDIWALGLVL
WEITRRTI--VN-GIVEEYRPPFFDMV-PSDPSFEEMKKVVCVDQHRPSL-HNR-LHSHP
ILSAIAKIMKECWFQSPSARLTALRVRKSLSKLDQDHDYDIDKLKLDL------------

- **Smad alignment**

>NP_001296671_1_Smad5_Hydra_vulgaris
MASLFSFTSPAVKRLLGWKQG-DEEEKWAEKAIDSLVKKLKKR-KGALEELEKALSNPSS
GN-SKCVTIPRSLDGRLQVSHRKGLPHVIYC--RVWRWPDLQSHHELKPLD-CCEFAFGL
NK-KEVCINPYHYRRVETPVLPPVLVPRQSEYP-RAQAPLPYHMQ-HSHS-GMPHNATLP
GYPLSAYSS-N---TPPPAYMSEDG---GSPRPDPSLMDTDGALSP----------DVTP
ITYQDPLNWCSIAYYELNLRVGEPFHASGT-S--LIIDGFTD-PNTS--SDRFCLGGM-S
NVNR-NSTIENTRRHISKGVHLYYVGGEVFAECLSDSAVFVQSKNCNYHHGFHP-STVCK
IPPQC-T--LKIFNNQEFAQLL-SQSVNHGYEAVYELSK-HCTIRMSFVKGWGAEYH-RQ
GVTSTPCWIEIHLHGPLQWLDKVLTQMGSPQ-NAITSVS
>XM_057436736_1_Smadx_Hydractinia_symbiolongicarpus
MSSLFSFTSPAVKRLLGWKQG-DEEEKWAEKAIDSLVKKLKKR-KGALEELEKALSNPSS
SD-SKCVTIPRSLDGRLQVSHRKGLPHVIYC--RVWRWPDLQSHHELKPLD-CCEYAFSL
NK-KEVCINPYHYRRVETPVLPPVLVPRQSEYP-RAPGPLPYHMQ-HNHT-GMPGNANFP
PPSFSHHPPGG---TPPPAYMSEDG---GSPRPDPNSMDADAAIPP----------DVTP
ITYHEPTNWCSIAYYELNIRVGEPFQASGT-S--LIIDGFTD-PNTS--SDRFCLGGM-S
NVNR-NSTIENTRRHISKGVHLYYVGGEVFAECLSDSAIFVQSRNCNYHHGFHP-TTVCK
IPPQC-T--LKIFNNQEFAQLL-SQSVNHGYEAVYELTK-HCTIRMSFVKGWGAEYH-RQ
DVTSTPCWVEIHLHGPLQWLDKVLTQMGSPR-NPITSVS
>ABC88374_1_Smad1_Nematostella_vectensis
MASLFSFTPPAVKRLLGWKQG-DEEEKWAEKAIESLVKKLKKK-KGALEDLEKALSNPNQ
-Q-SKCVTIARSLDGRLQVSHRKGLPHVIYC--RVWRWPDLQSHHELKPLE-CCEYAFGL
KQ-KEVCINPYHYHRVESPGLPPVLVPRQSEYP-RPPPPLPPPFR-ADDP-PMPYNASFP
---FTNRQNTS---SPSAGYMSEDG---GSPRPEPNAMDVDPVNSPPSISQSEAMSHVTA
VNYQEPLSWCSIAYYELNNRVGELFHAKST-S--LIVDGFTD-PNTTN-SERFCLGLL-S
NVNR-NSTIENTRRHIGKGVHLYYVGGEVYAECLSDSAIFVQSRNCNHSHGFHP-TTVCK
IPSGC-T--LKIFNNQEFAQLL-SQSVNYGFEAVYELTK-MCSIRISFVKGWGAEYH-RQ
DVTSTPCWIEVHLHGPLQWLDKVLSQMGSPR-NPISSVS
>XP_052078142_1_Smad1_Mytilus_californianus
LNSLFSFTSPAVKRLLGWKQG-DEEEKWAEKAVDSLVKKLKKK-KGALEDLEKALSCPGQ
-S-SKCVTIPRSLDGRLQVSHRKGLPHVIYC--RVWRWPDLQSHHELKPLE-CCDFPFSA
KQ-KEVCINPYHYKRVESPVLPPVLVPRYSEYPSGPGQSSLPPFQ-QPEP-SMPQNVSFD
PNGFHQQSQQG---TPPPAYQPHDDNSHNQHSQQTVNLQHGTRRPPPTPMDTPSGVDLQP
VTYQEPQYWCSIVYYELNNRVGEAFHASQT-S--IVVDGFTD-PSNN--ADRFCLGLL-S
NVNR-NSTIENTRRHISKGVHLYYVGGEVFAECLSDSSIFVQSRNCNYHHGFHP-TTVCK
IPPGC-S--LKIFNNQEFAALL-SQSVNHGFEAVYELTK-MCTIRMSFVKGWGAEYH-RQ
DVTSTPCWIEIHLNGPLQWLDKVLTQMGSPH-NPISSVS
>CAH1268124_1_Smad1_Branchiostoma_lanceolatum
MASLFSFTSPAVKRLLGWKQG-DEEEKWAEKAVDSLVKKLKKK-KGAMEELEKALSCPGQ
-P-SKCVTIPRSLDGRLQVSHRKGLPHVIYC--RVWRWPDLQSHHELKPLE-HCNYPFGA
KDTKEVCINPYHYKRVESPVLPPVLVPRHSEF--VPSQTLLPYHN-VPEP-PMPHNATF-
PQSFQNSPGMQSPHTPPPAYMPPGEDQ---NRNNEEQMDTSSQLPPPELRTILECGDVQP
VTYQEPVYWASIAYYELNNRVGEPFHAKSH-S--IIVDGFTD-PSNS--GDRFCLGLI-S
NVNR-NSTIENTRRHIGKGVHLYYVGGEVYAECLSDSAIFVQSRNCNHHHGFHP-TTVCK
IPPGC-S--LKIFNNQEFAQLL-SQSVNHGFEAVYELTK-MCTIRMSFVKGWGAEYH-RQ
DVTSTPCWIEIHLHGPLQWLDRVLTQMGSPH-NPISSVS

>ACU12852_1_Smad1_Paracentrotus_lividus
MASLFSFTSPVVKRLLGWKQG-DEEEKWAEKAVDSLVKKLKKK-KGAMEELERALSRPGE
-L-SKCVTIPRSLDGRLQVSHRKGLPHVIYC--RVWRWPDLQSHHELKALD-CCDYAFGK
KQ-KEVCINPYHYKRVESPVLPPVLVPRHS--ESIPSHSLLPYRSL-PEP-AYPHNATF-
PQSFQQQNSPPTTHTPPPAYMPPDEGGSNQNDNSSTPMDTNPPTQPNQIIA-VERGDVAP
VTYQEPQSWCSIAYYELNNRVGEPFHATTT-S--VIVDGFTD-PSNN--HDRFCLGLL-S
NVNR-NSTIENTRRHIGKGVHLYYVGGEVYAECLSDSSIFVQSRNCNHRHGFHP-TTVCK
IPPGC-S--LKIFNNQEFAALL-SQYVNHGFEMVYELTK-MCTIRMSFVKGWGAEYH-RQ
DVTSTPCWIEIHLNGPLQWLDKVLTQMGSPH-NPISSVS
>KAJ8028250_1_Smad1_Holothuria_leucospilota
MASLFSFTSPVVKRLLGWKQG-DEEEKWAEKAVDSLVKKLKKK-KGAMEELEKALSRPGE
-P-SKCVTIPRSLDGRLQVSHRKGLPHVIYC--RVWRWPDLQSHHELKALD-CCEYAFGK
KQ-KDVCINPYHYKRVESPVLPPVLVPRHSES--VPSHSLLPYRSL-PEP-PFPHNATF-
PQSFQQSQSPQPNMTPPPAYMPPDDK----TKDGSQPMDTSQGSVPPG----INMQDVAP
VTYQEPTNWCSIAYYELNNRVGEPFHASST-S--VIVDGFTN-PQNN--TDRFCLGLL-S
NVNR-NSTIENTRRHISKGVHLYYVGGEVYAECLSDSSIFVQSRNCNYRHGFHP-TTVCK
IPPGC-S--LKIFNNQEFAALL-SQSVNHGFEAVYELTK-MCTIRMSFVKGWGAEYH-RQ
DVTSTPCWIEIHLNGPLQWLDKVLTQMGSPH-NPISSVS
>AAC50790_1_Smad1_Homo_sapiens
VTSLFSFTSPAVKRLLGWKQG-DEEEKWAEKAVDALVKKLKKK-KGAMEELEKALSCPGQ
-P-SNCVTIPRSLDGRLQVSHRKGLPHVIYC--RVWRWPDLQSHHELKPLE-CCEFPFGS
KQ-KEVCINPYHYKRVESPVLPPVLVPRHSEY--NPQHSLLAQFR-NNEP-HMPLNATF-
PDSFQQ-PNSH-PFTPPPAYLPPED---PMTQDGSQPMDT-NMMAPPLPSEI-NRGDVQA
VAYEEPKHWCSIVYYELNNRVGEAFHASST-S--VLVDGFTD-PSNN--KNRFCLGLL-S
NVNR-NSTIENTRRHIGKGVHLYYVGGEVYAECLSDSSIFVQSRNCNYHHGFHP-TTVCK
IPSGC-S--LKIFNNQEFAQLL-AQSVNHGFETVYELTK-MCTIRMSFVKGWGAEYH-RQ
DVTSTPCWIEIHLHGPLQWLDKVLTQMGSPH-NPISSVS
>AAB72180_1_Smad5_Homo_sapiens
MASLFSFTSPAVKRLLGWKQG-DEEEKWAEKAVDALVKKLKKK-KGAMEELEKALSSPGQ
-P-SKCVTIPRSLDGRLQVSHRKGLPHVIYC--RVWRWPDLQSHHELKPLD-ICEFPFGS
KQ-KEVCINPYHYKRVESPVLPPVLVPRHNEF--NPQHSLLVQFR-NNEP-HMPQNATF-
PDSFHQ-PNNT-PFTPPPAYMPPDD---QMGQDNSQPMDTSNNMIPQIMPSI-SSRDVQP
VAYEEPKHWCSIVYYELNNRVGEAFHASST-S--VLVDGFTD-PSNN--KSRFCLGLL-S
NVNR-NSTIENTRRHIGKGVHLYYVGGEVYAECLSDSSIFVQSRNCNFHHGFHP-TTVCK
IPSSC-S--LKIFNNQEFAQLL-AQSVNHGFEAVYELTK-MCTIRMSFVKGWGAEYH-RQ
DVTSTPCWIEIHLHGPLQWLDKVLTQMGSPL-NPISSVS
>NP_001120689_1_Smad9_Homo_sapiens
ISSLFSFTSPAVKRLLGWKQG-DEEEKWAEKAVDSLVKKLKKK-KGAMDELERALSCPGQ
-P-SKCVTIPRSLDGRLQVSHRKGLPHVIYC--RVWRWPDLQSHHELKPLE-CCEFPFGS
KQ-KEVCINPYHYRRVETPVLPPVLVPRHSEY--NPQLSLLAKFRSASEP-LMPHNATY-
PDSFQQPPCSALPPTPPLPYHATEASETQSGQPVDATADRHVVL--------IPNGDFRP
VCYEEPQHWCSVAYYELNNRVGETFQASSR-S--VLIDGFTD-PSNN--RNRFCLGLL-S
NVNR-NSTIENTRRHIGKGVHLYYVGGEVYAECVSDSSIFVQSRNCNYQHGFHP-ATVCK
IPSGC-S--LKVFNNQLFAQLL-AQSVHHGFEVVYELTK-MCTIRMSFVKGWGAEYH-RQ
DVTSTPCWIEIHLHGPLQWLDKVLTQMGSPH-NPISSVS
>XP_001631657_1_Smad3_Nematostella_vectensis
MTSLLPFTPPVVKRLLGWKRG-DEDDKWAEKAVKSLVKKLKKT--GGLEELEKAITNPGV
-A-TKCVTIPRSLDGRLQVSHRKGLPHVIYC--RLWRWPDLQSHHELRPIE-ACEFAFSL
KK-EEVCVNPFHYQRVETPVLPPVLVPRQ-QSDVPHELPILPEY-------TRPENVPFP
TQEPTNSQYHIQPGTPT-DYISDDACSDMSGEHSQQNMQIDQQLSPAPDS--NNLIDAQP
IQYTEPTYWCSISYYEMNTRVGETFHASQP-S--LTVDGFTD-PSSS---DRFCLGLL-S
NINR-NPPIEMTRKHIGKGVRLYYIGGEVFAECLSDSSIFVQSPNCNQRYNWHP-ATVCK
IPPGC-N--LKIFNNQEFAQLL-SQSVNQGFEAVYALTR-MCTIRMSFVKGWGAEYR-RQ
TVTSTPCWIELHLNGPLQWLDKVLTQMGSPS-APCSSVS
>AAC39657_1_Smad2_Homo_sapiens
MSSILPFTPPVVKRLLGWKKG-EQEEKWCEKAVKSLVKKLKKT--GRLDELEKAITTQNC
-N-TKCVTIPRSLDGRLQVSHRKGLPHVIYC--RLWRWPDLHSHHELKAIE-NCEYAFNL
KK-DEVCVNPYHYQRVETPVLPPVLVPRHT--EILTELPPLDDYT-H----SIPENTNF-
PAGIEPQSNY-IPETPPPGYISEDG--ETSDQQLNQSMDTGSPASPTTLSPVNHSLDLQP
VTYSEPAFWCSIAYYELNQRVGETFHASQP-S--LTVDGFTD-PSNS---ERFCLGLL-S
NVNR-NATVEMTRRHIGRGVRLYYIGGEVFAECLSDSAIFVQSPNCNQRYGWHP-ATVCK
IPPGC-N--LKIFNNQEFAALL-AQSVNQGFEAVYQLTR-MCTIRMSFVKGWGAEYR-RQ
TVTSTPCWIELHLNGPLQWLDKVLTQMGSPS-VRCSSMS
>AAL68976_1_Smad3_Homo_sapiens
MSSILPFTPPIVKRLLGWKKG-EQEEKWCEKAVKSLVKKLKKT--GQLDELEKAITTQNV
-N-TKCITIPRSLDGRLQVSHRKGLPHVIYC--RLWRWPDLHSHHELRAME-LCEFAFNM
KK-DEVCVNPYHYQRVETPVLPPVLVPRHT--EIPAEFPPLDDYS-H----SIPENTNF-
PAGIEPQSN--IPETPPPGYLSEDG--ETSDHQMNHSMDAGSP-SPNPMSPAHNNLDLQP
VTYCEPAFWCSISYYELNQRVGETFHASQP-S--MTVDGFTD-PSNS---ERFCLGLL-S
NVNR-NAAVELTRRHIGRGVRLYYIGGEVFAECLSDSAIFVQSPNCNQRYGWHP-ATVCK
IPPGC-N--LKIFNNQEFAALL-AQSVNQGFEAVYQLTR-MCTIRMSFVKGWGAEYR-RQ
TVTSTPCWIELHLNGPLQWLDKVLTQMGSPS-IRCSSVS
>CAH1251607_1_Smad3_Branchiostoma_lanceolatum
MTSMLPFTPPIVKRLLGWKKG-EGEDKWSEKAVKSLVKKLKKT--CGLEELEKSITQQDP
-N-TKCVTIPRSLDGRLQVSHRKGLPHVIYC--RLWRWPDLQSHHELRALE-TCEYAFSL

KR-EEVCVNPYHYQRVETPVLPPVLVPRNPPGEMPAELPPLDDYL-N----SVPENTSY-
PQDVPTQNYS-LPETPPPGYMSEDG--DTNDQSQHNSMVTDSGSSPSPNPSPSQ--DLQP
VTYTEPTFWCSIAYYEMNTRVGETFHASQP-S--LTVDGFTD-PSNS---ERFCLGLL-S
NVNR-NHVIEQTRRHIGKGVRLYYIGGEVFAECLSESSVFVQSPNCNQRYGWHP-ATVCK
IPPGC-N--LKIFNNQEFAQLL-SQSVSQGFEAVYQLTR-MCTIRMSFVKGWGAEYR-RQ
TVTSTPCWIELHLNGPLQWLDKVLTQMGSPR-VPCSSMS
>KAJ8036527_1_Smad3_Holothuria_leucospilota
MSSIIAFTPPIVKRLLGWRKG-NDDDRYSEKAVKSLVRRLKKT--GGIEELERAISTQNS
-Q-TNCITIPESLDGRLQVSHRKRLPHVIYC--QLWRWPDLQSHHELKHVE-NCEYAFSL
RK-DDVCINPYHYLRVQTPVLPPVMVPRQPDIPADIRALPTPTLD-DLSPNTVPDNANY-
PTSMDAQNILPPE-TPPPGYMSEDGDTNDNMEASQNGSEYGSAASPSP----SDSLDMQP
ISYCEPPFWCSITYYEMNRRIGEVFHASQS-S--LTIDGFTD-PSSS---ERFCLGLL-S
NVNR-DPIIEQTRRHIGKGVRLYYIGGEVFAECLSDSSIFVQSPNCNQRYGWHP-ATVCK
IPPGC-N--LKIFNNQEFAAQL-SQSVNQGFEAVYQLTR-MCAIRLSFVKGWGADYR-RQ
TVTSTPCWIELHLNGPLQWLDRVLTQMGSPI-DICTSMS
>KAJ8036411_1_Smad3_Holothuria_leucospilota
LNSIMPFNTPVVKRLLGFIK--DENKKWCEKAVKSLEKKLKRT--GGIDELDKAISTQNS
-N-TKCVTIMRSLDGRLQIHLKKGLPHVIYC--RLWRWPDLVSHHELKPVE-HCEYAFHH
KK-EEVCVNPYHYTRVQTPDLPPIMVPVNTENLATSPPQNTPPAS--SVPNPVPENVNY-
ITSLEPVHALPQE-TPSPGYMSED------SENQDNKMDTSQPPDPNSLTASSPLVDVAP
VFYTEPVSWCSVVYYEKNQHVGEVFHASQP-C--LTIDGFMD-PSSA---ERFCLGIY-S
NASR-DVSIEQTRRHIGKGVTLYYIAGEVFAQCRSDSSIFVQSQNCNRRFGWHP-TTVCK
IPPGC-S--LSVFNNQEFATQL-SQTVSKGYKAVFELTS-VCSIRMSFVKGWGADYQ-RQ
RVNTTPCWVEIQLNGPLQWLDRVLQQMGSPQGDTPGSRS
>XP_012561976_1_Smad3_Hydra_vulgaris
LNSLLNFNAPIVRRLLAWKIG-DGEEHWSEKAVKSLVKKLKKT--GGLEDLEKSISSKGN
NA-TNCVKIIRSLDGRLQVSHRKGLPHVIYC--RLWRWPDLQSHHELRAID-SCEYAFNL
KR-EEVCVNPYHYQRVETPVMPPILVPRGKDLNMKP-EPTLEEF-------PRPENKTI-
SDDFIDNDISNLAFASSPQLMQLDQIQNNSQKSPESSTSPGYMTLPSPNLELSVHDIYDP
VLYEEPEAWCAIAYNELRTRVGDTFHSTKP-V--LTVDGYTD-PSSQ---DRFCLGLL-S
NINR-TEQIELSRRHIGKGVRLYYFGGEVFAECLSNSSIFVQSSNCNRRYGWHP-ATVCK
IPPSC-N--LKIFNNQEFAELL-SQCVPRGFNAVYQMKS-MCMVRLSFVKGWGAEYR-RQ
SITSTPCWIEIRLNGPLKWLDKVLVQMGSSS-DNVNSTT
>XM_057460141_1_Smadx_Hydractinia_symbiolongicarpus
LNSILNFNTSIVRRLLQWKVG-NDEEHWSEKAVKSLVKKLKKSGSGGLEELEKSITTQGQ
GEHTRCVTIPRSLDGRLQVSHRKGLPHVIYV--RLWRWPDLQSHHELRAID-SCQFAFNL
KR-SEVCVNPYHYQRVETPVMPPILVPRKTFETSEPDAPQPDEVH-RPGNVTIPEDESLL
SPVPIASIGANSPSQSSNNAYSQQQQNMVMSQQSPGAMSTGSINIPPNLEVVPEGFDA--
VMYEEPEIWCSILYNELRTRIGEEFSATKP-V--LTVDGYTD-PSSV---DRFCLGLL-S
NINR-TDQIEECRRAIGKGVRLYYFGGEVFAECLSKSSIFVQSSNCNRRYGWHP-ATVCK
IPSGC-N--LKIFNNQEFARLL-ADSVHLGFNAVYQLKS-MCMIRLSFVKGWGAEYR-RQ
TVFSTPCWIEIRLSGPLKWLDKVLVQMDPSV-DKVSSTT
>EDO31382_1_Smadx_Nematostella_vectensis
MANAPTSSLSIVHSLMCHRQG-GESEAFAKRAIESLVKKLKEK-KDELDSLITAITSAGT
HP-SKCVTIQRTLDGRLQVAGRKGFPHVIYA--RIWRWPDLH-KNELRHVK-YCQFAFDL
KC-DSVCVNPFHYERVVSPDIAGLSLSRNSEGGYGSSSQSEPDFRQA----VVPTAHHFP
GGQTIPPPV-----GPPPDMYSMHGAGVE-PGIGHVSQA---------------------
-----PENWCSIAYFELDQQVGEIFKVTSN-CPSVTVDGYVD-PSGG---NRFCLGQL-S
NVHR-TEASERARLHIGKGVQLDVRGGDVWVRCLSEHSVFVQSYYLDREAGRCPGDAVHK
IYPSA-Y--IKVFD---LRALL--PQMGQTSVGVDDLRR-LCILRLSFVKGWGPDYP-RK
SIKETPCWIEIHLHRALQLLDEILITMPINEPRPHDA--
>EDO37590_1_Smadx_Nematostella_vectensis
------------------RQG-GETEKFAKRAIESLVKKLRKK-TDELESLISTITTNGA
QP-SKCVTIQRTLDGRLQVCERKGFPHVIYA--RLWRWPDIQ-KMEMKHLD-FCRFGYDL
KY-ESVCVNPYHYERIRSP---

>ABC88375_1_Smad4_Nematostella_vectensis
QKKAPTSSLSIVLSLMCHRQG-GETEKFAKRAIESLVKKLRKK-TDELESLISTITTNGA
QP-SKCVTIQRTLDGRLQVCERKGFPHVIYA--RLWRWPDIQ-KMEMKHLD-FCRFGYDL
KY-ESVCVNPYHYERIRSP---
------------------------------TDEREKSMH-------------------HP
----------------ISTRNNTTYDTTET-R-----------KA--------------F
RVTR--------SHHVY--THLYFSP------YISTPQAY---------------VFVEN
LHPSL-N--LRSYI--------------------F-------------------------

>CAH1252166_1_Smad4_Branchiostoma_lanceolatum
SSNSVTSNLSIVHSLMCHRQG-GETETFAKRAIESLVKKLKEK-KDELDSLITAITTNGA
HP-SKCVTIQRTLDGRLQVAGRKGFPHVIYA--RIWRWPDLH-KNELKHVK-YCQYAFDL
KA-DSVCVNPYHYERVVSPGIPPSRLVKDEQYNSPGTSDSSQSIQTISRPDSFPHPMLSA
PHSVSSAAPVTNGFTPGPSTLESTTVATQPSTPFRSNTTTWAGNAPPPYWAGDASLANMP
SSRPGPEYWCSIAYFEMDVQVGEIFKVPSS-CPTVTVDGYTD-PSGI---DRFCLGQL-S

```
NVHR-TEASERARLHIGKGVQLDLRGGDVWVRCLSDHAVFVQSYYLDREAGRAPGDAVHK
IYPSA-Y--IKVFDRQCHRQMQQQAATAQAAAAADDLRR-LCILRMSFVKGWGPDYP-RQ
SIKQTPCWIEIHLHRALQLLDEVLHTMPLTGGDPRHLD-
>BAB40977_1_Smad4_Homo_sapiens
ITNTPTSNLSIVHSLMCHRQG-GESETFAKRAIESLVKKLKEK-KDELDSLITAITTNGA
HP-SKCVTIQRTLDGRLQVAGRKGFPHVIYA--RLWRWPDLH-KNELKHVK-YCQYAFDL
KC-DSVCVNPYHYERVVSPGLSGLTLQSNAPSSMMVKDEYVHDFEGQTEG-HSIQTIQHP
PSNRASTETYSTPASGPQPGQQQNGFTGQPATYHHNSTTTWTGSHPPMPPHPHNELAFQP
SNHPAPEYWCSIAYFEMDVQVGETFKVPSS-CPIVTVDGYVD-PSGG---DRFCLGQL-S
NVHR-TEAIERARLHIGKGVQLECKGGDVWVRCLSDHAVFVQSYYLDREAGRAPGDAVHK
IYPSA-Y--IK-------------------------------------------------
---------------------------------------
>KAJ8036600_1_Smad4_Holothuria_leucospilota
LTTAPTSALSIVHSLMCHRQG-GESETFAKRAIESLVKKLKEK-RDELDSLITAITTNGA
HP-SKCVTIQRTLDGRLQVAGRKGFPHVIYA--RIWRWPDLH-KNELKHLK-FCQYAFDL
KC-DSVCVNAYHYERIVSPGIAPSLRPSHPQLPAPPQSQTPYDLQ------NTPSQQTFG
PSGPSTSGTSGAPRRPPPSQQSGDSSLNNGQIGRSASTARWTGSWPSAPIS-GDNMNAAP
LSNQPPEYWCSIAYFELDTQVGEIFKVPAH-CTSVTVDGYVD-PSGM---DRFCLGQL-S
NVHR-VEASERARLHIGKGVELNIEGGDVWVKCLSDHAVFVQSYYLDREAGRAPGDAVHK
VYPGA-F--IKVFDRQCHAQMRSQAATAQAAAAADDLRR-LCILRMSFVKGWGPDYP-RH
SIKETPCWIEIHLHRALQLLDEVLHTMPLMEPRPMD---
>XM_057459701_1_Smadx_Hydractinia_symbiolongicarpus
AVSAPSSNLSIVHSLMCHRQG-GESESFAKRAIESLIKKLKEK-KDELEALITSITTAGA
HP-TKCVTIQRTLDGRLQVAGRKGFPHVIYA--RIWRWPDLH-KNELKHAA-YCQYAFDL
KC-ENVCINPYHYERVVSPDLSTLSISQHTEIPYRMFPPQLKPEIDRGLPSMVPMHVEKH
DMSPHTGGGYSQMAVPPAGHISRHGSASDIHQSGTGGQQSWSAMSPNSSSMTQSSTSGLL
SRHPPPDFWTTIAYYELDQQVGEIFKVPQR-CSSVIVDGYVDASGNG--GNRFCLGQL-S
NVHR-TEASEKALLHIGRGIQLDRRGGDVWVRCLSDQSVFVSSYYLDRQAGRSPGDAVHK
IYPQA-Y--IKVFDRMCFEQMKQQAQAAQAAAAADDLRR-LCLLRLSFVKGWGPDYP-RQ
SIKQTPCWIEVRLHRSLQLLDEVLHQIPIN--EPMPSDR
>XM_057437845_1_Smadx_Hydractinia_symbiolongicarpus
MPPLVSFGMSIVVELLCYRQG-GDSEKFARRAIESLVKKLRKK-SDELESLVIAIKSKGR
QH-SKCVTIPRTLDGRLQVCERKGFPHVIYS--RLFRWPDIH-KMELRHLE-ICQFAFDL
KY-DVVCVNPYHYERISTPSVDPSLFHRHQKFPTGPSRMLMPTYN--GGTSAAPLLPSIA
ISYPPRVPCAADPFSSHHSYHPFHRKINNSKSHIDDRMSTDDPAKTSLPVHIYSREDLYK
SSTTAPIKICAFCCCDLPMHVGYFFRSS--------VNSIID-ADDYLWAHTLCLHWSKS
TANK-NSPVCNFCGHYGKGMQFFCTEYEHFVSCSTCHANFIGKKKLSFQCSNCRSCAVCR
NPLNC-SSCGRCFDERDLLTWLCRNCIGDSHGKFNELSKGHCTIRCRMCEKYAHAICDRV
PAELIEALVEKNLDSPAPWKSATNDTLSSPSKSPVLTSS
>ADW95340_1_Smad6_Paracentrotus_lividus
-----MFRSALVRRLSRSRV--GGNDSGSKAAANSFLKRLKEK---QLELLLEAVESRGG
TQ-TSCVPISKGELGRRTV-----APHVLCC--QLFRWPELKHGSELKRLKFCCETSQEG
DE-ETVCCNPYHISRLCRP-----------ESPPPPYSRIAFERS--KTQ-AEEETPVTS
PVEFGQSTETG---NTPTERRQ--------PYANSSGVD---------------------
ATNGNRRHWCHVAYWEQRTRVGPMYSVFTD-S--VNI--FYDLPH----GDGFCLGLL-N
REGR-PESVAKIRQKIDYGLAMSREEDGVWIYNRSNYALFVNSPALDIPHSRTF--TVHK
LAPGF-S--IKIYDHAKSKLLELMHRESEPPDGPVDPNS----IRISFIKGWGPHYS-RQ
FITSCPCWIELIMTAP-------------PR--------
>AAC82331_1_Smad6_Homo_sapiens
-----MFRSGLVRRL--WRSGGGDEDGSLPRARESLLKRLKER---SLDTLLEAVESRGG
VP-GGCVLVPRA---DLRLGGQPAPPQLLLG--RLFRWPDLQHAVELKPLC-GC-HSFAA
AA-DGVCCNPYHFSRLCGP-----------ESPPPPYSRLSPRDE------YKPLDLSDS
TLSYTETEATNSLITAP-GEFSDAS--------------------------------MSP
-DATKPSHWCSVAYWEHRTRVGRLYAVYDQ-A--VSI--FYDLPQ----GSGFCLGQL-N
LEQR-SESVRRTRSKIGFGILLSKEPDGVWAYNRGEHPIFVNSPTLDAPGGRAL--VVRK
VPPGY-S--IKVFDFER-SGLQ-HAPEPDAADGPYDPNS----VRISFAKGWGPCYS-RQ
FITSCPCWLEILLNNPR----------------------
>CAH1251580_1_Smad6_Branchiostoma_lanceolatum
-----MFRSGLVRRL--WRAGVDEDE-SSGSGSESVLKRLKER---QLELLVQAVESRGG
LE-SGCVHFPRP---DVRIGRRTVAPHVLTC--LLWRWADVQQPHQLKTLM-CCQ-SYGQ
QDPDTVCCNPYHYTRLCGP-----------ESPPPPYSR-Y-PLD-RLRR-LSPEESVSS
STETGASPSLY---PPPPS---------------------------------TSDLSDLP
GQTRKRSHWCSIAYWEHRTRVGRLFAVYDA-S--VNI--FHE-LPHG---DGFCLGLLSS
SESH-AENVVRTRKKIGYGLTLSKEPDGVWAYNRSAHAIFVNSPTLDIPNSRTL--IVRK
IPPGF-S--IKIFDYGRSEMLQ-RTSNSDLLDGPYDPNS----VRISFAKGWGPSYS-RQ
FITSCPCWVEVLLNINR----------------------
>AAL68977_1_Smad7_Homo_sapiens
-----MFRSALVRRL--WRAG-GGELRGEGKAVRSVLKKLKER---QLELLLQAVESRGG
TR-TACLLLPGRLDCRLGPGAPAGLP-LLLC--KVFRWPDLRHSSEVKRLC-CCE-SYGK
INPELVCCNPHHLSRLC---L---------ESPPPPYSRYPMDFL-K----PTADCPDAV
PSSAETGGT---------NYLAPGGLSDSQ-------------------------LLLEP
---GDRSHWCVVAYWEEKTRVGRLYCVQEP-S--LDI--FYDLPQGN----GFCLGQL-N
SDNK-SQLVQKVRSKIGCGIQLTREVDGVWVYNRSSYPIFIKSATLDNPDSRTL--LVHK
VFPGF-S--IKAFDYEK-AYSL-QRPNDHEFM--QQPWT-GFTVQISFVKGWGQCYT-RQ
FISSCPCWLEVIFNSR-----------------------
```

>NP_571431_2_Smad1_Danio_rerio
VTSLFSFTSPAVKRLLGWKQG-DEEEKWAEKAVDALVKKLKKK-KGAMEELERALSCPGQ
-P-SNCVTIPRSLDGRLQVSHRKGLPHVIYC--RVWRWPDLQSHHELKALE-CCEFPFGS
KQ-KDVCINPYHYKRVDSPVLPPVLVPRNSEF--NAKLSMLPRFR-NTEP-PMPQNATF-
PDSFPQQPANALPFTPPPAYMPPEE---PMTQDCPQPMDT-NLLAPNLPLEISNRTDVHP
VAYQEPKHWCSIVYYELNNRVGEAFLASST-S--VLVDGFTD-PSNN--RNRFCLGLL-S
NVNR-NSTIENTRRHIGKGVHLYYVGGEVYAECLSDSSIFVQSRNCNYHHGFHP-TTVCK
IPSRC-S--LKIFNNQEFAELL-AQSVNHGFEAVYELTK-MCTIRMSFVKGWGAEYH-RQ
DVTSTPCWIEIHLHGPLQWLDKVLTQMGSPH-NPISSVS
>NP_571441_3_Smad2_Danio_rerio
MSSILPFTPPVVKRLLGWKKG-EQEEKWCEKAVKSLVKKLKKT--GQLDELEKAITTQNR
-N-TKCVTIPRSLDGRLQVSHRKGLPHVIYC--RLWRWPDLHSHHELRAIE-TCEYAFNL
KK-DEVCVNPYHYQRVETPVLPPVLVPRHT--EILTELPPLDDYT-N----SIPENTNF-
PTGIEPPNNY-IPETPPPGYISEDG--EASDQQMNQSMDTGSPASPSTLSPVNHGMDLQP
VTYSEPAFWCSIAYYELNQRVGETFHASQP-S--LTVDGFTD-PSNS---ERFCLGLL-S
NVNR-NATVEMTRRHIGRGVRLYYIGGEVFAECLSDSAIFVQSPNCNQRYGWHP-ATVCK
IPPGC-N--LKIFNNQEFAALL-AQSVNQGFEAVYQLTR-MCTIRMSFVKGWGAEYR-RQ
TVTSTPCWIELHLNGPLQWLDKVLTQMGSPS-VRCSSMS
>NP_571646_1_Smad3a_Danio_rerio
-MSILPFTPPIVKRLLGWKKG-EQEEKWCEKAVKSLVKKLKKT--GQLDELEKAITTQDV
-N-TKCITIPRSLDGRLQVSHRKGLPHVIYC--RLWRWPDLQSHHELRAIE-LCEFAFHM
KK-DEVCVNPYHYQRVETPVLPPVLVPRHTEIPS--EFPPLDDYS-H----SIPENTIF-
PAGIEPQSNYIPE-TPPPGYISEDGETS--DHQMNRSMDTGSPNSPNPVSPAHNNLDLQP
VTYCEPAFWCSISYYELNQRVGETFHASQP-S--LTVDGFTD-PSNS---ERFCLGLL-S
NVNR-NAAVELTRRHIGRGVRLYYIGGEVFAECLSDSAIFVQSPNCNQRYGWHP-ATVCK
IPPGC-N--LKIFNNQEFAALL-AQSVNQGFEAVYQLTR-MCTIRMSFVKGWGAEYR-RQ
TVTSTPCWIELHLNGPLQWLDKVLTQMGSPS-IRCSSVS
>NP_778258_1_Smad3b_Danio_rerio
-MSILPFTPPIVKRLLGWKKG-EQEEKWCEKAVKSLVKKLKKT--GQLEELEKAITTQNI
-N-TKCITIPRSLDGRLQVSHKKGLPHVIYC--RLWRWPDLQSHHELRAVE-MCEFAFHT
KK-DEVCVNPYHYQRVETPVLPPVLVPRRA--DIPTDFPPLDDY-------SIPENTIF-
PAGIEPPSNYIPE-TPPPGYLSEDG--ETSDHQMSHSMDTGSPTSPNPVSPANSNLDLQP
VTYCESAFWCSISYYELNQRVGETFHASQP-S--LTVDGFTD-PSNA---ERFCLGLL-S
NVNR-NAAVELTRRHIGRGVRLYYIGGEVFAECLSDSAIFVQSPNCNQRYGWHP-ATVCK
IPPGC-N--LKIFNNQEFAALL-AQSVNQGFEAVYQLTR-MCTIRMSFVKGWGAEYR-RQ
TVTSTPCWIELHLNGPLQWLDKVLTQMGSPN-LRCSSVS
>ACA58502_1_Smad4_Danio_rerio
ITNTPTSNLSIVHSLMCHRQG-GESETFAKRAIESLVKKLKEK-KDELDSLITAITTNGA
HP-SKCVTIQRTLDGRLQVAGRKGFPHVIYA--RLWRWPDLH-KNELKHVK-YCQFAFDL
KC-DSVCVNPYHYERVVSPGIPSGLMVKDEDYDGPPSLPSTEGHM-QQEPFSTPASSSAS
SSAFSSIAVGSTTQNPTSSWSRNSNFTPSVPHHQNGHLQHHPPMHPAHYWPVHNEIAFQP
SNHPAPEYWCSIAYFEMDVQVGETFKVPSS-CPIVTVDGYVD-PSGG---DRFCLGQL-S
NVHR-TEAIERARLHIGKGVQLECKGGDVWVRCLSDHAVFVQSYYLDREAGRAPGDAVHK
IYPSA-Y--IKVFDRQCHRQMQQQAATAQAAAAADDLRR-LCILRMSFVKGWGPDYP-RQ
SIKETPCWIEIHMHRALQLLDEVLHTMPIADPTPLD---
>NP_571443_3_Smad5_Danio_rerio
MSSLFSFTSPAVKRLLGWKQG-DEEEKWAEKAVDALVKKLKKK-KGAMEDLEKALSSPGQ
-P-SKCVTIPRSLDGRLQVSHRKGLPHVIYC--RVWRWPDLQSHHELKPLE-VCEYPFGS
KQ-KEVCINPYHYKRVESPVLPPVLVPRHSEF--NPQHSLLVQFR-NNEP-HMPLNATF-
PESFQQHSGGS-SFTPPPAYMPPDE---QMGQDGSQSMETGSSLAPQNMP----RGDVQP
VEYQEPSHWCSIVYYELNNRVGEAYHASST-S--VLVDGFTD-PSNN--KNRFCLGLL-S
NVNR-NSTIENTRRHIGKGVHLYYVGGEVYAECLSDTSIFVQSRNCNYHHGFHP-TTVCK
IPSGC-S--LKIFNNQEFAQLL-AQSVNHGFEAVYELTK-MCTIRMSFVKGWGAEYH-RQ
DVTSTPCWIEVHLHGPLQWLDKVLTQMGSPL-NPISSVS
>NP_001019981_2_Smad6a_Danio_rerio
-----MFRTGLVRRL--WRSG--GPDDWSRRVEEAFLKKLKDK---SLDVLLEAVESQGG
MP-SGCVLVS---QTEVRIGGHLVSPQYLLC--RLFRWPDLRLSSLLKPLC-HCQ-SFRA
EDSQTLCCNPHHYSRLCGPV--------------KDDTPPPPYSHLSPLPEHKPLNSSLP
MLPYIETEA-----TRSAGGLSQD----------------------------YSDASMSP
-SSLAQNHWCNVAYWELRTRVGRLYPVHDA-S--LSI--FYDLPQ----GTGLCLGLL-P
LSPR-STSVQRTRGKIGHGILLSKEPDGVWAYNRSQHPIFVNSPTLE-HHPYLS-LTVRR
VMPGY-S--IKVFDYEKSCQMQ-PASHPVHPEGPYDPNS----VRISFAKGWGPCYS-RQ
FITSCPCWLEILLNNHR----------------------
>NP_001038516_1_Smad6b_Danio_rerio
-----MFRSGLVRRL--WRSGYGEGSRLLSPSVDSLLKRLKEK---TLDTLLEAVESGGM
-P-SDCVMVSRT---ELRLGGHMAPPQLLIC--KLYRWSDLQHTAQLKALC-ECK-SFGA
QD--GVCCNPYHYSRLCGP-----------ESPPPPYSRLSPSEE------HKPLDLSDS
TLSYTETEAAS---SPN-ATQGE-----------------------------FSDASLSP
-DAPKQSHWCNVAYWEHRTRVGRLYTVYQP-A--VSI--FYDLPQGT----GFCLGQL-S
LDQR-SSTVQRTRGKIGYGLLLSKEPDGVWAYNRSQHPIFVNSPTLDVPGSRSL--VVRK
VMPGY-S--IKVFDYER-SSMLRQGAESELLDGPYDPNS----VRISFAKGWGPCYS-RQ
FITSCPCWLEILLNNHR----------------------
>NP_778257_2_Smad7_Danio_rerio
-----MFRSGLVRRL--WRS--RAPVEGEGDADTSILKKIKEK---QLEVLLQAVESRGG
AR-SPCLLLPGKADARLG-QHSYPLPLLLY---KVFRWPDLRHSSELKRLS-CCE-SYGK

```
INPELVCCNPHHMSRLC-------------EL----ESP-PPPYS------RYPTDFLKP
PDSPGSV----------PASTETGGTAYSAPMGFSDSLA-------------LQERGEQP
-------HWCVVAYWEEKTRVGRLYSVQEP-S----LDIFYDLPQGT----GFCLGQL-A
SENK-SQLVQMVRAKIGYGIQLSREADGVWVYNRSCYPIFIKSATLDNPDSRTL--LVHK
VFPGF-S--IKAFDFEKAGSL--QRPNDHEF---SQQPRTGFTVQISFVKGWGQCYT-RQ
FISSCPCWLEVIFNNR----------------------
>NP_001315428_1_Smad9_Danio_rerio
ITSLFSFTSPAVKRLLGWKQG-DEEEKWAEKAVDSLVKKLKKK-KGAMEELEKALSCPGQ
-P-SKCVTIPRSLDGRLQVSHRKGLPHVIYC--RVWRWPDLQSHHELKALD-CCEFPFGS
KQ-KEICINPYHYRRVETPVLPPVLVPRHSEF--NPQHSLLAKFR-NNEP-LMPQNATF-
PDSFPAMPCSS---TPPPPYSMMES---SPSEDV-KPAESSE--NK-LILTA-PQRDLRP
VCYEEPEYWCSVAYYELNNRVGETFHASAR-S--ILVDGFTD-PSNN--KNRFCLGLL-S
NVNR-NSTIEHTRRHIGKGVHLYYVGGEVYAECLSDSSIFVQSRNCNYQHGFHP-TTVCK
IPSGC-S--LKIFNNQLFAQLL-SQSVNHGFEVVYELTK-MCTIRMSFVKGWGAEYH-RQ
DVTSTPCWIEIHLHGPLQWLDKVLTQMGSPH-NPISSVS
>NP_001370321_1_Dwarfin_sma2_Caenorhabditis_elegans
---MINFD--GIKKILKWKQG-DEDENWAKKAIDNLMKKLIKHNKQALENLEFALRCQGQ
QK-TECVTIPRSLDGRLQISHRKALPHVIYC--RVYRWPDLQSHHELKAIE-DCRFCYES
GQ-KDICINPYHYKRVHTGVLPPVLVPRYSEKPPQEVPPTLAKFQ-LSGS-RMPQNVNMA
NVNFTANQFHQ---YNP---------------NGIEEMDTSQKFIPPGVPTCVPFDKV--
---WEEQFWATVSYYELNTRVGEQVKVSST-T--ITIDGFTD-PCIN--GSKISLGLF-S
NVNR-NATIENTRRHIGNGVKLTYVNGSLFAQCESDSAIFVQSSNCNYINGFHS-TTVVK
IANKC-S--LKIFDMEIFRQLL-EDCSRRGFDASFDLQK-MTFIRMSFVKGWGAEYQ-RQ
DVTSTPCWIEIHLHAPLAWLDRVLSTMGPTP-RPISSIS
>NP_001040864_1_Dwarfin_sma4_Caenorhabditis_elegans
ISCVFTSQTPTVNAVLCYQQG-GEDSDFVRKAIESLVKKLKDK-RIELDALITAVTSNGK
QP-TGCVTIQRSLDGRLQVAGRKGVPHVVYA--RIWRWPKVS-KNELVKLV-QCQTSSD-
HP-DNICINPYHYERVVNRIT---------------SADQSLHVE------NSPMKSEYL
GDAGVIDSCSDWPNTPPNGGFAPDQPQLVTPIISDIPIDLNQIYVPTPPQL---------
-----LDNWCSIIYYELDTPIGETFKVSARDHGKVIVDGGMD-PHGE-NEGRLCLGAL-S
NVHR-TEASEKARIHIGRGVELTAH-ADGNISITSNCKIFVRSGYLDYTHGSEYSSKAHR
FTPNESS--FTVFDRWAYMQMLRRSRSSNEAVRADRMRRDFCTIAISFVKAWGDVYQ-RK
TIKETPCWIEVTLHRPLQILDQLLSQFGSS---------
>NP_498493_1_Dwarfin_sma3_Caenorhabditis_elegans
MNGLLHMHGPAVKKLLGWKIG-EDEEKWCEKAVEALVKKLKKKNNGTLEDLECVLANPCT
-N-SRCITIAKSLDGRLQVSHKKGLPHVIYC--RVWRWPDISSPHELRSID-TCSYPYES
SS-KTICINPYHYQRLSRPQ------------GLNSSMPSPQPIS--------SPNTIWQ
SSGSSTASCAS---SPSPSVFSEDGG-------------------------EVQVHQRP
PPFRHPKSWAQITYFELNSRVGEVFKLVNL-S--ITVDGYTN-PSNS--NTRICLGQL-T
NVNR-NGTIENTRMHIGKGIQLDNKEMHIMITNNSDMPVFVQSKNTNLMMNMPL-VKVCR
IPPHS-Q--LCVFEFNLFFQML-EQSCNDS-DGLNELSK-HCFIRISFVKGWGEDYP-RQ
DVTSTPCWLELRLNVPLAYIDQKMKQTPRTNLMEPNSMT
>NP_001368578_1_Daf3_Caenorhabditis_elegans
DPDLFKYDKPAVDKILAYRTN-NIDREFDQKACESLVKKLKDK-KNDLQNLIDVVLSKGT
KY-TGCITIPRTLDGRLQVHGRKGFPHVVYG--KLWRFNEMT-KNETRHVD-HCKHAFEM
KS-DMVCVNPYHYQRYHTPGMPPMNMHTRPQAPHNPGVSHPYSIAPQLNMNPIPQMPQMP
QMPPPLHQGYGMNGHQPPQLSQNHTSQQGSQGQVPNDPPISRPVSGAIIRSSEVLENIMP
APYHDNEKWGTIVYYEKNLQIGEKKCSRGN-F--HVDGGFIC-SE-----NRYSLGLE-P
NPIR-EPVAFKVRKAIVDGIRFSYKKGSVWLQNRMKYPVFVTSGYLDEQSGGLKKDKVHK
VY-GCAS--IKTFGFNVSKQIIRDALLSKQMATMDSLAK-YCCVRVSFCKGFGEAYPERP
SIHDCPVWIELKINIAYDFMDSICQYITNCF-EPLGMED
>NP_492321_1_Daf8_Caenorhabditis_elegans
MDDFPEPTEAMAQKVLEETEG-DGTIYWALKVTR-LLSRVAKK-HQCFEAFYDAVIKGDP
-K-TRCCPAHEKLIGN--------FGRAIMCVLRAFRFPVIRYESQVKSIL-TCRHAFNS
HS-RNVCLNPYHYRWVELPTVPPIIVNKELDYGEPPTEDALDDWNQK----DLKEEQVVA
SWDVPNVTMRGDEISSPREFIPNGNDTISLDQEMMDDSEVSDIMTPIEPRPLRQAGIYEC
VEYEESPSWLKLIYYEEGTMIGEKADVEGH-H--CLIDGFTASRTDSETRSRFSLGWY-N
NPNR-SPQTAEVRGLIGKGVRFYLLAGEVYVENLCNIPVFVQSIGANMKNGFQL-NTVSK
LPPTG-T--MKVFDMRLFSKQL-RTAAEKTYQDVYCLSR-MCTVRVSFCKGWGEHYR-RS
TVLRSPVWFQAHLNNPMHWVDSVLTCMGAPP-RICSSRT
>NP_001255476_1_Daf14_Caenorhabditis_elegans
LPASIN--NPNMP--I----N-D----WLEDAPM-------------------------
---PDCYNVP---------------------------------------------STST
DE-NN---DPFPFSNIS-------------------SQSSLKP--------KTPEKAVVE
VRPTGNEML-----DPEPKYPKEEK----------------------------------
-----P--WCTIFYYELTVRLGKAFEAKVP-T--ITIDGATG-AS-----DECRMSLTSQ
PSSR-NSKSSQIRNTVGAGIQLAYENGELWLTVLTDQIVFVQCPFLNQTLNKPL-KYVFR
LQNKGDQKRMKIFDKEQFEQ---EKTLALGPLTEDERMRIFSNIRVSFCKGFGETYS-RL
KVVNLPCWIEIILHEPADEYDTVF-RINNER-PEIGSRS
>NP_001259992_1_Mad_Drosophila_melanogaster
LGSLFSFTSPAVKKLLGWKQG-DEEEKWAEKAVDSLVKKLKKR-KGAIEELERALSCPGQ
-P-SKCVTIPRSLDGRLQVSHRKGLPHVIYC--RVWRWPDLQSHHELKPLE-LCQYPFSA
KQ-KEVCINPYHYKRVESPVLPPVLVPRHSEF--APGHSMLQ-FN-HAEP-SMPHNVSYS
NSGFNSHSLST---TPPPAYSPSEDGNSNNPNDGGQLLDA-------------QMGDVAQ
VSYSEPAFWASIAYYELNCRVGEVFHCNNN-S--VIVDGFTN-PSNN--SDRCCLGQL-S
```

```
NVNR-NSTIENTRRHIGKGVHLYYVTGEVYAECLSDSAIFVQSRNCNYHHGFHP-STVCK
IPPGC-S--LKIFNNQEFAQLL-SQSVNNGFEAVYELTK-MCTIRMSFVKGWGAEYH-RQ
DVTSTPCWIEIHLHGPLQWLDKVLTQMGSPH-NAISSVS
>AAC38971_1_Med_Drosophila_melanogaster
MPPPPSNALSIVHSLMCHRQG-GESEGFAKRAIESLVKKLKEK-RDELDSLITAITTNGA
HP-SKCVTIQRTLDGRLQVAGRKGFPHVIYA--RIWRWPDLH-KNELKHVK-YCAFAFDL
KC-DSVCVNPYHYERVVSPGLGPSRLVKDEEYPPPPGQPPPQPTQQQPVPHGMPGGPVMA
PPPPPQQAQNPQGNQPPTGVQANTGSAGAQAGAGGGAAGTWTGPGSPQQTPPQQQQQQP
LSRQPPEYWCSIAYFELDTQVGETFKVPSA-KPNVIIDGYVD-PSGG---NRFCLGAL-S
NVHR-TEQSERARLHIGKGVQLDLRGGDVWLRCLSDNSVFVQSYYLDREAGRTPGDAVHK
IYPAC----IKVFDRQCHQQMHSLATNAQAAAAADDLRR-LCILRLSFVKGWGPDYP-RQ
SIKETPCWIEVHLHRALQLLDEVLHAMPIDGPRAAA---
>AAD11458_1_Smad2_Drosophila_melanogaster
---MLPFTPQVVKRLLALKKGNEVEGKWSEKAVKNLVKKIKKN--SQLEELERAISTQNC
-Q-TRCVTVPRSKPAPAGEHLRKGLPHVIYC--RLWRWPDLQSQNELKPLD-HCEYAFHL
RK-EEICINPYHYKKIE----LSILVPK--SLPTPPDSIVDYPLD--NHTHQIPNNTDYN
AAIIRSASLSPPQYTPPPGYMSEDGD----PIDPNDNMNMSRLTPPA---------DAAP
VMYHEPAFWCSISYYELNTRVGETFHASQP-S--ITVDGFTD-PSNS---ERFCLGLL-S
NVNR-NEVVEQTRRHIGKGVRLYYIGGEVFAECLSDSSIFVQSPNCNQRYGWHP-ATVCK
IPPGC-N--LKIFNNQEFAALL-SQSVSQGFEAVYQLTR-MCTIRMSFVKGWGAEYR-RQ
TVTSTPCWIELHLNGPLQWLDRVLTQMGSPR-LPCSSMS
>BAA22841_1_Dad_Drosophila_melanogaster
VSAAPPAQPHRPRSL-AMRQGMDSNSSSCGQSLSALMKQLKR---KQRNELLLAVKSRLD
PP-TKCILIPCKTQTVWE-------PHVTAS--RLFFWRELWNAKELKRLP-TCPAARDC
IY---MCCNPLHWFRILQPE-TESPTPPYQ----RSKMLRLKDADFEEDSQNDAKSAALS
TWSAESTSISNIYK---PALYESVT-----TDGKDHNIN--------------------
-----SQVWCQIAYWEMAHRVGEFFHAKTN-AVNIYTDGIVA-SE----VDSMCLRDLTP
AGNQIHSVVPTARHTVGLGVTLSLENGDVWIYNRGNTTIFVDSPTLSENL-----DRVCK
VMPGC----LKAFE-TNRAELLSMRDHGHHPMGPVDYFS----IKISFGKGWGRDYK-RQ
DIMGCPCWLEVHF-------SHLR---------------
>XP_032236464_1_Smad6_Nematostella_vectensis
LRSIT---LALTRSLLCLKL----LEFTVNKGSESILRRLRDT---ELGLLLKALESRGG
DV-SSCVYFPYGEKVCKRVVH---EPHVVLY--RTFREPNVQSSNELKPLAICSRRDSTG
KR---VCINPHHY--------SEIIQIQSP------TPELQQNLS------PKPHTKQDE
SSGFGS-----------------------NFTETQSTDCSSKKQPNLPLSI---SELFE
STSEWPSAWCVVAYWELNECIGPFYHGHQD-V----INIYETLPK----PKGFCLAGLDR
NQNV-SDGTKRARNHVGFGLQLSREEDGIWIYNRSEYAVFTTFPYVTTTPSASQNITVTK
VPPGF-S--LKIYDYNH------SPATCRTFEGPWKVPE---SVRISFAKGWGASYR-RP
VVTSCPCWLEIHF-------------TVSR--------
```
